# Selective and Additive‐Free Hydrogenation of Nitroarenes Mediated by a DMSO‐Tagged Molecular Cobalt Corrole Catalyst

**DOI:** 10.1002/ejoc.202100073

**Published:** 2021-05-02

**Authors:** Daniel Timelthaler, Wolfgang Schöfberger, Christoph Topf

**Affiliations:** ^1^ Institute of Catalysis (INCA) Johannes Kepler University (JKU) 4040 Linz Austria; ^2^ Institute of Organic Chemistry Johannes Kepler University (JKU) 4040 Linz Austria

**Keywords:** Catalysis, Cobalt, Corroles, Hydrogenation, Nitroarenes

## Abstract

We report on the first cobalt corrole that effectively mediates the homogeneous hydrogenation of structurally diverse nitroarenes to afford the corresponding amines. The given catalyst is easily assembled prior to use from 4‐*tert*‐butylbenzaldehyde and pyrrole followed by metalation of the resulting corrole macrocycle with cobalt(II) acetate. The thus‐prepared complex is self‐contained in that the hydrogenation protocol is free from the requirement for adding any auxiliary reagent to elicit the catalytic activity of the applied metal complex. Moreover, a containment system is not required for the assembly of the hydrogenation reaction set‐up as both the autoclave and the reaction vessels are readily charged under a regular laboratory atmosphere.

## Graphical abstract

A molecularly well‐defined cobalt corrole catalyst was used in the homogeneous pressure hydrogenation of various nitroarenes. The pertinent catalytic system featured broad functional group tolerance and its practicability was verified in the synthesis of a precursor to an anticancer drug (Imatinib).

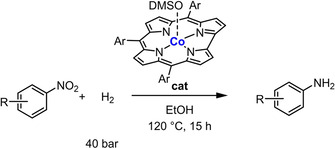

## Introduction

The design of modern hydrogenation catalysts based on earth‐abundant metals relies, to a large extent, on the incorporation of pincer motifs into the catalyst architecture to guarantee a well‐balanced stability‐reactivity relationship of the active species. Especially the reports on pertinent Mn[^1^, ^2^]‐ and Co‐complexes[^3^, ^4^] have ignited a new major wave of research in the field of base‐metal‐centered redox catalysis.

**Figure 1:**
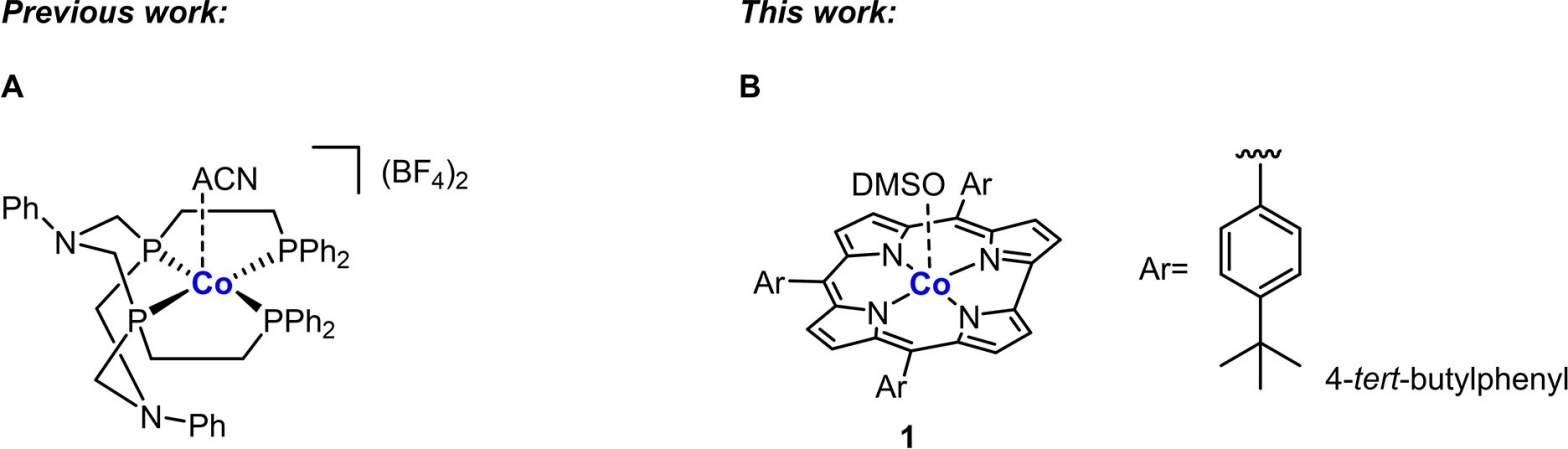
**(A)** Molecular structure of a well‐defined Co‐P^2C−PPh2^_2_N^Ph^_2_ coordination compound with a labile apical acetonitrile (ACN) ligand.^15^ The catalytic activity of this cobalt complex hinges upon the addition of a substoichiometric amount of KOH (10 mol %). **(B)** Structural drawing of the macrocyclic cobalt corrole complex **1** that is a catalyst in its own right and which was successfully employed in the rare homogeneous hydrogenation of nitro compounds to afford amines without the addition of any auxiliary reagents; the appendant dimethylsulfoxide (DMSO) serves as a dummy ligand for an incoming substrate.

Effective donor atom sets in the ligand framework of potent cobalt hydrogenation catalysts are PNP,^[5]^ NNN,^[6]^ CNC,^[7]^ NNP,^[8]^ and NNS^[9]^ constellations. By contrast, tridentate ligands that spread out a tripodal geometry around the metal center might also be capable of catalyzing transformation with gaseous hydrogen.^[10]^ However, cobalt complexes with bidentate ligands containing N, P, and NHC donors have also been successfully implemented in various transformations.^[11]^ Strikingly, certain cobalt‐bisphosphine combinations enable challenging and highly sought‐after enantioselective hydrogenation reactions of enamides and *α*,*β*‐unsaturated carboxylic acids.^[12]^ Homoleptic cobalt complexes that allow for organic transformations with H_2_ gas are rare, yet the early K_3_[Co(CN)_5_] was demonstrated to effect the low‐pressure‐hydrogenation of conjugated C=C double bonds,^[13]^ whereas an anthracene‐tethered cobaltate was shown to catalyze the hydrogenation of olefins, imines, and ketones.^[14]^ Quite recently, Zhang and Wen with coworkers communicated a cobalt/tetraphosphine‐based homogeneous catalytic protocol that relies on the formidable P^2C−PPh2^_2_N^Ph^_2_ ligand which fixes the Co‐center in a square planar environment (Figure 1).^[15]^ However, the respective full‐fledged catalyst that eventually facilitates the hydrogenation of polar double bonds and *N*‐heteroarenes is only obtained upon the addition of high amounts of KOH (10 mol %).

With respect to the precedence of catalytically active cobalt complexes in which the active Co‐ions are firmly anchored in the cavity of a macrocyclic ring, certain cobalt corrole assemblies have been reported to function as efficient electrocatalysts for water splitting^[16]^ and redox‐transformations involving molecular oxygen^[17]^ as well as mediators that bring about the highly relevant electroreduction of CO_2_.[^18^, ^19^] Furthermore, metallocorroles found application as components for gas sensors.^[20]^ However, quite remarkably, the application of these macrocyclic metal complexes in reduction processes using cheap and widely abundant molecular hydrogen has escaped attention so far. This circumstance together with the observation of a PPh_3_‐tagged Co‐corrole to function as selective sensor for nitrite and nitrate ions^[21]^ inspired us to test congeneric complex **1** (Figure 1) for its ability to catalyze the hydrogenation of structurally analogous nitroarenes. Traditionally, this atom‐efficient catalytic transformation is well achieved through 3d‐metal‐based heterogeneous catalysis whereas related homogeneous protocols significantly lag behind in development.^[22]^ For the first time, we herein demonstrate that Co‐corrole **1** represents a decent homogeneous catalyst for the reduction of nitroarenes to the corresponding aniline derivatives using gaseous H_2_.

## Results and Discussion

Our studies commenced with the synthesis of an A_3_‐tris(4‐*tert*‐butylphenyl)corrole according to a standard literature procedure^[23]^ whereby we chose the bulky *tert*‐butyl substituent by virtue of its ability to confer considerable stability on the full‐fledged catalyst. The thus‐obtained macrocycle was then metalated with cobalt(II) acetate^[24]^ to afford the DMSO‐tagged cobalt corrole complex **1**. Both syntheses and in‐depth characterization of this metal complex class have been performed and reported recently by the groups of Paolesse, Gros, and Kadish.^[25]^ Therein, the authors stress the aptitude of the ligated dimethylsulfoxide moiety to readily dissociate from the Co‐center already at room temperature and this fact clearly renders the appendant DMSO an ideal placeholder for the incoming nitroarene substrates.

**Scheme 1:**
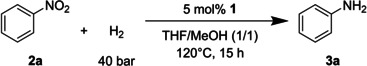
Hydrogenation of nitrobenzene as benchmark reaction for catalyst **1**. Compound **3 a** was isolated in almost quantitative yield as its hydrochloride salt.

Initially, we probed the catalytic activity of Co‐corrole **1** in the homogeneous hydrogenation of nitrobenzene **2 a** (40 bar H_2_, 120 °C) in a mixture of THF and MeOH (1/1 by volume) using a catalyst loading of 5 mol % (Scheme 1). We chose this particular solvent mix in order to provide maximum solubility for the reaction components. To our delight, complete conversion of the model substrate was observed after a period of 15 h. GC‐MS analysis of the reaction mixture then revealed almost quantitative formation of projected aniline **3 a** (99 %) albeit with concomitant generation of minute quantities of *N*‐methylaniline as by‐product (1 %). The latter is presumably formed through single methylation of **3 a** by the solvent MeOH which obviously functioned as an alkylation agent in this case.

We then started a systematic condition evaluation in order to establish an optimized reaction parameter set for the given nitrobenzene hydrogenation. A solvent variation study soon revealed that the use of a protic reaction medium is mandatory for the catalytic activity of complex **1** since all tested common non‐protic solvents including THF, MTBE, 1,4‐dioxane, DMSO, toluene, and *n*‐heptane resulted in full collapse of the catalytic activity. Interestingly, when the reaction was performed under neat conditions, higher substrate conversions were achieved compared to the non‐protic solvents case. Yet, the overall yields could not compete with those obtained for reactions that were run in protic reaction media and amongst the latter, EtOH clearly outperformed its congeners MeOH, *i*‐PrOH, *n*‐BuOH, and H_2_O (Table S1). Expectedly, the use of alcohols bearing longer alkyl chains such as *n*‐BuOH gave rise to higher portions of the above‐mentioned *N*‐alkylaniline. The use of EtOH as solvent was therefore encouraged considering the fact that (industrial) chemical processes should generally strive for replacing petroleum‐based chemicals with analogues that are also accessible through biomass‐valorization in order to comply with the principles of green chemistry.^[26]^

In order to substantiate the homogeneous nature of the introduced Co‐corrole‐based nitroarene hydrogenation, we eventually performed a Maitlis’ filtration experiment.^[27]^ For this purpose, the standard nitrobenzene hydrogenation reaction (120 °C, 40 bar H_2_) was interrupted after 2 h whereas the reaction mixture was then filtrated through a PTFE filter (pore size 0.2 *μ*m) under inert conditions. After that, the clear filtrate was again pressurized with H_2_ (40 bar) while the catalytic transformation was hereafter re‐enacted and allowed to run for another 13 h until completion. In accordance with our expectations, the filtration did not affect the catalyst performance in any way.

Furthermore, we also conducted the nitrobenzene hydrogenation in the presence of a related soluble cobalt tetrakis(4‐*tert*‐butyl)porphyrine complex which was synthesized according to a published procedure^[28]^ and strikingly, this compound was completely inactive under the standard reaction conditions. Hence, we conclude that the ligating macrocycle effectively prohibits the reduction of the metal cation to cobalt (nano)particles that are effective hydrogenation catalysts in their own right.^[29]^

Deterred by the rather high catalyst loading (5 mol %) we then aimed for reducing the latter whilst still maintaining full substrate conversion. We soon found out that most of the nitro substrates were fully converted into the desired anilines at 120 °C and 40 bar H_2_ after a period of 15 h using a loading of only 2 mol % of catalyst **1** (vide infra). As a notable positive side effect of the applied lower catalyst amount, we observed reduced formation of the aforementioned detrimental alkylated anilines.

Further variations of the physical reaction parameters resulted in a marked decrease of the catalytic activity of the given system. Application of lower temperatures (80 °C, 100 °C, and 110 °C, respectively) led to incomplete nitrobenzene conversion and accordingly the yield of aniline was significantly diminished. Similar adverse effects were observed upon reduction of the H_2_ pressure from 40 bar to 20 bar (Table S2) and on stopping the reaction after 2 h, 6 h, and 10 h, respectively (Table S3).

With the intention to provide the cobalt central atom of complex **1** in a low oxidation state to foster the hydrogenation process,^[12a]^ we conducted the nitrobenzene reduction in the presence of solid reducing agents such as Zn, Mn, and Mg. Rewardingly, the pertinent reaction also proceeded well without external reductants and moreover, the catalytic system did not benefit in any way from the addition of acids or bases (Table S4). If ammonia was added to a solution of **1** in ethanol, formation of the known bis(ammine) cobalt corrole complex was observed^[25b]^ which, however, proved to be inactive in the target transformation. These experimental findings provided compelling reasons that the given Co‐catalyzed nitroarene hydrogenation is best performed without any additives.

Subsequently, a variety of functionalized substrates **2 a**, **y** was subjected to pressure hydrogenation in the presence of catalyst **1** (Table 1) and H_2_ gas. On completion of the catalytic transformation, the initially formed anilines **3 a**, **y** were isolated as their hydrochloride salts **4 a**, **y** through precipitation from the reaction mixture upon addition of etheric HCl solution.

**Table 1:** Products obtained upon homogeneous hydrogenation of nitrobenzenes effected by complex 1.^[a]^

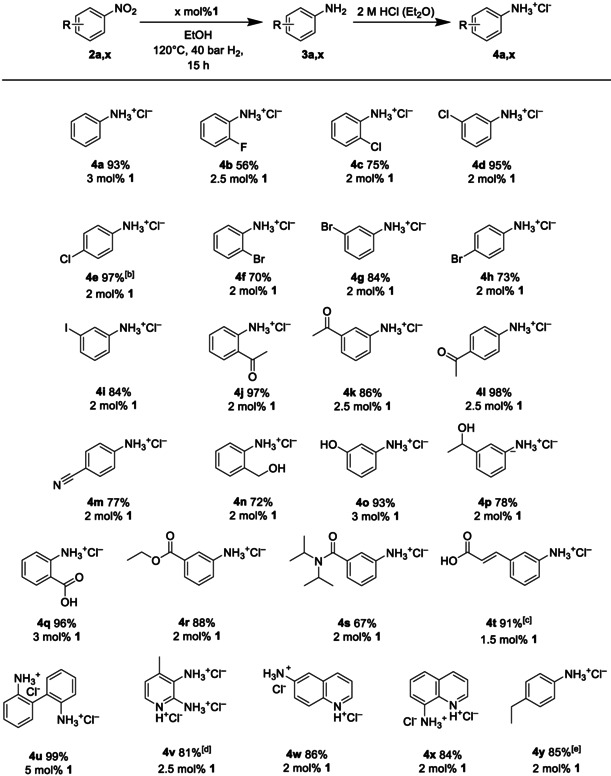

[a] Reaction conditions: nitro compound **2 a**, **x** (0.25 mmol), loading of catalyst **1** as indicated, ethanol (1.5 mL), 120 °C, 40 bar H_2_, and reaction time of 15 h. [b] Additionally, upscaling to 1 mmol substrate was performed with 2 mol % of **1** (88 % isolated yield **4 e** was obtained). [c] Formation of the corresponding saturated amino‐tagged cinnamic acid was observed (4 % as determined by ^1^H NMR), *i. e*. 1,4‐hydrogenation occurred to a minor extent. [d] 4‐Methyl‐3‐nitropyridin‐2‐amine was used as the substrate. [e] 1‐Ethynyl‐4‐nitrobenzene was used as substrate.

Halogenated nitrobenzenes **2 b**, **i** were neatly converted into the anilines and quite remarkably, hydrodehalogenation products were not observed in the GC‐MS spectrum. In addition, nitroarene **2 e** served as test substrate for performing the catalytic hydrogenation applying a higher substrate loading (1 mmol). In this particular case 88 % isolated yield **4 e** were obtained versus 97 % when applying the standard 0.25 mmol substrate amount. The three nitro acetophenones **2 j**, **l** as well as cyano‐tagged **2 m** were selectively hydrogenated at the NO_2_ group with excellent to good yields without competing reductive transformation of the other reducible of the substrates. The presence of hydroxy groups in compounds **2 n**, **p** is also readily accommodated by the given protocol. However, while phenolic substrate **2 o** gave rise to almost quantitative yield of the aniline hydrochloride **4 o** (93 %), the hydroxyalkyl‐congeners furnished products **4 n** and **4 p** in medium 72 % and 78 % yield, respectively.

Ethyl ester **2 r** was exclusively reduced at the nitro group albeit with concomitant transesterification if an alcohol other than EtOH was used as the solvent. Delightfully, our metallocorrole‐based system is also well‐compatible with acidic CO_2_H motifs that usually hamper related hydrogenation protocols.^[10]^ In this context, benzoic acid derivative **2 q** was smoothly transformed into **4 q** when the catalyst amount was slightly increased to 3 mol % instead of the standard 2 mol % loading. As a further notable asset, we did not observe formation of the corresponding ethyl ester although EtOH is present in large excess during the catalytic process. In order to curb the extent of 1,4‐hydrogenation in cinnamic acid derivatives like **2 t**, the catalyst loading had to be decreased to 1.5 mol % but still, it was not possible to fully suppress the Michael reduction of this substrate.

In cases with two NO_2_ groups having to be reduced within the same molecule, such as 2,2′‐dinitrobiphenyl **2 u**, the catalyst amount must be increased accordingly (5 mol %) to achieve quantitative yield of salt **4 u**.

The broad applicability of complex **1** as nitro‐hydrogenation catalyst was further underpinned by the fact that the complex even copes with *N*‐heterocyclic compounds featuring the pyridine or the quinoline core; products **4 v**, **x** were all obtained in good yields exceeding 80 %. Obviously, the catalytic Co center in **1** is not blocked by the sp^2^‐N donors of these heterocycles.

Substrate **2 y** with a *para* ethynyl group was fully hydrogenated to yield the ethyl‐tagged salt **4 y** with good yield. Interestingly, if the parent nitrobenzene was equipped with a vinyl group in the *meta* position, the C=C bond remained intact during the hydrogenation process (vide infra).

**Scheme 2:**
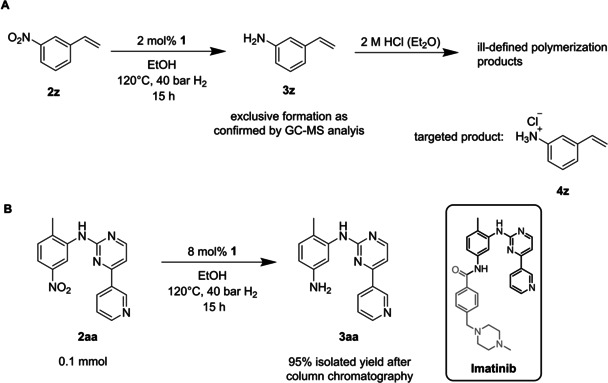
**(A)** Selective homogeneous hydrogenation of nitrostyrene **2 z** and attempted isolation of **3 z** as the HCl salt **4 z. (B)** Synthesis of the Imatinib precursor **3 aa** using molecular H_2_ and complex **1** as catalyst.

We further included challenging 3‐nitrostyrene **2 z** and heterocycle **2 aa** to the substrate panoply. Although catalyst **1** sharply discriminated between the NO_2_ group and the exocyclic C=C double bond in **2 y**, *i. e*. no 3‐ethylaniline was detected in the GC‐MS chromatogram, we were not able to isolate and characterize a pristine portion of 3‐aminostyrene **3 z**, neither in the free amine form nor as its HCl salt **4 z** since compound **3 z** is notoriously prone to rapid polymerization (Scheme 2A).

Amine **3 aa** is an immediate precursor to the anticancer drug Imatinib and its laboratory synthesis usually entails the use of pyrophoric Raney nickel^[30]^ or waste‐producing stoichiometric reagents such as alkaline Na_2_S_2_O_431_ or SnCl_2_/HCl.^[32]^ On the contrary, cobalt corrole **1** permits access to an atom‐efficient way for the manufacture of **3 aa** using cheap H_2_ gas (Scheme 2B).

## Conclusion

We herein introduced a simple and robust catalytic protocol for the rare homogeneous hydrogenation of nitroarenes effected by a well‐accessible Co‐corrole complex. This macrocyclic compound tolerates a broad array of functional groups and does not require any additives such as hydrides or bases to develop catalytic activity. Since the pertinent catalyst is readily handled under open‐flask conditions, the tedious and time‐consuming in‐ and outfeed of the autoclave between an inert‐gas‐filled glovebox and the ordinary laboratory atmosphere is advantageously omitted. The utility of the given metallocorrole as hydrogenation catalyst was further demonstrated in the synthesis of an anticancer drug precursor.

## Experimental Section

**General Information**. All chemicals were purchased from Merck (including Sigma‐Aldrich), Fluorochem, Acros Organics, Alfa Aesar, VWR, Roth, TCI, or Chem Lab and used as received without further purification. The catalytic hydrogenation reactions were carried out in a 300 mL Parr autoclave whereas the used H_2_ gas (5.0 purity) was purchased from Linde Gas GmbH. Practical work under inert conditions was performed in a LABmaster Pro glovebox from Braun filled with argon (6.0 purity from Linde Gas GmbH). GC‐MS analyses were carried out on a Shimadzu GC‐MS QP‐2020 using helium (5.0 purity from Linde Gas GmbH) as carrier gas. UV‐Vis absorption spectra were recorded on a Shimadzu UV‐1800 spectrophotometer and high‐resolution mass spectrometry was performed on a Thermo Fisher Scientific LTQ Orbitrap XL. NMR data were collected on a Bruker Avance III 300 MHz or 500 MHz spectrometer. Spectra for the various cores were recorded as follows: 300 MHz for ^1^H NMR and 75.5 MHz for ^13^C{^1^H} NMR on the 300 MHz spectrometer or 500 MHz for ^1^H NMR, 125.8 MHz for ^13^C{^1^H} NMR and 470.5 MHz for ^19^F NMR on the 500 MHz device. Chemical shifts are listed in parts per million (ppm) on the delta scale (δ). Axis calibration was performed using the residual nondeuterated solvent for ^1^H NMR as a reference.

**Safety Statement Concerning High Pressure Hydrogenations**. The hydrogen container (200 bar, 50 L) is placed in a tapping‐unit‐equipped safety storage cabinet. The vessel is attached to a control panel that allows for fine adjustment of the desired H_2_ pressure used for the hydrogenation reaction. The charging procedure of the autoclave is performed under an efficient fume hood with a built‐in hydrogen sensor which is electronically connected to a magnetic valve that instantaneously stops the gas supply in case of any H_2_ leakage that might occur during the filling procedure. Furthermore, both an optical and an acoustical alarm signal is triggered as soon as free H_2_ is detected inside the hood.

**Standard Protocol for the Catalytic Hydrogenation of Nitroarenes**. A 4 mL glass vial was initially charged with a magnetic stirring bar, complex **1** (2.5–12.5 μmol), and the respective nitroarene (0.25 mmol). After that, the solvent (1.5 mL) was added and the reaction vessel was sealed with a needle‐penetrated septum cap. The glass vials were then placed in a drilled Al plate with a capacity to accommodate seven vessels. Hereafter, the Al inlet was transferred into the autoclave which was then tightly sealed and flushed three times with hydrogen (30 bar) before being pressurized to the definite value. The reaction vessel was then placed on a stirring plate and heated to the required temperature. On completion of the catalytic transformation, the autoclave was cooled to room temperature whereupon the H_2_ pressure was released. Subsequently, solvent (1 mL) was added to each reaction vials and the thus‐diluted reactions solutions were stirred on air for a period of 30 min in order to drive off excess hydrogen. Finally, an aliquot of 50 *μ*L was taken from each vial, mixed with 0.5 mL of methanol (0.5 mL), and analyzed by GC‐MS.

**Standard Procedure for the Isolation of the Hydrogenation Products**. The ethanolic reaction solution as obtained from the catalytic hydrogenation experiment was transferred into a 50 mL round‐bottom flask whereupon the solvent was removed *in vacuo*. The residuals were taken up in dichloromethane and an excess of 2 M HCl solution in diethyl ether was added to initiate prompt formation of a precipitate. The suspension was then filtered and the collected solid was washed with dichloromethane until the draining filtrate appeared colorless. Eventually, the thus‐obtained hydrochloride salt was dried in a desiccator over silica gel.

**Alternative Procedure for the Isolation of the Salts 4 c, 4 p, 4 s, and 4 y**. Since these products proved to be partially or completely soluble in dichloromethane, a different isolation method was elaborated. First, the ethanolic reaction solution from the hydrogenation experiment was evaporated to dryness and after that, 3 M aqueous HCl solution (2 mL) was added. The thus‐obtained suspension was filtrated and the clear aqueous filtrate was then evaporated to dryness leaving behind the hydrochloride salt.

## Conflict of interest

The authors declare no conflict of interest.

## Supporting information

As a service to our authors and readers, this journal provides supporting information supplied by the authors. Such materials are peer reviewed and may be re‐organized for online delivery, but are not copy‐edited or typeset. Technical support issues arising from supporting information (other than missing files) should be addressed to the authors.

Supplementary

## References

[1] S. Elangovan , C. Topf , S. Fischer , H. Jiao , A. Spannenberg , W. Baumann , R. Ludwig , K. Junge , M. Beller , J. Am. Chem. Soc. 2016, 138, 8809–8814. 27219853 10.1021/jacs.6b03709

[2a] S. Elangovan , J. Neumann , J.-P. Sortais , K. Junge , C. Darcel , M. Beller , Nat. Commun. 2016, 7, 12641; 27708259 10.1038/ncomms12641 PMC5059641

[2b] K. Azouzi , A. Bruneau-Voisine , L. Vendier , J.-B. Sortais , S. Bastin , Catal. Commun. 2020, 142, 106040.

[3a] A. Mukherjee , Milstein , ACS Catal. 2018, 8, 11435–11469;

[3b] W. Liu , B. Sahoo , K. Junge , M. Beller , Acc. Chem. Res. 2018, 51, 1858–1869. 30091891 10.1021/acs.accounts.8b00262

[4] M. Hapke , G. Hilt , (Eds.) Cobalt Catalysis in Organic Synthesis: Methods and Reactions, Wiley-VCH: Weinheim, 2020.

[5a] G. Zhang , B. L. Scott , S. K. Hanson , Angew. Chem. Int. Ed. 2012, 51, 12102–12106; 10.1002/anie.201206051 23042754

[5b] S. Rosler , J. Obenauf , R. Kempe , J. Am. Chem. Soc. 2015, 137, 7998–8001; 26080036 10.1021/jacs.5b04349

[5c] J. Yuwen , S. Chakraborty , W. W. Brennessel , ACS Catal. 2017, 7, 3735–3740;

[5d] J. Schneekönig , B. Tanner , H. Hornke , M. Beller , K. Junge , Catal. Sci. Technol. 2019, 9, 1779–1783.

[6a] Q. Knijnenburg , A. D. Horton , H. v. d. Heijden , T. M. Kooistra , D. G. H. Hetterscheid , J. M. M. Smits , B. de Bruin , P. H. M. Budzelaar , A. W. Gal , J. Mol. Catal. A 2005, 232, 151–159;

[6b] S. Monfette , Z. R. Turner , S. P. Semproni , P. J. Chirik , J. Am. Chem. Soc. 2012, 134, 4561–4564; 22390262 10.1021/ja300503k

[6c] J. Chen , C. Chen , C. Ji , Z. Lu , Org. Lett. 2016, 18, 1594–1597. 26974555 10.1021/acs.orglett.6b00453

[7a] R. P. Yu , J. M. Darmon , C. Milsmann , G. W. Margulieux , S. C. E. Stieber , S. DeBeer , P. J. Chirik , J. Am. Chem. Soc. 2013, 135, 13168–13184; 23968297 10.1021/ja406608u PMC3799879

[7b] K. Tokmic , C. R. Markus , L. Thu , A. R. Fout , J. Am. Chem. Soc. 2016, 138, 11907–11913. 27569420 10.1021/jacs.6b07066

[8] D. Srimani , A. Mukherjee , A. F. G. Goldberg , G. Leitus , Y. Diskin-Posner , L. J. W. Shimon , Y. Ben David , D. Milstein , Angew. Chem. Int. Ed. 2015, 54, 12357–12360; 10.1002/anie.201502418 25914240

[9] P. Puylaert , A. Dell'Acqua , F. El Ouahabi , A. Spannenberg , T. Roisnel , L. Lefort , S. Hinze , S. Tin , J. G. de Vries , Catal. Sci. Technol. 2019, 9, 61–64.

[10] T. J. Korstanje , J. Ivar van der Vlugt , C. J. Elsevier , B. de Bruin , Science 2015, 350, 298–302. 26472903 10.1126/science.aaa8938

[11a] S. Sandl , T. M. Maier , N. P. van Leest , S. Kröncke , U. Chakraborty , S. Demeshko , K. Koszinowski , B. de Bruin , F. Meyer , M. Bodensteiner , C. Herrmann , R. Wolf , A. Jacobi von Wangelin , ACS Catal. 2019, 9, 7596–7606;

[11b] Z. Shao , R. Zhong , R. Ferracioli , Y. Li , Q. Liu , Chin. J. Chem. 2019, 37, 1125–1130;

[11c] Z. Wei , Y. Wang , Y. Li , R. Ferraccioli , Q. Liu , Organometallics 2020, 39, 3082–3087.

[12a] M. R. Friedfeld , H. Y. Zhong , R. T. Ruck , M. Shevlin , P. J. Chirik , Science 2018, 360, 888–892; 29798879 10.1126/science.aar6117

[12b] X. Du , Y. Xiao , J.-M. Huang , Y. Zhang , Y.-N. Duan , H. Wang , C. Shi , G.-Q. Chen , X. Zhang , Nat. Commun. 2020, 11, 3239–3249; 32591536 10.1038/s41467-020-17057-z PMC7319995

[12c] H. Zhong , M. Shevlin , P. J. Chirik , J. Am. Chem. Soc. 2020, 142, 5272–5281. 32064867 10.1021/jacs.9b13876

[13] J. Kwiatek , I. L. Mador , J. K. Seyler , J. Am. Chem. Soc. 1962, 84, 304–305.

[14] D. Gärtner , A. Welther , B. R. Rad , R. Wolf , A. Jacobi von Wangelin , Angew. Chem. Int. Ed. 2014, 53, 3722–3726; 10.1002/anie.201308967 24616276

[15] Y.-N. Duan , X. Du , Z. Cui , Z. Zeng , Y. Liu , T. Yang , J. Wen , X. Zhang , J. Am. Chem. Soc. 2019, 141, 20424–20433. 10.1021/jacs.9b11070 31791120

[16] X. Li , H. Lei , J. Liu , X. Zhao , S. Ding , Z. Zhang , X. Tao , W. Zhang , W. Wang , X. Zheng , R. Cao , Angew. Chem. Int. Ed. 2018, 57, 15070–15075; 10.1002/anie.201807996 30242949

[17a] K. M. Kadish , L. F. Frémond , Z. Ou , J. Shao , C. Shi , F. C. Anson , F. Burdet , C. P. Gros , J.-M. Barbe , R. Guilard , J. Am. Chem. Soc. 2005, 127, 5625–5631; 15826202 10.1021/ja0501060

[17b] W. Schöfberger , F. Faschinger , S. Chattopadhyay , S. Bhakta , B. Mondal , A. A. W. J. Elemans , S. Müllegger , S. Tebi , R. Koch , F. Klappenberger , M. Paszkiewicz , J. V. Barth , E. Rauls , H. Aldahhak , W. G. Schmidt , A. Dey , Angew. Chem. Int. Ed. 2016, 55, 2350–2355; 10.1002/anie.201508404 PMC4949709 26773287

[17c] H. C. Honig , C. B. Krishnamurthy , I. Borge-Duràn , M. Tasior , D. T. Gryko , I. Grinberg , L. Elbaz , J. Phys. Chem. C 2019, 123, 26351–26357.

[18] R. De , S. Gonglach , S. Paul , M. Haas , S. S. Sreejith , P. Gerschel , U.-P. Apfel , T. H. Vuong , J. Rabeah , S. Roy , W. Schöfberger , Angew. Chem. Int. Ed. 2020, 59, 10527–10534; 10.1002/anie.202000601 PMC7540269 32281187

[19] S. Gonglach , S. Paul , M. Haas , F. Pillwein , S. S. Sreejith , S. Barman , R. De , S. Müllegger , P. Gerschel , U.-P. Apfel , H. Coskun , A. Aljabour , P. Stadler , W. Schöfberger , S. Roy , Nat. Commun. 2019, 10, 3864. 31455766 10.1038/s41467-019-11868-5 PMC6711975

[20] J.-M. Barbe , G. Canard , S. Brandés , F. Jérôme , G. Dubois , R. Guilard , Dalton Trans. 2004, 1208–1214. 15252662 10.1039/b316706b

[21a] S. Yang , Y. Wo , M. Meyerhoff , Anal. Chim. Acta 2014, 843, 89–96; 25150700 10.1016/j.aca.2014.06.041 PMC4143778

[21b] S. Yang , M. Meyerhoff , Electroanalysis 2013, 25 (12), 2579–2585. 25422577 10.1002/elan.201300400 PMC4239217

[22a] D. Formenti , F. Ferretti , F. Scharnagl , M. Beller , Chem. Rev. 2018, 119, 2611–2680; 30516963 10.1021/acs.chemrev.8b00547

[22b] A. Corma , P. Serna , Science 2006, 313, 332–334; 16857934 10.1126/science.1128383

[22c] R. V. Jagadeesh , A.-E. Surkus , H. Junge , M.-M. Pohl , J. Radnik , J. Rabeah , H. Huan , V. Schünemann , A. Brückner , M. Beller , Science 2013, 342, 1073–1076; 24288327 10.1126/science.1242005

[22d] P. Zhou , L. Jiang , F. Wang , K. Deng , K. Lv , Z. Zhang , Sci. Adv. 2017, 3, e1601945. 28232954 10.1126/sciadv.1601945 PMC5315448

[23] B. Koszarna , D. Gryko , J. Org. Chem. 2006, 71, 3707–3717. 16674040 10.1021/jo060007k

[24] S. Nardis , F. Mandoj , M. Stefanelli , R. Paolesse , Coord. Chem. Rev. 2019, 388, 360–405.

[25a] W. Osterloh , V. Quesneau , N. Desbois , S. Brandès , W. Shan , V. Blondeau-Patissier , R. Paolesse , C. Gros , K. Kadish , Inorg. Chem. 2019, 59, 595–611; 31825638 10.1021/acs.inorgchem.9b02855

[25b] V. Quesneau , W. Shan , N. Desbois , S. Brandès , Y. Rousselin , M. Vanotti , V. Blondeau-Patissier , M. Naitana , P. Fleurat-Lessard , E. van Caemelbecke , K. Kadish , C. Gros , Eur. J. Inorg. Chem. 2018, 38, 4265–4277;

[25c] W. Osterloh , N. Desbois , V. Quesneau , S. Brandès , P. Fleurat-Lessard , Y. Fang , V. Blondeau-Patissier , R. Paolesse , C. Gros , K. Kadish , Inorg. Chem. 2020, 59, 8562–8579; 32452674 10.1021/acs.inorgchem.0c01037

[25d] X. Jiang , W. Shan , N. Desbois , V. Quesneau , S. Brandès , E. Caemelbecke , W. Osterloh , V. Blondeau-Patissier , C. Gros , K. Kadish , New J. Chem. 2018, 42, 8220–8229.

[26] P. Anastas , N. Eghbali , Chem. Soc. Rev. 2010, 39, 301–312. 20023854 10.1039/b918763b

[27] J. E. Hamlin , K. Hirai , A. Millan , P. M. Maitlis , J. Mol. Catal. 1980, 7, 543–544.

[28] A. Alonso-Castro , J. Zapata-Morales , A. Hernández-Munive , N. Campos-Xolalpa , S. Pérez-Gutiérrez , C. Pérez-González , Bioorg. Med. Chem. 2015, 23, 2529–2537. 25863493 10.1016/j.bmc.2015.03.043

[29a] P. Büschelberger , E. Reyes-Rodriguez , C. Schöttle , J. Treptow , C. Feldmann , A. Jacobi von Wangelin , R. Wolf , Catal. Sci. Technol. 2018, 8, 2648–2653;

[29b] H. Dai , H. Guan , ACS Catal. 2018, 8, 9125–9130;

[29c] D. Timelthaler , C. Topf , J. Org. Chem. 2019, 84, 11604–11611. 31454242 10.1021/acs.joc.9b01544

[30] A. Kompella , B. R. K. Adibhatla , P. R. Muddasani , S. Rachakonda , V. K. Gampa , P. K. Dubey , Org. Process Res. Dev. 2012, 16, 1794–1804.

[31] A. S. Ivanov , S. V. Shishkov , Monatsh. Chem. 2009, 140, 619–623.

[32] M. Patoliya , G. J. Kharadi , J. Chem. 2013, 2013, 1–7.

