# Supplementary material for: Selective and Additive‐Free Hydrogenation of Nitroarenes Mediated by a DMSO‐Tagged Molecular Cobalt Corrole Catalyst
Source: European J Org Chem. 2021 May 2;2021(14):2114–20. doi: 10.1002/ejoc.202100073 (PMC8252576; doi:10.1002/ejoc.202100073)
Supplement: Supplementary file 1 — Supplementary [file EJOC-2021-2114-s001.pdf]

European Journal of Organic Chemistry

Supporting Information

Selective and Additive-Free Hydrogenation of Nitroarenes Mediated by a DMSO-Tagged Molecular Cobalt Corrole Catalyst

Daniel Timelthaler, Wolfgang Schöfberger, and Christoph Topf*

Supporting Information

Table of Contents

Table S1: Effect of the Reaction Medium on the Composition of the Reaction Mixture Obtained Through Co-Corrole-Catalyzed Hydrogenation of Nitrobenzene S2

Table S2: Effect of the Catalyst Loading, Temperature, and H_2 pressure on the Composition of the Reaction Mixture Obtained Through Co-Corrole-Catalyzed Hydrogenation of Nitrobenzene S3

Table S3: Effect of the Reaction Time on the Composition of the Reaction Mixture Obtained Through Co-Corrole-Catalyzed Hydrogenation of Nitrobenzene S4

Table S4: Effect of Various Additives on the Composition of the Reaction Mixture Obtained Through Co-Corrole-Catalyzed Hydrogenation of Nitrobenzene S5

Experimental Procedures S6

References S16

Figures S1–S90. ^{1}H-, ^{13}C{^{1}H}-, ^{19}F NMR, and HR-MS Spectra S17

Table S1: Effect of the Reaction Medium on the Composition of the Reaction Mixture Obtained Through Co-Corrole-Catalyzed Hydrogenation of Nitrobenzene[a]

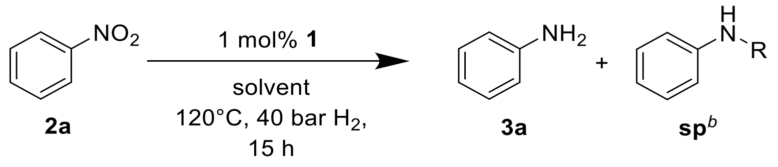

Entry	Solvent	Amount 2a / %[c]	Amount 3a / %[c]	Amount sp / %[c]
1	MeOH	83	17	<1
2	EtOH	36	64	<1
3	*i*-PrOH	71	29	<1
4	*n*-BuOH	62	29	9
5	H_2O	71	29	<1
6	THF/EtOH (1/1 by volume)	70	24	5
7	THF/H_2O (1/1 by volume)	67	33	<1
8	THF	100	0	0
9	EtOAc	100	0	0
10	1,4-Dioxane	100	0	0
11	Acetone	100	0	0
12	DMSO	100	0	0
13	Toluene	100	0	0
14	*n*-Heptane	100	0	0
15	neat	83	17	0

[a]Reaction conditions: nitrobenzene **2a** (0.2 mmol), complex **1** (1 mol%), solvent (1.5 mL), 120 °C, 40 bar H_2, 15 h reaction time. [b]Side product **sp** is the *N*-alkylated aniline derived from the organic rest of the solvent alcohol. [c]Amounts are based on the peak ratios of the respective components in the chromatogram as obtained upon GC-MS measurement.

Table S2: Effect of the Catalyst Loading, Temperature, and H_2 pressure on the Composition of the Reaction Mixture Obtained Through Co-Corrole-Catalyzed Hydrogenation of Nitrobenzene[a]

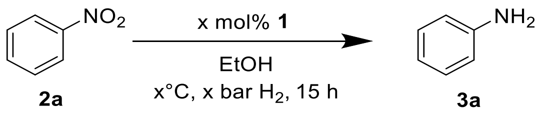

Entry	Catalyst loading / mol%	Temperature / °C	H_2 pressure / bar	Amount 2a / %[b]	Amount 3a / %[b]
1	1	120	40	27	73
2	2	120	40	<1	>99
3	2	110	40	19	81
4	1	100	40	88	12
5	2	100	40	51	49
6	1	80	40	>99	<1
7	2	80	40	80	20
8	1	120	20	79	21
9	2	120	20	6	94
10	0	120	40	100	0
11	2	120	Ar[c]	100	0

[a]Reaction conditions: nitrobenzene **2a** (0.1 mmol), complex **1** (loading as indicated), solvent (1.5 mL), temperature and H_2 pressure as indicated, 15 h reaction time. [b]Amounts are based on the peak ratios of the respective components in the chromatogram as obtained via GC-MS measurement. [c]The reaction was performed in a pressure tube under an inert gas atmosphere.

Table S3: Effect of the Reaction Time on the Composition of the Reaction Mixture Obtained Through Co-Corrole-Catalyzed Hydrogenation of Nitrobenzene[a]

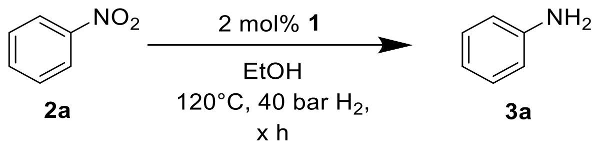

Entry	Reaction time / h	Amount 2a / %[b]	Amount 3a / %[b]
1	2	82	18
2	6	54	46
3	10	27	73
4	15	<1	>99

[a]Reaction conditions: nitrobenzene **2a** (0.1 mmol), **1** (2 mol%), in 1.5 mL of solvent, 120 °C, 40 bar H_2, reaction time as indicated. [b]Amounts are based on the peak ratios of the respective components in the chromatogram as obtained via GC-MS measurement.

Table S4: Effect of Various Additives on the Composition of the Reaction Mixture Obtained Through Co-Corrole- Catalyzed Hydrogenation of Nitrobenzene[a]

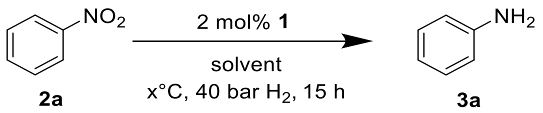

Entry	Solvent	Temperature / °C	Additive	Amount 2a / %[b]	Amount 3a / %[b]
1	THF/MeOH (1/2)	100	Zn (20 mol%)	75	25
2	THF/MeOH (1/2)	100	Zn (20 mol%) NH_3 in H_2O (18 M, 20 µL)	96	4
3	THF/MeOH (1/2)	100	Zn (20 mol%) NH_4CO_3 (200 mol%)	98	2
4	THF/MeOH (1/2)	100	Zn (20 mol%) NH_4Cl (200 mol%)	84	16
5	THF/MeOH (1/2)	100	Zn (20 mol%) NaOH (100 mol%)	97	3
6	THF/MeOH (1/2)	100	Zn (20 mol%) K_2CO_3 (100 mol%)	97	3
7	THF/MeOH (1/2)	100	Zn (20 mol%) KOtBu (100 mol%)	97	3
8	THF/MeOH (1/2)	100	Zn (20 mol%) $NaHCO_3$ (100 mol%)	95	5
9	THF/EtOH (1/20)	100	Zn (20 mol%) $KHSO_4$ (20 mol%)	44	56
10	THF/EtOH (1/20)	100	Zn (20 mol%) $Zn(OTf)_2$ (20 mol%)	25	75
11	THF/EtOH (1/20)	100	Zn (20 mol%) $Al(OTf)_3$ (20 mol%)	35	65
12	THF/EtOH (1/20)	100	Zn (20 mol%) $ZnCl_2$ (20 mol%)	35	65
13	THF/EtOH (1/20)	100	Zn (20 mol%) $KHSO_4$ (20 mol%)	44	56
14	THF/EtOH (1/20)	100	Mn (20 mol%)	25	75
15	THF/EtOH (1/20)	100	Mg (20 mol%)	31	64
16[c]	THF/EtOH (1/20)	120	CH_3COOH (20 mol%)	29	71
17[c]	THF/EtOH (1/20)	120	CH_3COOH (40 mol%)	37	63
18[c]	THF/EtOH (1/20)	120	CH_3COOH (100 mol%)	38	62
19[c]	THF/EtOH (1/20)	120	TFA (20 mol%)	56	44
20[c]	THF/EtOH (1/20)	120	TFA (40 mol%)	41	59
21[c]	THF/EtOH (1/20)	120	TFA (100 mol%)	32	68

[a]Reaction conditions: nitrobenzene **2a** (0.1 mmol), **1** (2 mol%), solvent (1.5 mL), temperature and as indicated, 40 bar H_2 pressure, reaction time: 15 h. [b]Amounts is based on the peak ratio of the respect component in the chromatogram as obtained through GC-MS measurement. [c]1 mol% of **1** was used.

Experimental Procedures

*Synthesis of free-base and mono-DMSO cobalt corrole (**1**).* The free-base tris-(4-*tert*-butylphenyl) corrole was synthesized according to a procedure reported by Gryko and co-workers[1] whereas the ensuing metalation was carried out following the guidelines developed by the groups of Paolesse, Gros, and Kadish.[2] Catalyst **1** was synthesized upon mixing 191 mg of free-base corrole (0.274 mmol) with 76 mg of cobalt acetate tetrahydrate (0.30 mmol) in DMSO (30 mL) and hereafter the solution was stirred under a nitrogen atmosphere at 80°C for a period of 50 min. After cooling to room temperature, the solution was poured into cold aqueous 0.8 M NaCl solution (100 mL) upon which the resulting precipitate was collected on a frit, washed five times with water, and eventually dried in a desiccator over silica gel.

*(4-tBuPh)$_3$CorCo(DMSO) (**1**).* Dark red solid: 214 mg (0.258 mmol, 94%). Analytical data: UV-vis [CH_2Cl_2 with 1% DMSO; λ_{max}, nm (ε x 10^{-3}, L mol^{-1} cm^{-1})]: 394 (76.5), 564 (13.0). ^{1}H NMR (300 MHz, $CDCl_3$, 20 °C): δ = 8.40 (d, *J* = 3.85 Hz, 2H), 8.12 -7.59 (m, 16H*), 6.55 (s, 2H), 1.56 (s, 18H), 1.53 (s, 9H), 0.74 (s, 6H) ppm; HR-MS (ESI+): m/z: calcd. for $C_{49}H_{47}CoN_4$: 750.3127 [M – DMSO]$^{\bullet+}$; found: 750.3134 [M – DMSO]$^{\bullet+}$. *2 β-pyrrole signals are overlapping with phenyl-H signals.

*Synthesis of 1-(3-nitrophenyl)ethan-1-ol (**2p**).* The title compound was synthesized according to a published literature method.[3] In a 50 mL round-bottom flask were placed 3-nitroacetophenone (1.20 g, 6.66 mmol) which was then dissolved in methanol (10 mL) upon which the solution was cooled down to 0°C using an ice bath. Then, sodium borohydride (0.52 g, 19 mmol) was added in small portions over a period of 10 min. Hereafter, the reaction mixture was allowed to reach room temperature whereas stirring was continued for a further 17 h. The solvent was then removed *in vacuo* while the crude was subsequently extracted thrice with EtOAc. The combined organic phases were washed with brine, dried with Na_2SO_4, and finally evaporated to dryness leaving behind the desired alcohol.

*1-(3-Nitrophenyl)ethan-1-ol (**2p**).* Brown oil: 959 mg (5.74 mmol, 86%). Analytical data: ^{1}H NMR (300 MHz, $CDCl_3$, 20 °C): δ = 8.24 (s, 1H), 8.11 (d, *J* = 8.15 Hz, 1H), 7.70 (d, *J* = 7.66 Hz, 1H), 7.51 (t, *J* =

7.92 Hz, 1H), 5.01 (q, J = 6.44 Hz, 1H), 2.20 (s, 1H), 1.52 (d, J = 6.50 Hz, 3H) ppm; $^{13}C\{^1H\}$ NMR (75.5 MHz, $CDCl_3$, 20 °C): δ = 148.5, 148.0, 131.7, 129.6, 122.5, 120.5, 69.5, 25.6 ppm.

*Synthesis of 3-nitrostyrene (**2y**).* The olefin was synthesized according to a literature method.[4] A 50 mL round-bottom flask was charged with 1-(3-nitrophenyl)ethan-1-ol (600 mg, 3.59 mmol) and hydroquinone (4.5 mg, 41 μmol) which functioned as a stabilizer. To the solid mixture was added 85% orthophosphoric acid (5 ml) while the suspension was then heated to 80°C and agitated for 2 h. Hereafter, water was added (20 mL) and the mixture was extracted thrice with EtOAc whereas the combined organic phases were then washed with brine, dried with Na_2SO_4, and evaporated to dryness. Finally, the product mixture was purified by column chromatography (silica, heptane/EtOAc, 10/1 by volume) yielding the product as a colorless oil which was stored at -80 °C to avoid oligomerization.

*3-Nitrostyrene (**2y**).* Colorless oil: 241 mg (1.62 mmol, 45 %). Analytical data: ^1H NMR (300 MHz, $CDCl_3$, 20 °C): δ = 8.25 (t, J = 1.86 Hz, 1H), 8.10 (dd, J_1 = 8.18 Hz, J_2 = 1.33 Hz, 1H), 7.70 (d, J = 7.74 Hz, 1H), 7.49 (t, J = 7.95 Hz, 1H), 6.77 (dd, J_1 = 17.58 Hz, J_2 = 10.91 Hz, 1H), 5.89 (d, J = 17.56 Hz, 1H), 5.44 (d, J = 10.50 Hz, 1H) ppm; $^{13}C\{^1H\}$ NMR (75.5 MHz, $CDCl_3$, 20 °C): δ = 148.7, 139.4, 134.9, 132.2, 129.6, 122.5, 121.0, 117.2 ppm.

*Synthesis of ethyl-3-nitrobenzoate (**2r**).* The ester was synthesized according to a literature procedure.[5] In a 50 mL Schlenk flask, dry 3-nitrobenzoylchloride (500 mg, 2.69 mmol) was placed to which dry ethanol (10 mL, 170 mmol) was added slowly over a period of 1 min. The resulting mixture was kept at reflux for 6 h under a dry nitrogen atmosphere whereupon the excess ethanol was distilled of, leaving behind the crude as a yellow-white oil. The title compound was obtained as an off-white solid after column chromatography (silica, heptane/CH_2Cl_2, 1/1 by volume).

*Ethyl-3-nitrobenzoate (**2r**).* Off-white solid: 474 mg (2.42 mmol, 90%). Analytical data: ^1H NMR (300 MHz, $CDCl_3$, 20 °C): δ = 8.88 (t, J = 1.90 Hz, 1H), 8.46-8.36 (m, 2H), 7.68 (t, J = 8.00 Hz, 2H), 4.47 (q, J = 7.14 Hz, 2H), 1.46 (t, J = 7.15 Hz, 3H) ppm; $^{13}C\{^1H\}$ NMR (75.5 MHz, $CDCl_3$, 20 °C): δ = 164.5, 148.3, 135.3, 132.2, 129.6, 127.3, 124.5, 61.9, 14.3 ppm.

*Synthesis of N,N-diisopropyl-3-nitrobenzamide (**2s**).* The amide was prepared following a literature protocol.[6] In a 50 mL Schlenk-flask, dry 3-nitrobenzoylchloride (500 mg, 2.69 mmol) was suspended in dry diethyl ether (15 mL) whereupon the mixture was cooled to 0°C using an ice bath. After that, dry diisopropylamine (1 mL, 7.09 mmol) was slowly added by syringe upon which the mixture was allowed to reach room temperature. Under a dry argon gas atmosphere, the flask content was then stirred for 18 h at this temperature whereupon 5% aqueous HCl solution (5 mL) was added. Subsequently, the organic phase was washed twice with 5% aqueous HCl solution, once with brine, once with 5% NaOH solution, and again once with brine in that order. Eventually, the organic phase was dried with Na_2SO_4, filtered over cotton, and then evaporated to dryness leaving behind the title compound.

*N,N-Diisopropyl-3-nitrobenzamide (**2s**).* White solid: 666 mg (2.65 mmol, 99%). Analytical data: ^{1}H NMR (300 MHz, $CDCl_3$, 20 °C): δ = 8.24-8.17 (m, 1H), 8.17-8.12 (m, 1H), 7.67-7.51 (m, 2H), 3.63 (s, 2H), 1.34 (d, *J* = 70.35 Hz, 12H) ppm; ^{13}C{^{1}H} NMR (75.5 MHz, $CDCl_3$, 20 °C): δ = 168.2, 148.2, 140.3, 131.8, 129.9, 123.6, 121.0, 51.4, 46.3, 20.7 ppm; HR-MS (ESI+): m/z: calcd. for $C_{13}H_{19}N_2O_3$: 131.1390 $[M + H]^+$; found: 131.1395 $[M + H]^+$.

*Anilinium hydrochloride (**4a**).* The title compound was synthesized and isolated through the standard procedures described above whereas 31.2 mg (0.253 mmol) of **2a** were used as starting material. White solid: 30.4 mg (0.235 mmol, 93% yield). Analytical data: ^{1}H NMR (300 MHz, D_2O, 20 °C): δ = 7.59-7.49 (m, 3H), 7.44-7.37 (m, 2H) ppm; ^{13}C{^{1}H} NMR (75.5 MHz, D_2O, 20 °C): δ = 130.1, 129.8, 129.1, 122.8 ppm; HR-MS (ESI+): m/z: calcd. for $C_6H_8N^+$: 94.0651 $[M – Cl]^+$; found: 94.0649 $[M – Cl]^+$.

*2-Fluoroanilinium hydrochloride (**4b**).* The title compound was synthesized and isolated through the standard procedures described above whereas 34.1 mg (0.242 mmol) of **2b** were used as starting material. Off-white solid: 19.8 mg (0.134 mmol, 56% yield). Analytical data: ^{1}H NMR (300 MHz, D_2O, 20 °C): δ = 7.53-7.40 (m, 2H), 7.40-7.24 (m, 2H) ppm; ^{13}C{^{1}H} NMR (75.5 MHz, D_2O, 20 °C): δ = 155.4 (d, *J* = 248 Hz), 130.5 (d, *J* = 7.71 Hz), 125.4 (d, *J* = 3.86 Hz), 124.5, 118.4 (d, *J* = 13.81 Hz), 116.6 (d, *J* = 18.00 Hz) ppm; ^{19}F NMR (470.5 MHz, D_2O, 20 °C): δ = -126.5 (s, Ar-F) ppm; HR-MS (ESI+): m/z: calcd. for $C_6H_7FN^+$: 112.0557 $[M – Cl]^+$; found: 112.0555 $[M – Cl]^+$.

2-Chloroanilinium hydrochloride (***4c***). The title compound was synthesized via the standard procedure and the isolation followed the alternative procedure as described above; 39.9 mg (0.253 mmol) of **2c** were used as starting material. White solid: 31.3 mg (0.191 mmol, 75% yield). Analytical data: ^{1}H NMR (300 MHz, D_2O, 20 °C): δ = 7.56-7.52 (m, 1H), 7.49-7.35 (m, 3H) ppm; ^{13}C{^{1}H} NMR (75.5 MHz, D_2O, 20 °C): δ = 130.5, 130.3, 128.5, 127.9, 127.4, 124.5 ppm; HR-MS (ESI+): m/z: calcd. for $C_6H_7ClN^+$: 128.0262 $[M – Cl]^+$; found: 128.0261 $[M – Cl]^+$.

3-Chloroanilinium hydrochloride (***4d***). The title compound was synthesized and isolated through the standard procedures described above whereas 39.2 mg (0.249 mmol) of **2d** were used. White solid: 38.7 mg (0.236 mmol, 95% yield). Analytical data: ^{1}H NMR (300 MHz, D_2O, 20 °C): δ = 7.54-7.44 (m, 3H), 7.37-7.28 (m, 1H) ppm; ^{13}C{^{1}H} NMR (75.5 MHz, D_2O, 20 °C): δ = 134.9, 131.3, 131.1, 129.2, 123.2, 121.4 ppm; HR-MS (ESI+): m/z: calcd. for $C_6H_7ClN^+$: 128.0262 $[M – Cl]^+$; found: 128.0261 $[M – Cl]^+$.

4-Chloroanilinium hydrochloride (***4e***). The title compound was synthesized and isolated through the standard procedures described above whereas 39.2 mg (0.249 mmol) of **2e** were used as starting material. White solid: 39.5 mg (0.241 mmol, 97% yield). Analytical data: ^{1}H NMR (300 MHz, D_2O, 20 °C): δ = 7.49 (d, *J* = 8.71 Hz, 2H), 7.35 (d, *J* = 8.74 Hz, 2H) ppm; ^{13}C{^{1}H} NMR (75.5 MHz, D_2O, 20 °C): δ = 134.4, 130.0, 128.4, 124.5 ppm; HR-MS (ESI+): m/z: calcd. for $C_6H_7ClN^+$: 128.0262 $[M – Cl]^+$; found: 128.0261 $[M – Cl]^+$.

2-Bromoanilinium hydrochloride (***4f***). The title compound was synthesized and isolated through the standard procedures described above whereas 46.7 mg (0.231 mmol) of **2f** were used as starting material. Off-white solid: 33.5 mg (0.161 mmol, 70% yield). Analytical data: ^{1}H NMR (300 MHz, D_2O, 20 °C): δ = 7.76 (d, *J* = 8.00 Hz, 1H), 7.49-7.44 (m, 2H), 7.40-7.30 (m, 1H) ppm; ^{13}C{^{1}H} NMR (75.5 MHz, D_2O, 20 °C): δ = 133.8, 130.3, 129.8, 129.1, 124.3, 116.4 ppm; HR-MS (ESI+): m/z: calcd. for $C_6H_7BrN^+$: 171.9756 $[M – Cl]^+$; found: 171.9759 $[M – Cl]^+$.

3-Bromoanilinium hydrochloride (***4g***). The title compound was synthesized and isolated through the standard procedures described above whereas 50.5 mg (0.250 mmol) of **2g** were used as starting material. White solid: 44.0 mg (0.211 mmol, 84% yield). Analytical data: ^{1}H NMR (300 MHz, D_2O, 20 °C): δ = 7.70-7.64 (m, 1H), 7.63-7.35 (m, 3H) ppm; ^{13}C{^{1}H} NMR (75.5 MHz, D_2O, 20 °C): δ = 132.1, 131.5, 131.5, 125.9, 122.6, 121.8 ppm; HR-MS (ESI+): m/z: calcd. for $C_6H_7BrN^+$: 171.9756 $[M – Cl]^+$; found: 171.9757 $[M – Cl]^+$.

4-Bromoanilinium hydrochloride (***4h***). The title compound was synthesized and isolated through the standard procedures described above whereas 46.7 mg (0.231 mmol) of **2h** were used as starting material. Off-white solid: 35.4 mg (0.170 mmol, 73% yield). Analytical data: ^{1}H NMR (300 MHz, D_2O, 20 °C): δ = 7.71 - 7.64 (m, 2H), 7.33 - 7.27 (m, 2H) ppm; ^{13}C{^{1}H} NMR (75.5 MHz, D_2O, 20 °C): δ = 133.1, 129.2, 124.7, 122.3 ppm; HR-MS (ESI+): m/z: calcd. for $C_6H_7BrN^+$: 171.9756 $[M – Cl]^+$; found: 171.9758 $[M – Cl]^+$.

3-Iodoanilinium hydrochloride (***4i***). The title compound was synthesized and isolated through the standard procedures described above whereas 62.3 mg (0.250 mmol) of **2i** were used as starting material. Off-white solid: 53.8 mg (0.211 mmol, 84% yield). Analytical data: ^{1}H NMR (300 MHz, D_2O, 20 °C): δ = 7.86 (d, *J* = 7.92 Hz,1H), 7.82-7.76 (m, 1H), 7.44-7.36 (m, 1H), 7.28 (t, *J* = 8.02 Hz, 1H) ppm; ^{13}C{^{1}H} NMR (75.5 MHz, D_2O, 20 °C): δ = 138.3, 131.7, 131.5, 131.0, 122.4, 93.9 ppm; HR-MS (ESI+): m/z: calcd. for $C_6H_7IN^+$: 219.9618 $[M – Cl]^+$; found: 219.9619 $[M – Cl]^+$.

2-Acetylbenzenaminium hydrochloride (***4j***). The title compound was synthesized and isolated through the standard procedures described above whereas 40.7 mg (0.246 mmol) of **2j** were used as starting materila. Off-white solid: 40.9 mg (0.238 mmol, 97% yield). Analytical data: ^{1}H NMR (300 MHz, D_2O, 20 °C): δ = 8.16 (d, *J* = 7.81 Hz,1H), 7.72 (t, *J* = 7.34 Hz, 1H), 7.58 (t, *J* = 7.57 Hz, 1H), 2.68 (s, 3H) ppm; ^{13}C{^{1}H} NMR (75.5 MHz, D_2O, 20 °C): δ = 203.7, 135.1, 132.8, 131.1, 128.8, 127.8, 124.6, 27.5 ppm; HR-MS (ESI+): m/z: calcd. for $C_8H_{10}NO^+$: 136.0757 $[M – Cl]^+$; found: 136.0757 $[M – Cl]^+$.

3-Acetylbenzenaminium hydrochloride (***4k***). The title compound was synthesized and isolated through the standard procedures described above whereas 42.2 mg (0.256 mmol) of **2k** were used as starting material. Off-white solid: 37.8 mg (0.220 mmol, 86% yield). Analytical data: ^{1}H NMR (300 MHz, D_2O, 20 °C): δ = 8.10-8.02 (m, 1H), 7.93 (s, 1H), 7.70-7.63 (m, 2H), 2.65 (s, 3H) ppm; ^{13}C{^{1}H} NMR (75.5 MHz, D_2O, 20 °C): δ = 201.9, 138.0, 130.7, 129.3, 127.9, 122.4, 26.3 ppm; HR-MS (ESI+): m/z: calcd. for $C_8H_{10}NO^+$: 136.0757 $[M – Cl]^+$; found: 136.0757 $[M – Cl]^+$.

4-Acetylbenzenaminium hydrochloride (***4l***). The title compound was synthesized and isolated through the standard procedures described above whereas 41.2 mg (0.250 mmol) of **2l** were used as starting material. Off-white solid: 42.1 mg (0.245 mmol, 98% yield). Analytical data: ^{1}H NMR (300 MHz, D_2O, 20 °C): δ = 8.00 (d, *J* = 8.48 Hz, 2H), 7.43 (d, *J* = 8.46 Hz, 2H), 2.58 (s, 3H) ppm; ^{13}C{^{1}H} NMR (75.5 MHz, D_2O, 20 °C): δ = 202.2, 136.0, 135.9, 130.4, 122.6, 26.2 ppm; HR-MS (ESI+): m/z: calcd. for $C_8H_{10}NO^+$: 136.0757 $[M – Cl]^+$; found: 136.0757 $[M – Cl]^+$.

4-Cyanobenzenaminium hydrochloride (***4m***). The title compound was synthesized and isolated through the standard procedures described above whereas 36.9 mg (0.249 mmol) of **2m** were used as starting material. Off-white solid: 29.7 mg (0.192 mmol, 77% yield). Analytical data: ^{1}H NMR (300 MHz, D_2O, 20 °C): δ = 7.84 (d, *J* = 8.50 Hz, 2H), 7.45 (d, *J* = 8.55 Hz, 2H) ppm; ^{13}C{^{1}H} NMR (75.5 MHz, D_2O, 20 °C): δ = 137.1, 134.3, 122.8, 118.8, 110.1 ppm; HR-MS (ESI+): m/z: calcd. for $C_7H_7N_2^+$: 119.0604 $[M – Cl]^+$; found: 119.0603 $[M – Cl]^+$.

2-(Hydroxymethyl)benzenaminium hydrochloride (***4n***). The title compound was synthesized and isolated through the standard procedures described above whereas 39.0 mg (0.255 mmol) of **2n** were used as starting material. Light brown solid: 29.3 mg (0.184 mmol, 72% yield). Analytical data: ^{1}H NMR (300 MHz, D_2O, 20 °C): δ = 7.48-7.31 (m, 4H), 4.74 (s, 2H) ppm; ^{13}C{^{1}H} NMR (500 MHz, D_2O, 30 °C): δ = 133.5, 129.8, 129.6, 128.7, 124.0, 61.0 ppm; HR-MS (ESI+): m/z: calcd. for $C_7H_{10}NO^+$: 124.0757 $[M – Cl]^+$; found: 124.0749 $[M – Cl]^+$.

3-Hydroxybenzenaminium hydrochloride (***4o***). The title compound was synthesized and isolated through the standard procedures described above whereas 34.7 mg (0.250 mmol) of **2o** were used as the starting material. Brown solid: 33.6 mg (0.231 mmol, 93% yield). Analytical data: ^{1}H NMR (300 MHz, D_2O, 20 °C): δ = 7.39 (t, *J* = 8.13 Hz, 1H), 7.01-6.90 (m, 2H), 6.88 (t, *J* = 2.19 Hz 1H) ppm; ^{13}C{^{1}H} NMR (75.5 MHz, D_2O, 20 °C): δ = 156.8, 131.3, 130.8, 116.1, 114.6, 110.1 ppm; HR-MS (ESI+): m/z: calcd. for $C_6H_8NO^+$: 110.0600 $[M – Cl]^+$; found: 110.0599 $[M – Cl]^+$.

3-(1-Hydroxyethyl)benzenaminium hydrochloride (***4p***). The title compound was synthesized via the standard procedure and the isolation followed the alternative procedure as described above; 41.7 mg (0.250 mmol) of **2p** were used as starting material. Brown solid: 33.8 mg (0.195 mmol, 78% yield). Analytical data: ^{1}H NMR (300 MHz, D_2O, 20 °C): δ = 7.57-7.46 (m, 2H), 7.40 (s, 1H), 7.32 (dt, J_1 = 7.16 Hz, J_2 = 1.81 Hz, 1H), 4.95 (q, *J* = 6.53 Hz, 1H), 1.46 (d, *J* = 6.44 Hz, 3H) ppm; ^{13}C{^{1}H} NMR (75.5 MHz, D_2O, 20 °C): δ = 147.5, 130.3, 129.9, 126.3, 121.9, 119.9, 69.0, 23.6 ppm; HR-MS (ESI+): m/z: calcd. for $C_8H_{12}NO^+$: 138.0913 $[M – Cl]^+$; found: 138.0915 $[M – Cl]^+$.

3-Carboxybenzenaminium hydrochloride (***4q***). The title compound was synthesized and isolated through the standard procedures described above whereas 41.5 mg (0.248 mmol) of **2q** were used as starting material. White solid: 41.2 mg (0.237 mmol, 96% yield). Analytical data: ^{1}H NMR (300 MHz, D_2O, 20 °C): δ = 8.09-8.02 (m, 1H), 7.97 (s, 1H), 7.67-7.61 (m, 2H) ppm; ^{13}C{^{1}H} NMR (75.5 MHz, D_2O, 20 °C): δ = 168.9, 131.8, 130.5, 130.3, 130.2, 127.8, 124.0 ppm; HR-MS (ESI+): m/z: calcd. for $C_7H_8NO_2^+$: 138.0550 $[M – Cl]^+$; found: 138.0551 $[M – Cl]^+$.

3-(Ethoxycarbonyl)benzenaminium hydrochloride (***4r***). The title compound was synthesized and isolated through the standard procedures described above whereas 47.5 mg (0.243 mmol) of **2r** were used as starting material. White solid: 43.0 mg (0.213 mmol, 88% yield). Analytical data: ^{1}H NMR (300 MHz, D_2O, 20 °C): δ = 8.13 -8.03 (m, 1H), 7.98 (s, 1H), 7.69-7.61 (m, 2H), 4.38 (q, *J* = 7.05 Hz, 2H), 1.36 (t, *J* = 7.14 Hz, 3H) ppm; ^{13}C{^{1}H} NMR (75.5 MHz, D_2O, 20 °C): δ = 167.3, 131.8, 130.5, 130.4,

129.9, 127.7, 123.6, 62.7, 13.3 ppm; HR-MS (ESI+): m/z: calcd. for $C_9H_{12}NO_2^+$: 166.0863 [M – Cl]$^+$; found: 166.0865 [M – Cl]$^+$.

3-(Diisopropylcarbamoyl)benzenaminium hydrochloride (***4s***). The title compound was synthesized via the standard procedure and the isolation followed the alternative procedure as described above; 62.7 mg (0.251 mmol) of **2s** were used as starting material. White solid: 43.0 mg (0.168 mmol, 67% yield). Analytical data: ^{1}H NMR (300 MHz, D_2O, 20 °C): δ = 7.60 (t, *J* = 7.84, 1H), 7.50-7.40 (m, 2H), 7.35 (s, 1H), 3.82-3.62 (m, 2H), 1.44 (d, *J* = 6.76 Hz, 6H), 1.12 (d, *J* = 6.66 Hz, 6H) ppm; ^{13}C{^{1}H} NMR (75.5 MHz, D_2O, 20 °C): δ = 171.1, 139.1, 130.9, 130.4, 125.9, 123.8, 119.9, 52.3, 46.2, 19.6, 19.4 ppm; HR-MS (ESI+): m/z: calcd. for $C_{13}H_{21}N_2O^+$: 221.1648 [M – Cl]$^+$; found: 221.1653 [M – Cl]$^+$.

3-(2-Carboxyvinyl)benzenaminium hydrochloride (***4t***). The title compound was synthesized and isolated through the standard procedures described above whereas 49.2 mg (0.255 mmol) of **2t** were used as starting material. Brown solid: 46.4 mg (0.232 mmol, 91% yield). Analytical data: ^{1}H NMR (300 MHz, D_2O, 20 °C): δ = 7.78-7.51 (m, 4H), 7.49-7.40 (m, 1H), 6.63-6.49 (m, 1H) ppm; ^{13}C{^{1}H} NMR (75.5 MHz, D_2O, 20 °C): δ = 170.4, 144.1, 135.9, 130.7, 130.6, 128.6, 124.6, 122.3, 119.4 ppm; HR-MS (ESI+): m/z: calcd. for $C_9H_{10}NO_2^+$: 164.0706 [M – Cl]$^+$; found: 164.0709 [M – Cl]$^+$.

[1,1'-Biphenyl]-2,2'-diaminium hydrochloride (***4u***). The title compound was synthesized and isolated through the standard procedures described above whereas 61.9 mg (0.254 mmol) of **2u** were used as starting material. Light-brown solid: 64.4 mg (0.250 mmol, 99% yield). Analytical data: ^{1}H NMR (300 MHz, D_2O, 20 °C): δ = 7.69-7.51 (m, 6H), 7.46 (dd, J_1 = 7.45 Hz, J_2 = 1.55 Hz, 1H) ppm; ^{13}C{^{1}H} NMR (75.5 MHz, D_2O, 20 °C): δ = 131.5, 130.8, 130.1, 129.8, 128.7, 123.6 ppm; HR-MS (ESI+): m/z: calcd. for $C_{12}H_{13}N_2^+$: 185.1073 [M – Cl]$^+$; found: 185.1076 [M – Cl]$^+$.

2,3-Diammonio-4-methylpyridinium hydrochloride (***4v***). The title compound was synthesized and isolated through the standard procedures described above whereas 39.0 mg (0.255 mmol) of **2v** were used as starting material. Red-brown solid: 48.2 mg (0.207 mmol, 81% yield). Analytical data: ^{1}H NMR

(300 MHz, D_2O, 20 °C): δ = 7.26 (d, *J* = 6.46 Hz, 1H), 6.74 (d, *J* = 6.48 Hz, 1H) 2.23 (s, 3H) ppm; $^{13}C\{^1H\}$ NMR (75.5 MHz, D_2O, 20 °C): δ = 144.6, 136.7, 128.6, 124.3, 116.6, 16.9 ppm; HR-MS (ESI+): m/z: calcd. for $C_6H_{10}N_3^+$: 124.0869 $[M – Cl]^+$; found: 124.0868 $[M – Cl]^+$.

6-Ammonioquinolinium hydrochloride (***4w***). The title compound was synthesized and isolated through the standard procedures described above whereas 43.0 mg (0.247 mmol) of **2w** were used as starting material. Brown solid: 46.2 mg (0.213 mmol, 86% yield). Analytical data: ^1H NMR (300 MHz, D_2O, 20 °C): δ = 9.01-8.93 (m, 2H), 8.20-8.13 (m, 1H) 8.00 (dd, J_1 = 8.47 Hz, J_2 = 5.52 Hz, 1H), 7.90-7.84 (m, 2H) ppm; $^{13}C\{^1H\}$ NMR (75.5 MHz, D_2O, 20 °C): δ = 146.2, 142.9, 138.0, 134.7, 129.7, 128.6, 122.4, 122.2, 117.0 ppm; HR-MS (ESI+): m/z: calcd. for $C_9H_{10}N_2^+$: 145.0760 $[M – Cl]^+$; found: 145.0761 $[M – Cl]^+$.

8-Ammonioquinolinium hydrochloride (***4x***). The title compound was synthesized and isolated through the standard procedures described above whereas 43.4 mg (0.249 mmol) of **2x** were used as starting materials. Dark-brown solid: 45.6 mg (0.210 mmol, 84% yield)· Analytical data: ^1H NMR (300 MHz, D_2O, 20 °C): δ = 8.94 (dd, J_1 = 5.14 Hz, J_2 = 1.24 Hz, 1H), 8.83 (dd, J_1 = 8.47 Hz, J_2 = 1.31 Hz, 1H), 7.87 (dd, J_1 = 8.45 Hz, J_2 = 5.19 Hz, 1H), 7.79-7.73 (m, 1H), 7.67 (t, *J* = 7.86 Hz, 1H), 7.58 (dd, , J_1 = 7.52 Hz, J_2 = 1.06 Hz, 1H) ppm; $^{13}C\{^1H\}$ NMR (75.5 MHz, D_2O, 20 °C): δ = 145.1, 145.0, 133.5, 131.8, 129.6, 129.4, 122.7, 121.7, 121.4 ppm; HR-MS (ESI+): m/z: calcd. for $C_9H_{10}N_2^+$: 145.0760 $[M – Cl]^+$; found: 145.0761 $[M – Cl]^+$.

4-Ethylanilinium hydrochloride (***4y)***. The title compound was synthesized via the standard procedure and the isolation followed the alternative procedure as described above; 37.2 mg (0.253 mmol) of 4-ethynyl nitrobenzene **2y** was used. White solid: 34.0 mg (0.216 mmol, 85% yield). Analytical data: ^1H NMR (300 MHz, D_2O, 20 °C): δ = 7.34 (d, *J* = 8.49 Hz, 2H), 7.27 (d, *J* = 8.50 Hz, 2H), 2.60 (q, *J* = 7.62 Hz, 2H), 1.13 (t, *J* = 7.63 Hz, 3H) ppm; $^{13}C\{^1H\}$ NMR (75.5 MHz, D_2O, 20 °C): δ = 146.0, 129.4, 127.0,

122.7, 27.7, 14.8 ppm; HR-MS (ESI+): m/z: calcd. for $C_8H_{12}N^+$: 122.0964 $[M – Cl]^+$; found: 122.0964 $[M – Cl]^+$.

6-Methyl-N¹-(4-(pyridin-3-yl)pyrimidin-2-yl)benzene-1,3-diamine (***3aa***). The title compound was synthesized via the standard hydrogenation procedure described above whereas 31.0 mg (0.101 mmol) of **2aa** were used as starting material. The product **3aa** was isolated on removing the volatiles *in vacuo* and subsequent purification of the crude by column chromatography (silica, EtOAc + 1% MeOH + 5% NEt_3) which yielded the product as a yellow solid. 26.6 mg (0.095 mmol, 95% yield). Analytical data: ^{1}H NMR (300 MHz, $CDCl_3$, 20 °C): δ = 8.25 (s, 1H), 8.70 (s, 1H), 8.49 (d, *J* = 5.12 Hz, 1H), 8.33 (d, *J* = 7.87 Hz 1H), 7.60 (d, *J* = 1.45 Hz, 1H), 7.41 (dd, , J_1 = 7.62 Hz, J_2 = 4.91 Hz, 1H), 7.13 (d, *J* = 5.10 Hz, 1H), 7.05-6.92 (m, 2H), 6.41 (dd, , J_1 = 7.93 Hz, J_2 = 1.88 Hz, 1H), 3.65 (s, 2H), 2.25 (s, 3H) ppm; ^{13}C{^{1}H} NMR (75.5 MHz, $CDCl_3$, 20 °C): δ = 162.7, 160.8, 159.1, 151.6, 148.7, 145.3, 138.0, 134.5, 132.9, 131.2, 123.8, 118.3, 110.8, 108.5, 108.2, 17.4 ppm; HR-MS (ESI+): m/z: calcd. for $C_{16}H_{16}N_5^+$: 278.1400 $[M – H]^+$; found: 278.1407 $[M – H]^+$.

References

(1) Koszarna, B.; Gryko, D. Efficient Synthesis of *meso*-Substituted Corroles in a H_2O-MeOH Mixture. *J. Org. Chem.* **2006**, *71*, 3707-3717.

(2) Osterloh, W.; Quesneau, V.; Desbois, N.; Brandès, S.; Shan, W.; Blondeau-Patissier, V.; Paolesse, R.; Gros, C.; Kadish, K. Synthesis and The Effect of Anions on the Spectroscopy and Electrochemistry of Mono(dimethyl sulfoxide)-Ligated Cobalt Corroles. *Inorg. Chem.* **2019**, *59* (1), 595-611.

(3) Li, X.; Tan, H. Preparation Method of 1-Ethyl-3-Nitrobenzene by Using *m*-Nitroacetophenone, Sodium Borohydride and Iodine. Patent CN 110498744 A, **2019**.

(4) Wan, P.; Davis, M.; Teo, M. Photoaddition of Water and Alcohols to 3-Nitrostyrenes. Structure-Reactivity and Solvent Effects. *J. Org. Chem.* **1989**, *54*, 1354-1359.

(5) Desrousseaux, S.; Bennetau, B.; Morand, J.; Mingotaud, C.; Létard, J.; Montant, S.; Freysz, E. Design of New Fluorinated Bridged Push–Pull Stilbenes and Preparation of LB Films for Second Harmonic Generation in the Blue Domain. *New J. Chem.* **2000**, *24*, 977-985.

(6) Arnold, K.; Batsanov, A.; Davies, B.; Whiting, A. Synthesis, Evaluation and Application of Novel Bifunctional *N,N*-Di-Isopropylbenzylamineboronic Acid Catalysts for Direct Amide Formation Between Carboxylic Acids and Amines. *Green Chem.* **2008**, *10*, 124-134.

^{1}H-, ^{13}C{^{1}H}-, ^{19}F NMR and HR-MS Spectra

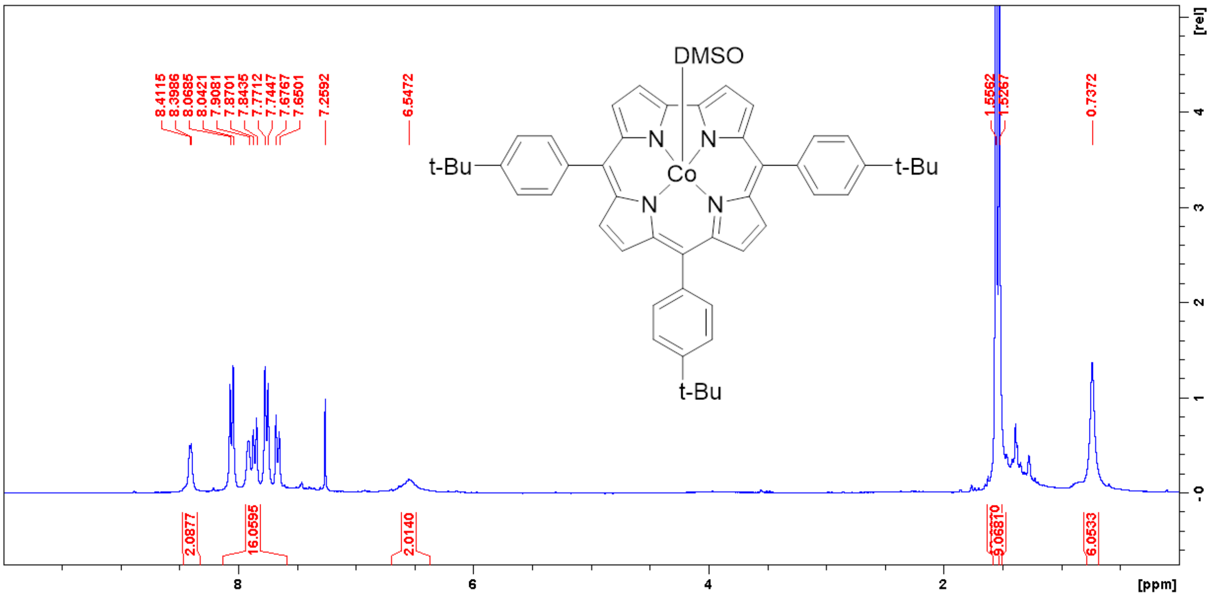

Figure S1: ^{1}H NMR Spectrum of Catalytically Active DMSO-Tagged Co-Corrole (**1**) in $CDCl_3$.

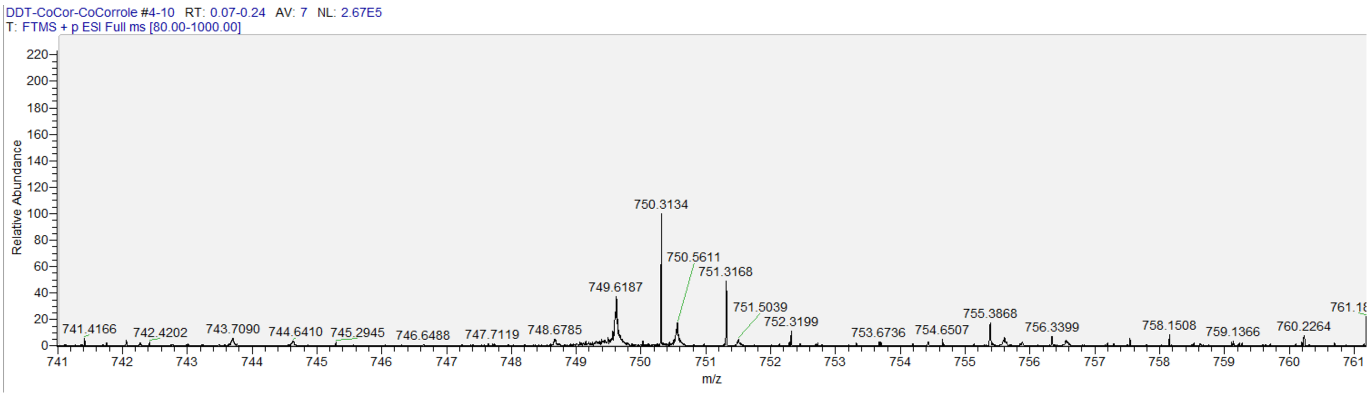

Figure S2: HR-MS of Catalytically Active DMSO-Tagged Co-Corrole (**1**).

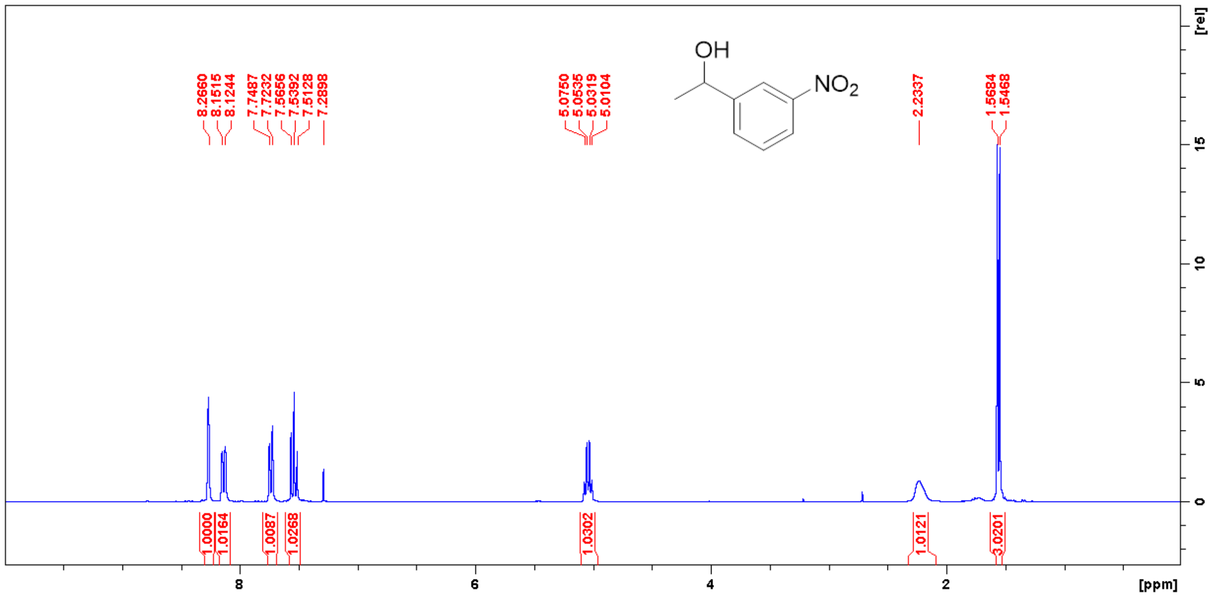

Figure S3: ^{1}H NMR Spectrum of 1-(3-Nitrophenyl)ethan-1-ol (**2p**) in $CDCl_3$.

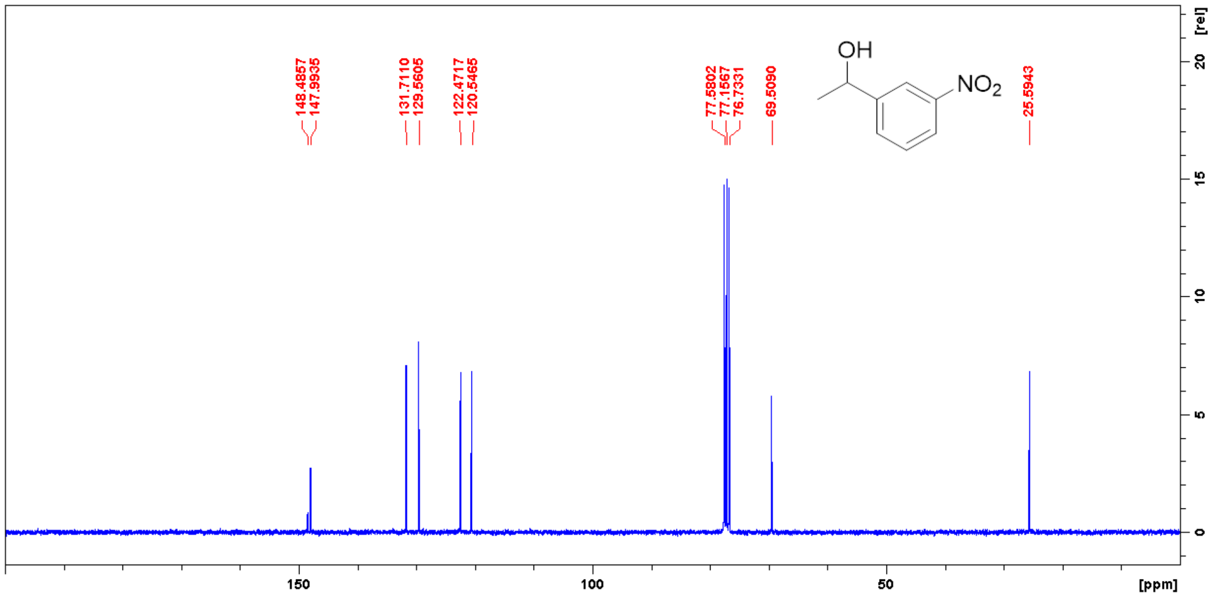

Figure S4: $^{13}C\{^{1}H\}$ NMR Spectrum of 1-(3-Nitrophenyl)ethan-1-ol (**2p**) in $CDCl_3$.

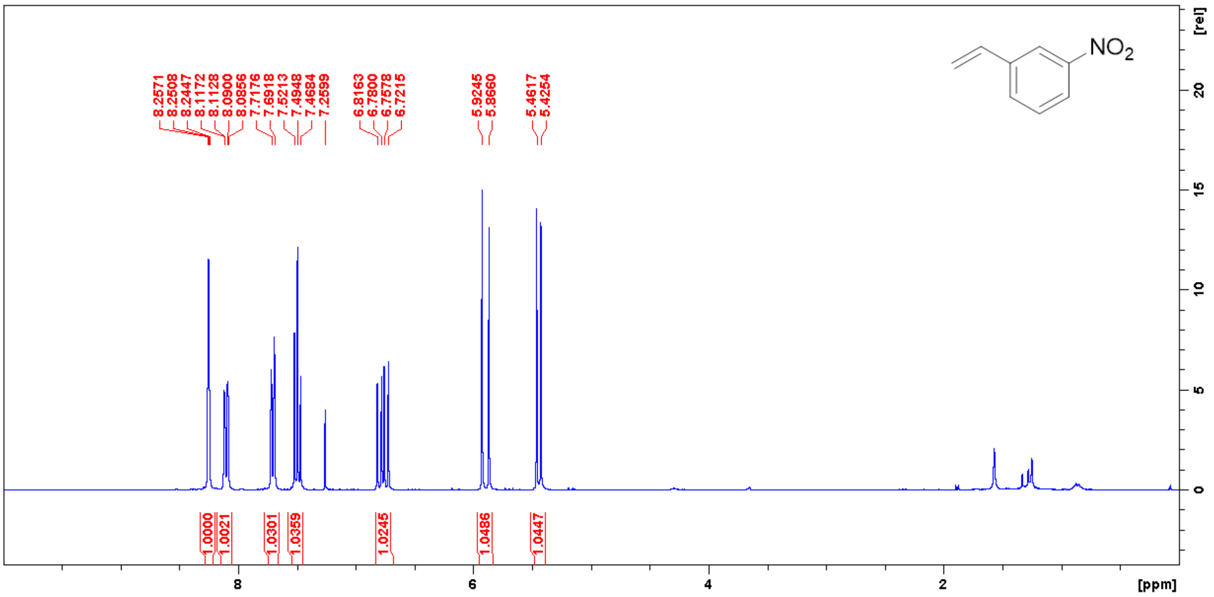

Figure S5: ^{1}H NMR Spectrum of 3-Nitrostyrene in $CDCl_3$.

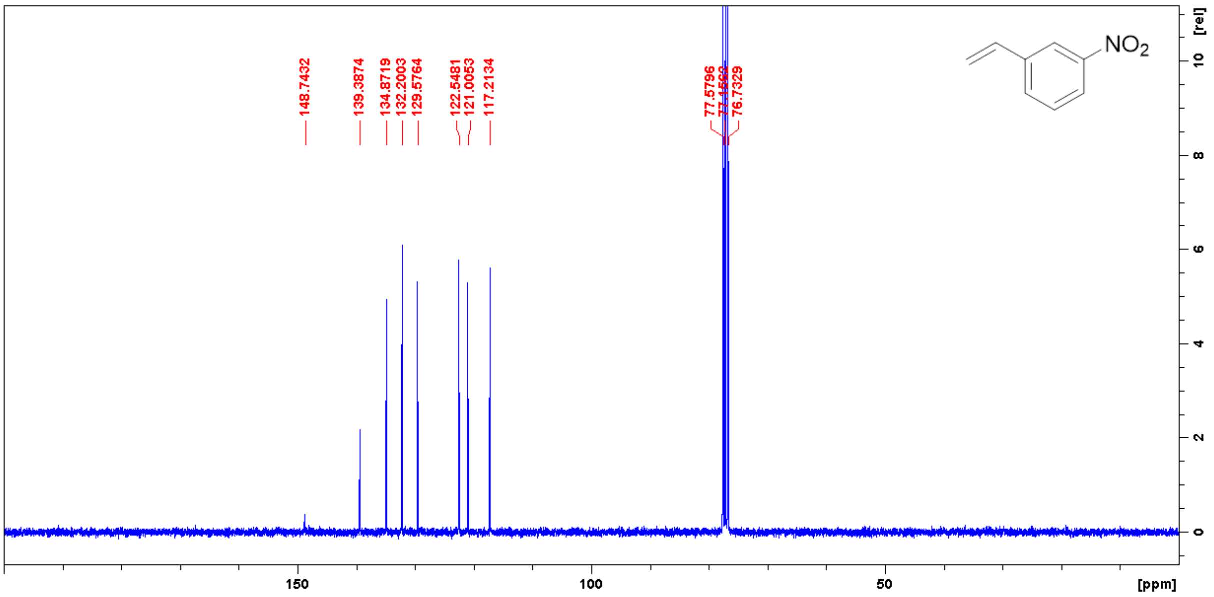

Figure S6: $^{13}C\{^{1}H\}$ NMR Spectrum of 3-Nitrostyrene in $CDCl_3$.

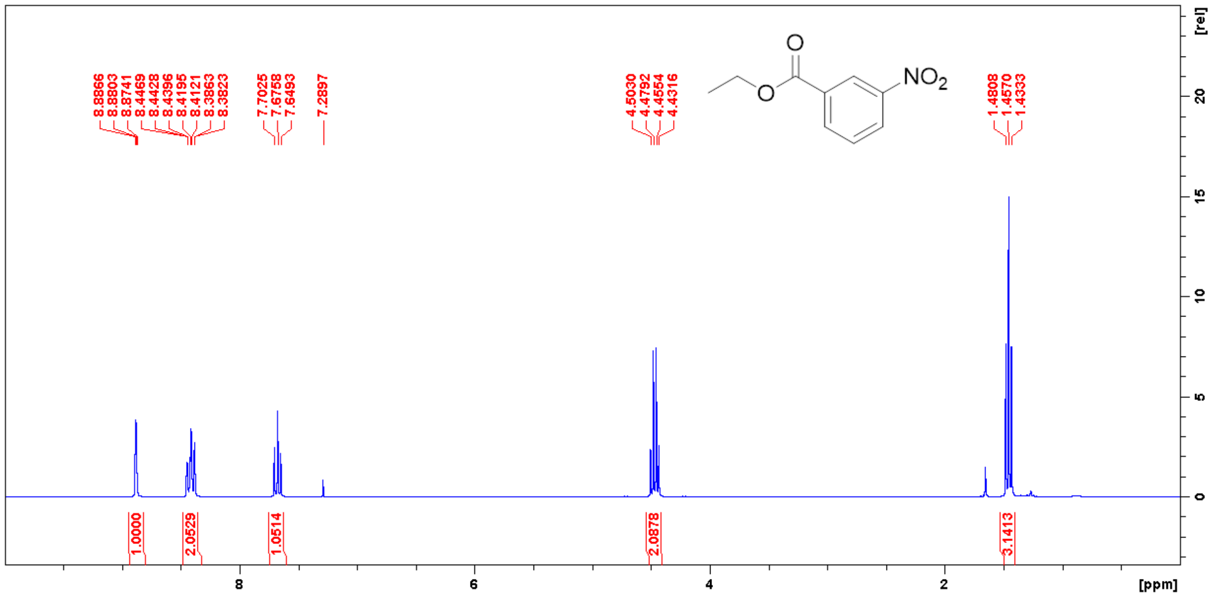

Figure S7: ^{1}H NMR Spectrum of Ethyl-3-nitrobenzoate (**2r**) in $CDCl_3$.

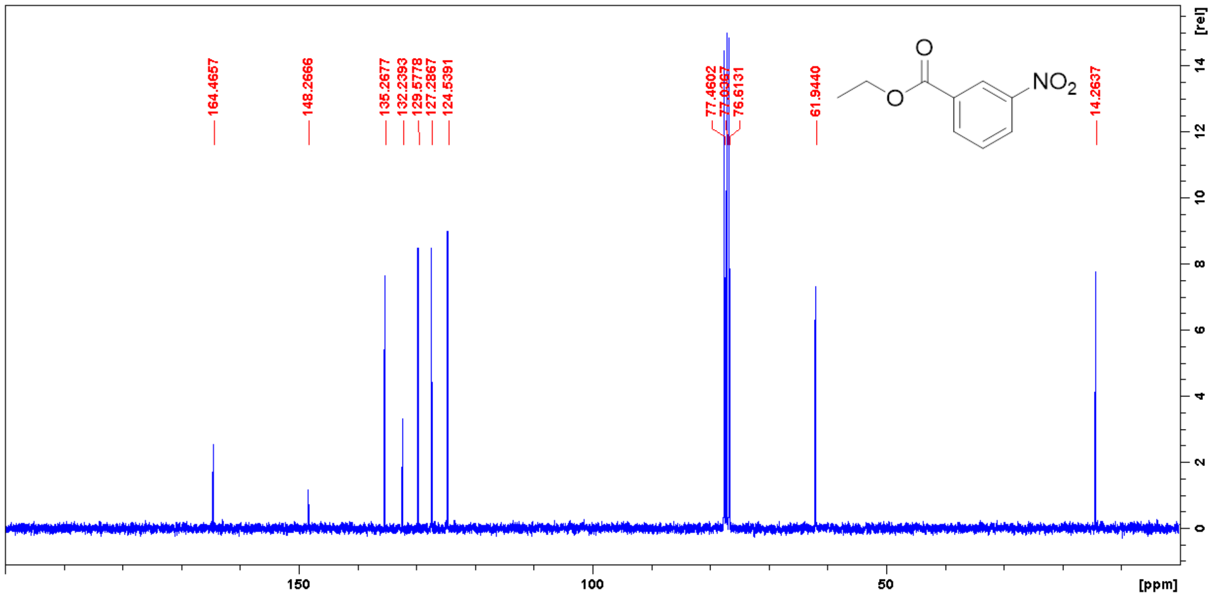

Figure S8: ^{13}C{^{1}H} NMR Spectrum of Ethyl-3-nitrobenzoate (**2r**) in $CDCl_3$.

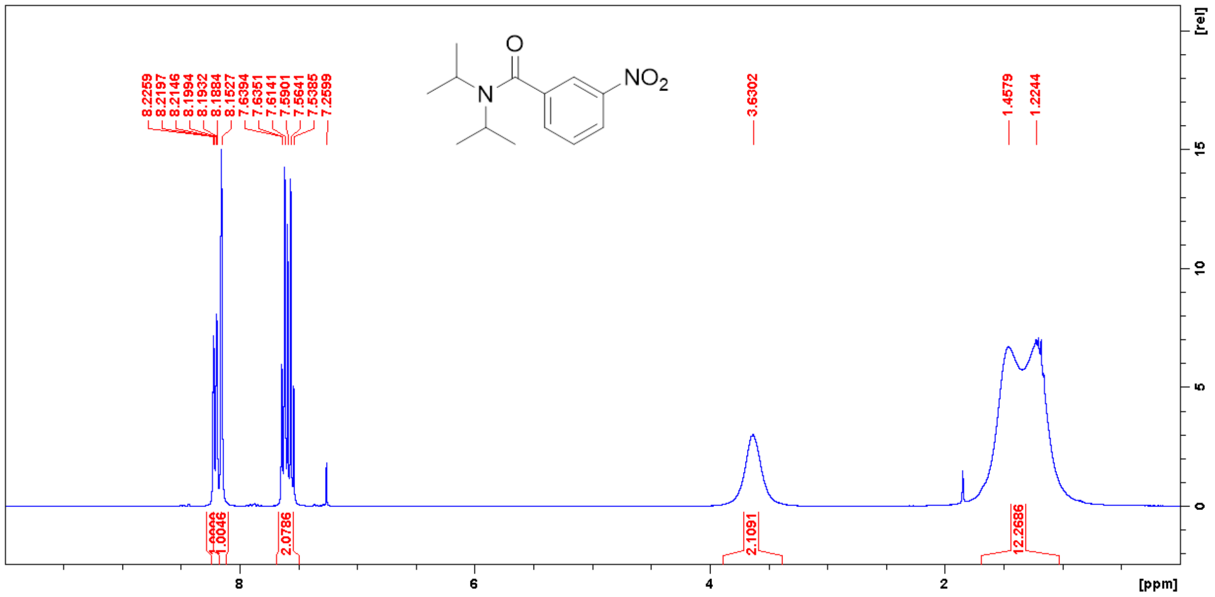

Figure S9: ^{1}H NMR Spectrum of *N*,*N*-Diisopropyl-3-nitrobenzamide (**2s**) in $CDCl_3$.

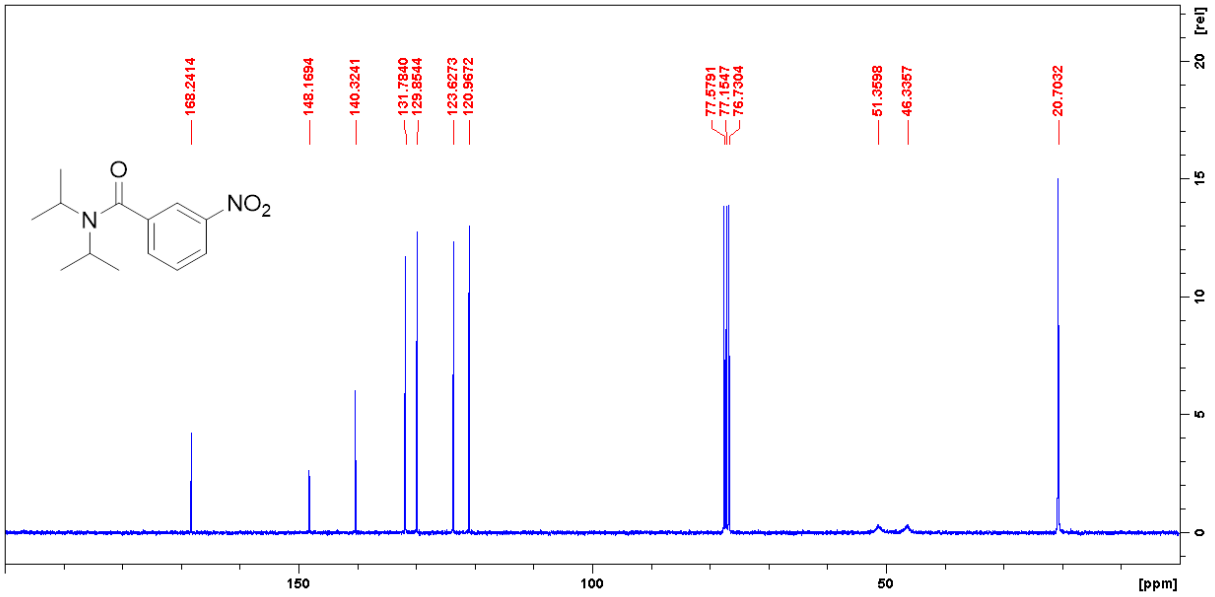

Figure S10: ^{13}C{^{1}H} NMR Spectrum of *N*,*N*-Diisopropyl-3-nitrobenzamide (**2s**) in $CDCl_3$.

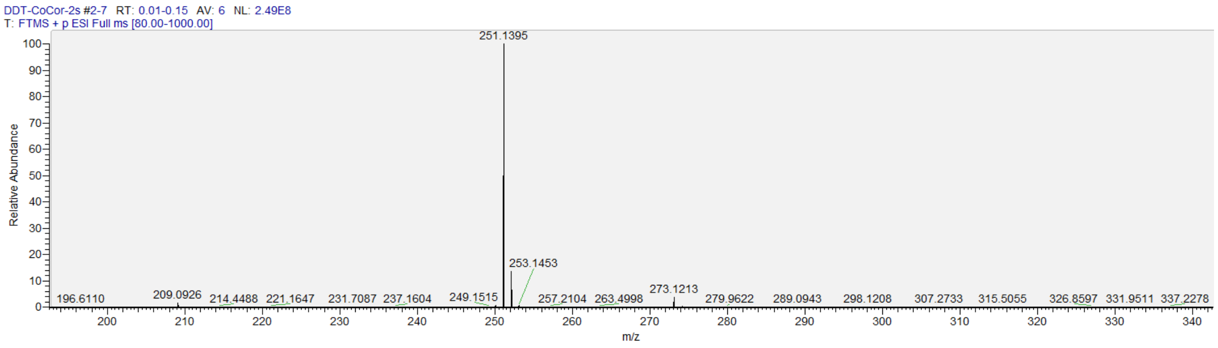

Figure S11: HR-MS of *N*,*N*-Diisopropyl-3-nitrobenzamide (**2s**).

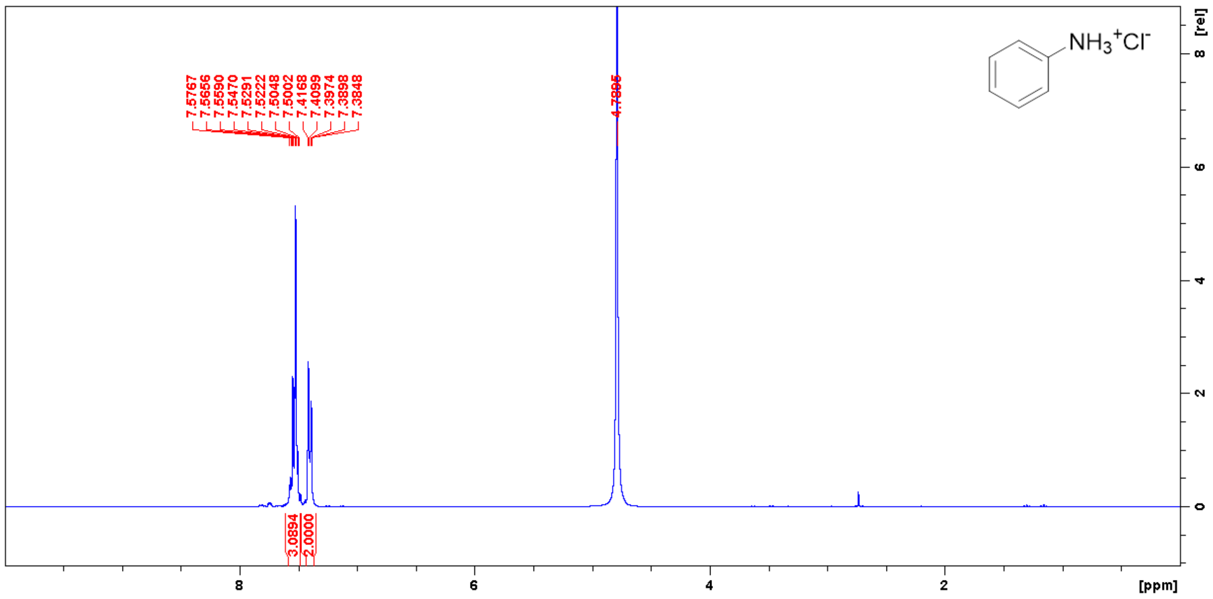

Figure S12: ^{1}H NMR Spectrum of Anilinium Hydrochloride (**4a**) in D_2O.

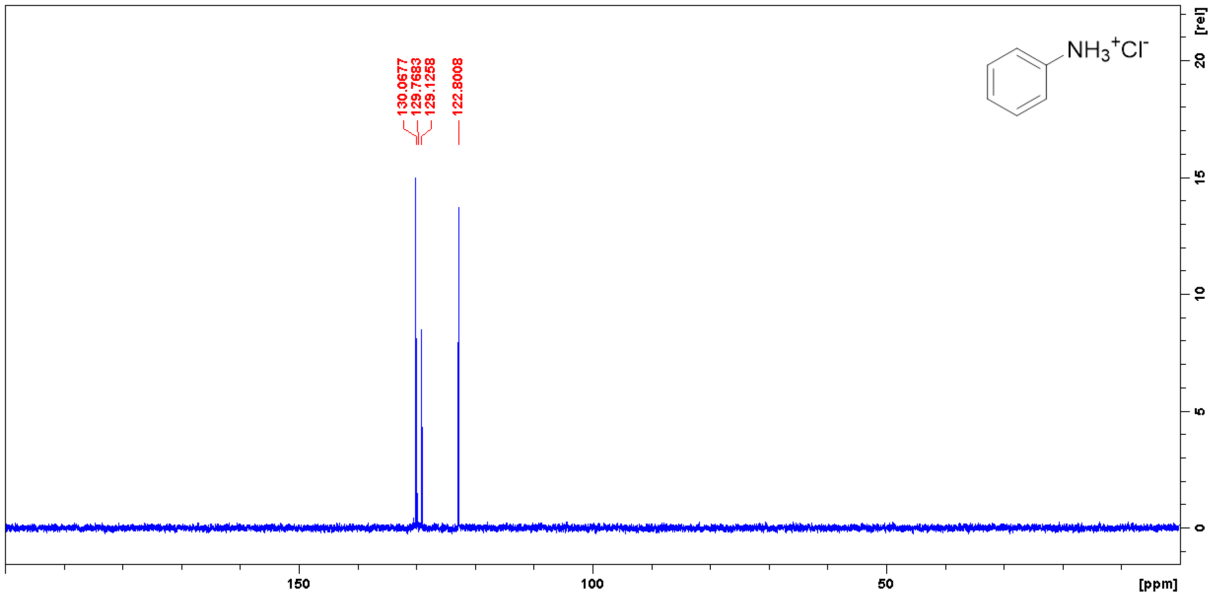

Figure S13: $^{13}C\{^{1}H\}$ NMR Spectrum of Anilinium Hydrochloride (**4a**) in D_2O.

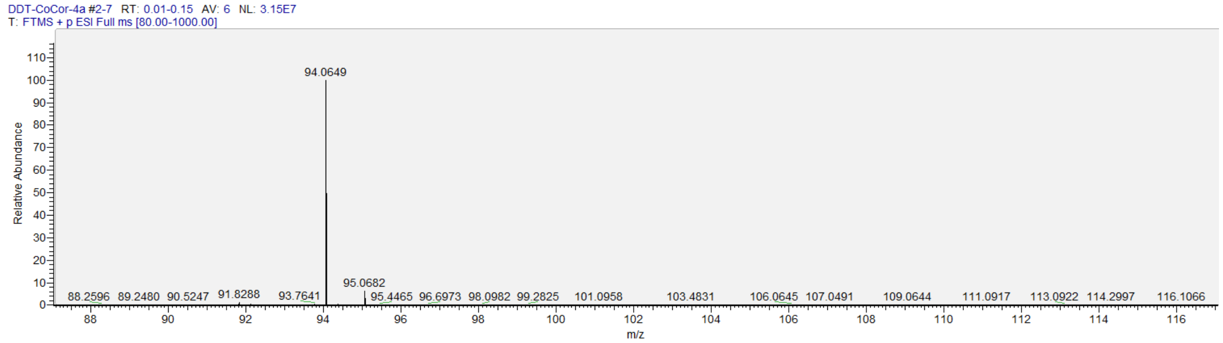

Figure S14: HR-MS of Anilinium Hydrochloride (**4a**).

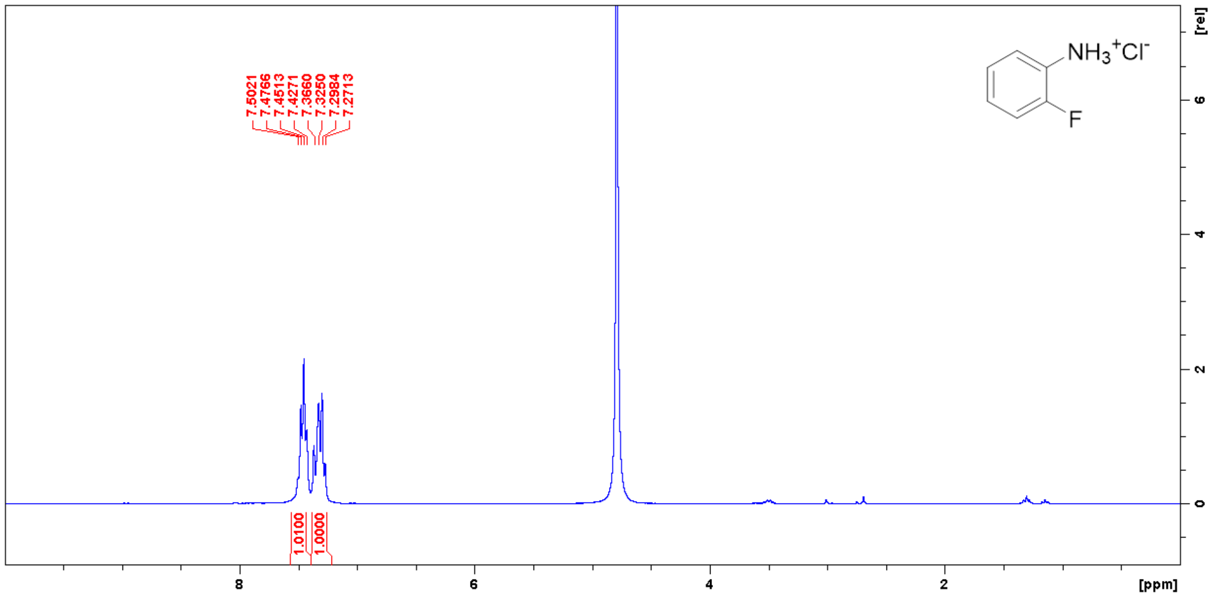

Figure S15: ^{1}H NMR Spectrum of 2-Fluoroanilinium Hydrochloride (**4b**) in D_2O.

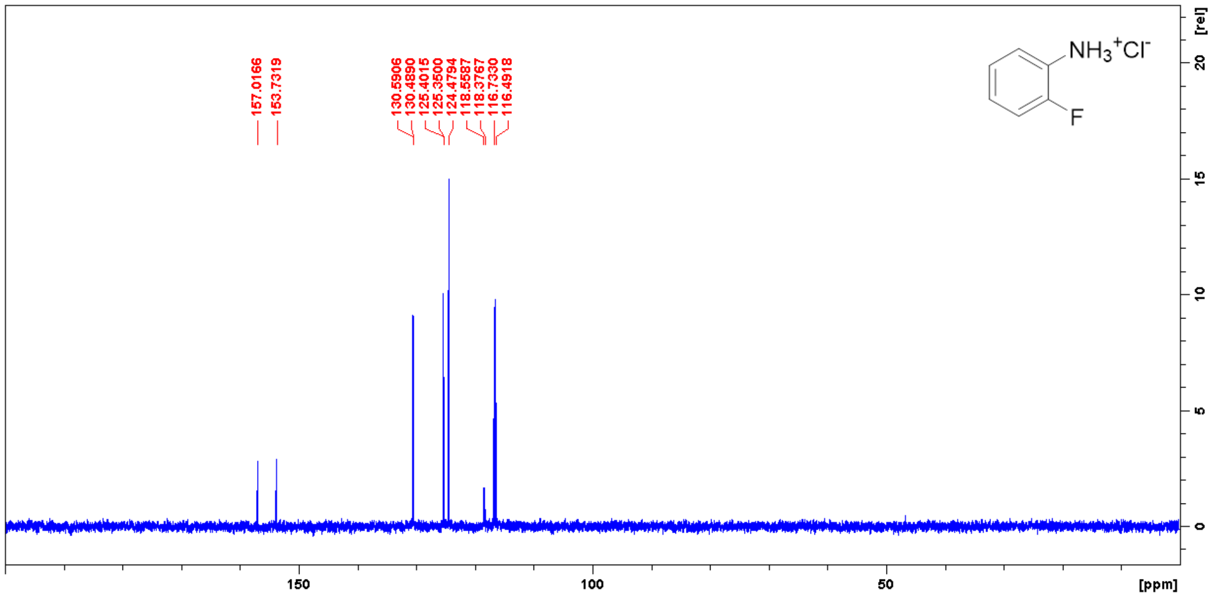

Figure S16: $^{13}C\{^{1}H\}$ NMR Spectrum of 2-Fluoroanilinium Hydrochloride (**4b**) in D_2O.

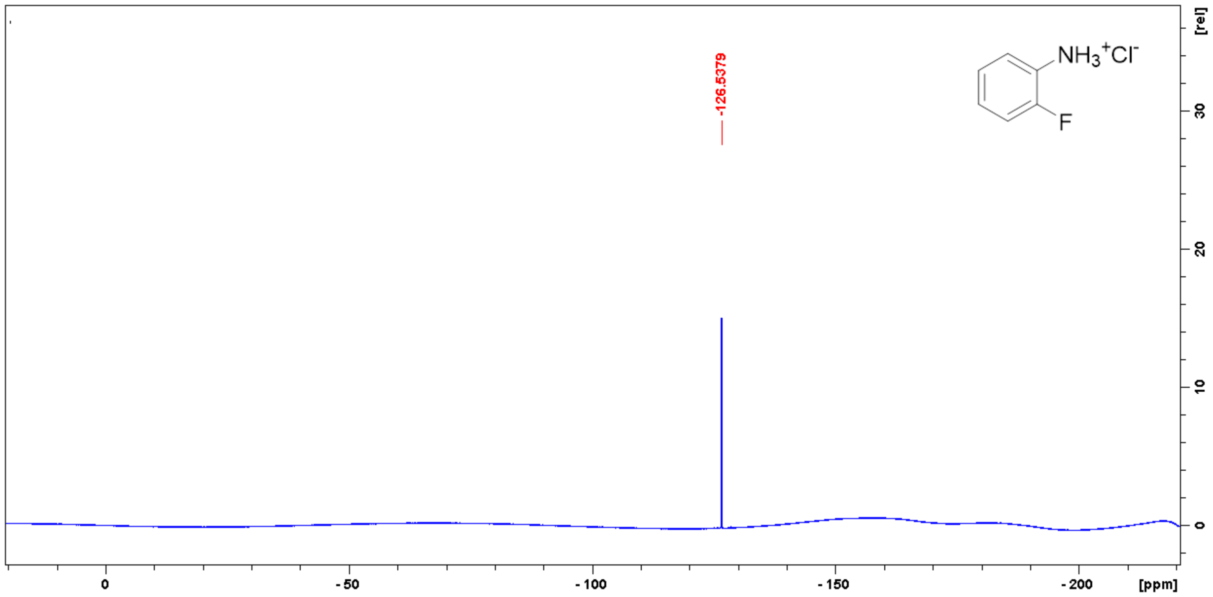

Figure S17: ^{19}F NMR Spectrum of 2-Fluoroanilinium Hydrochloride (**4b**) in D_2O.

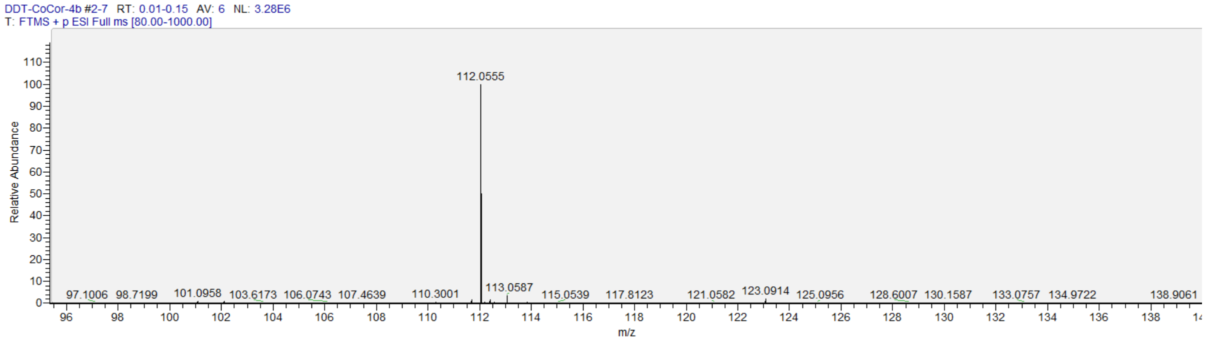

Figure S18: HR-MS of 2-Fluoroanilinium Hydrochloride (**4b**).

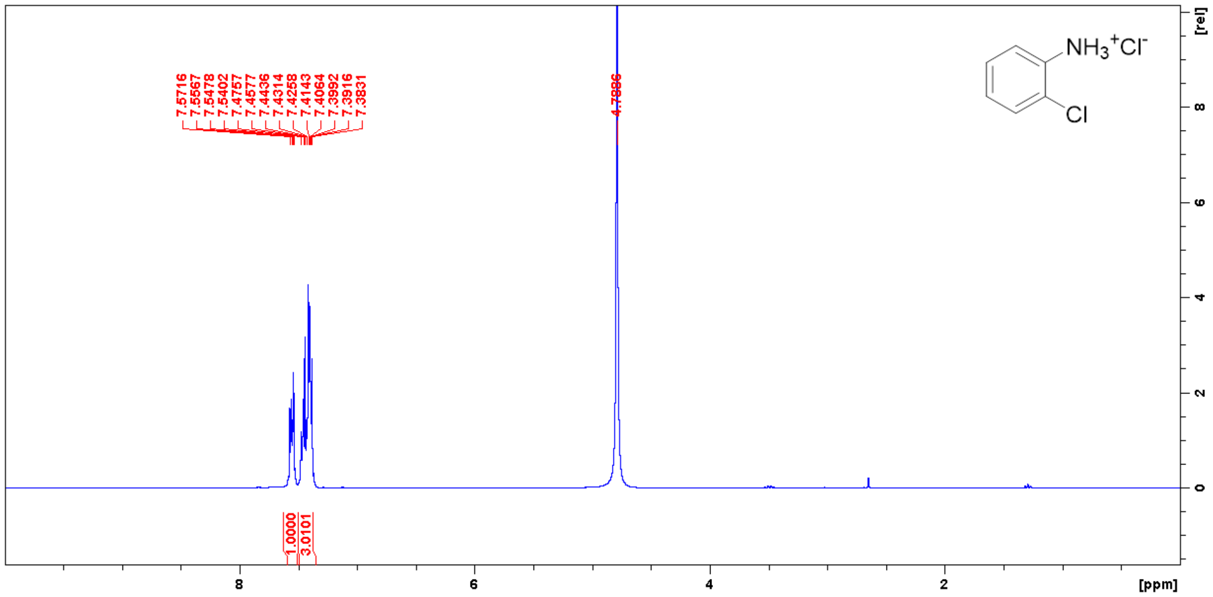

Figure S19: ^{1}H NMR Spectrum of 2-Chloroanilinium Hydrochloride (**4c**) in D_2O.

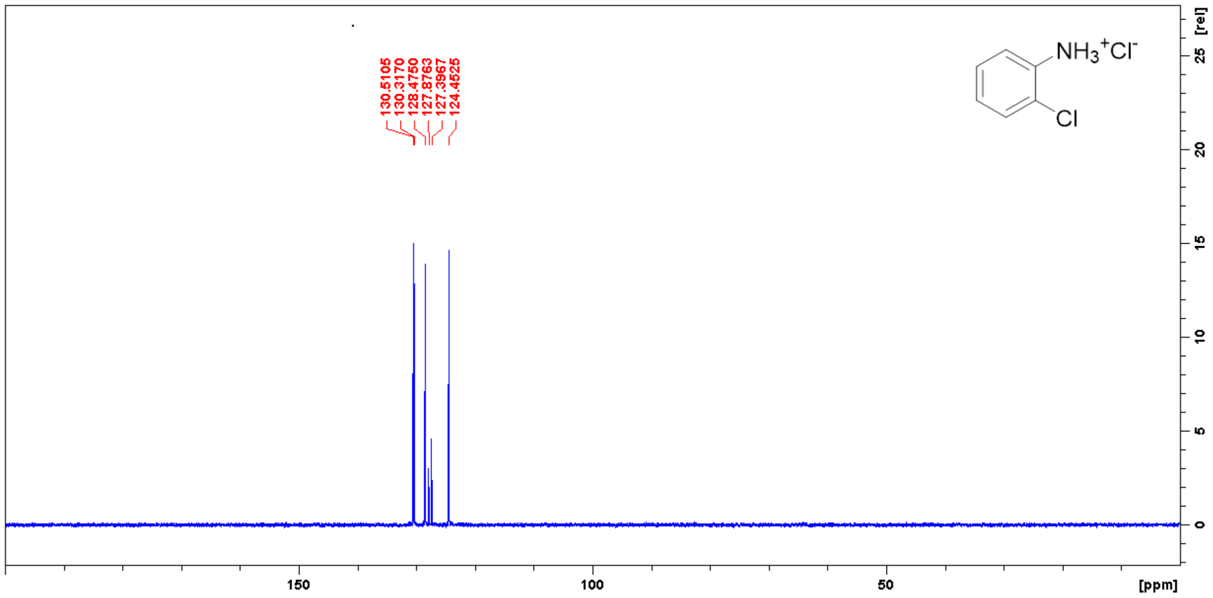

Figure S20: $^{13}C\{^{1}H\}$ NMR Spectrum of 2-Chloroanilinium Hydrochloride (**4c**) in D_2O.

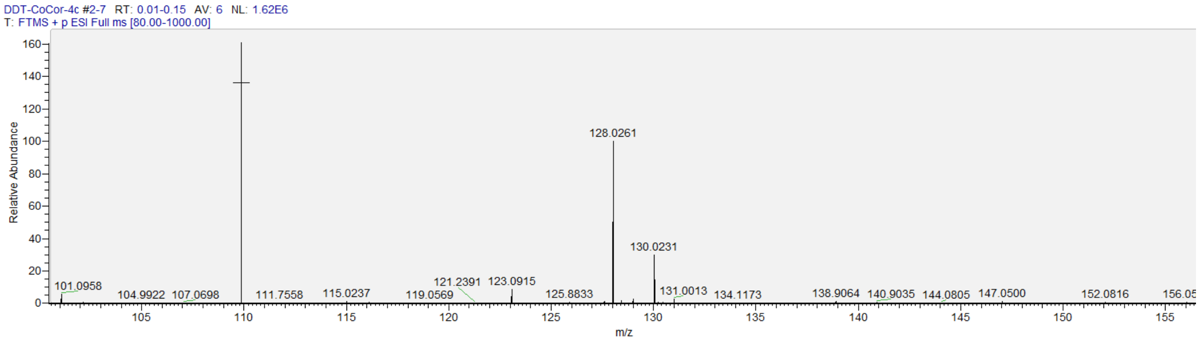

Figure S21: HR-MS of 2-Chloroanilinium Hydrochloride (**4c**).

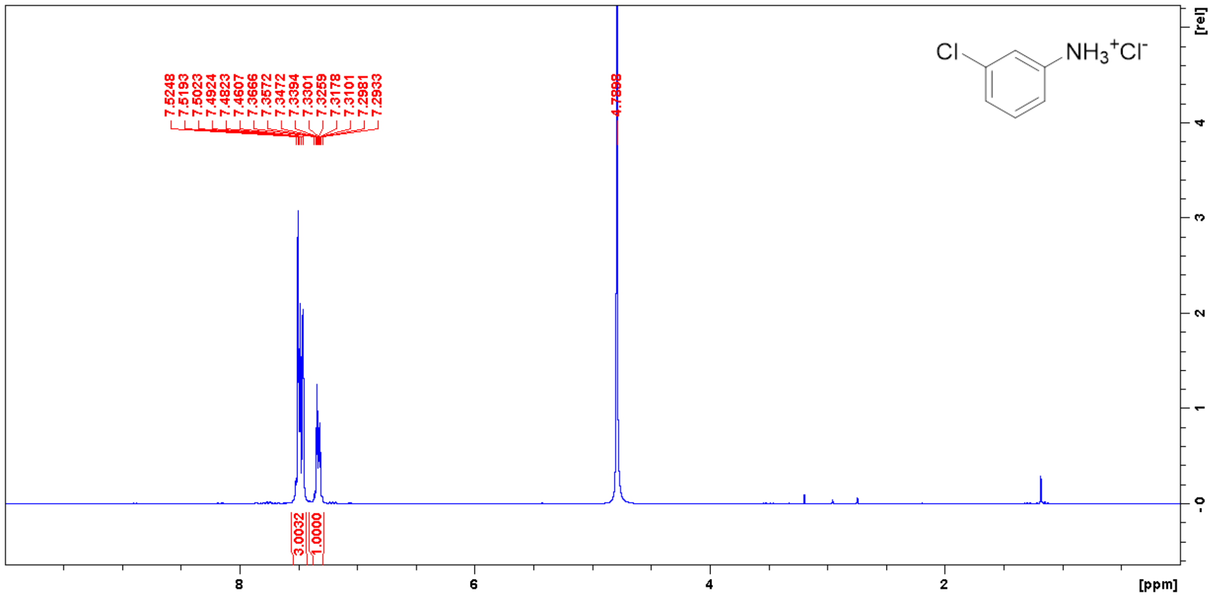

Figure S22: ^{1}H NMR spectrum of 3-chloroanilinium hydrochloride (**4d**) in D_2O.

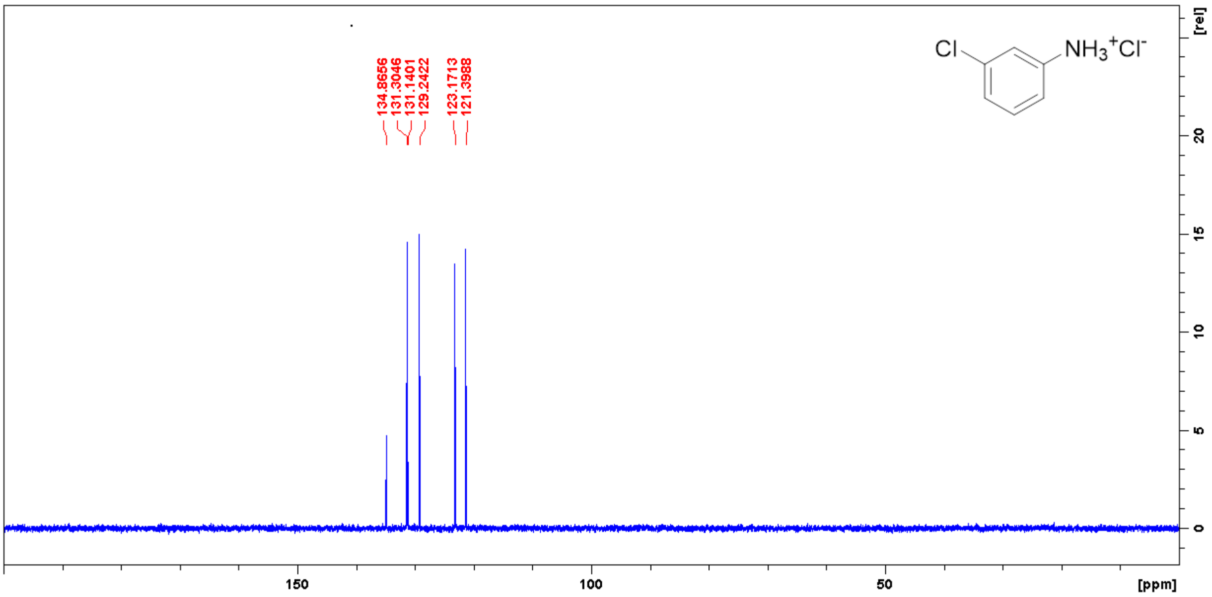

Figure S23: $^{13}C\{^{1}H\}$ NMR Spectrum of 3-Chloroanilinium Hydrochloride (**4d**) in D_2O.

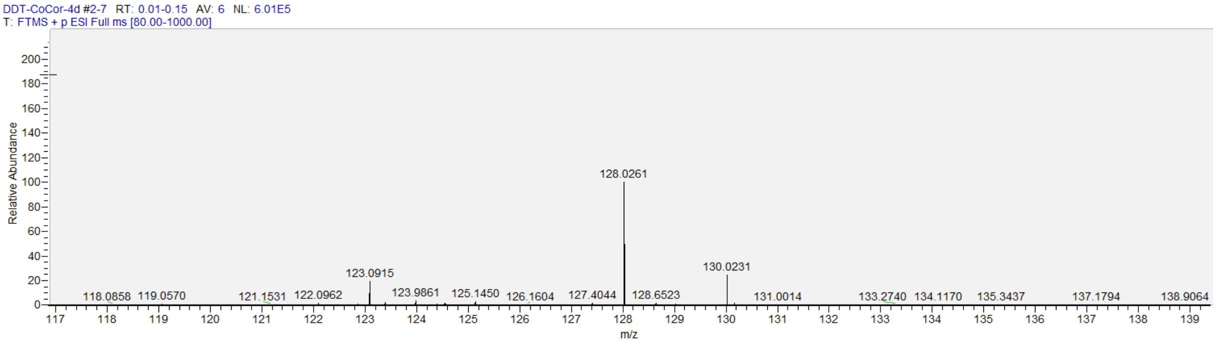

Figure S24: HR-MS of 3-Chloroanilinium Hydrochloride (**4d**).

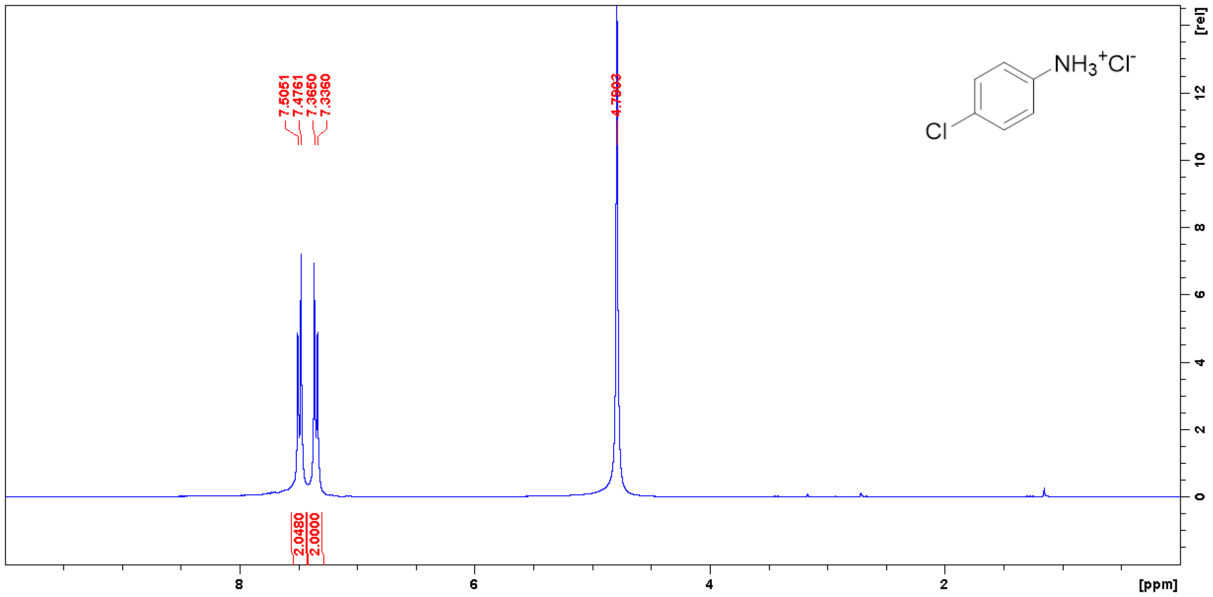

Figure S25: ^{1}H NMR Spectrum of 4-Chloroanilinium Hydrochloride (**4e**) in D_2O.

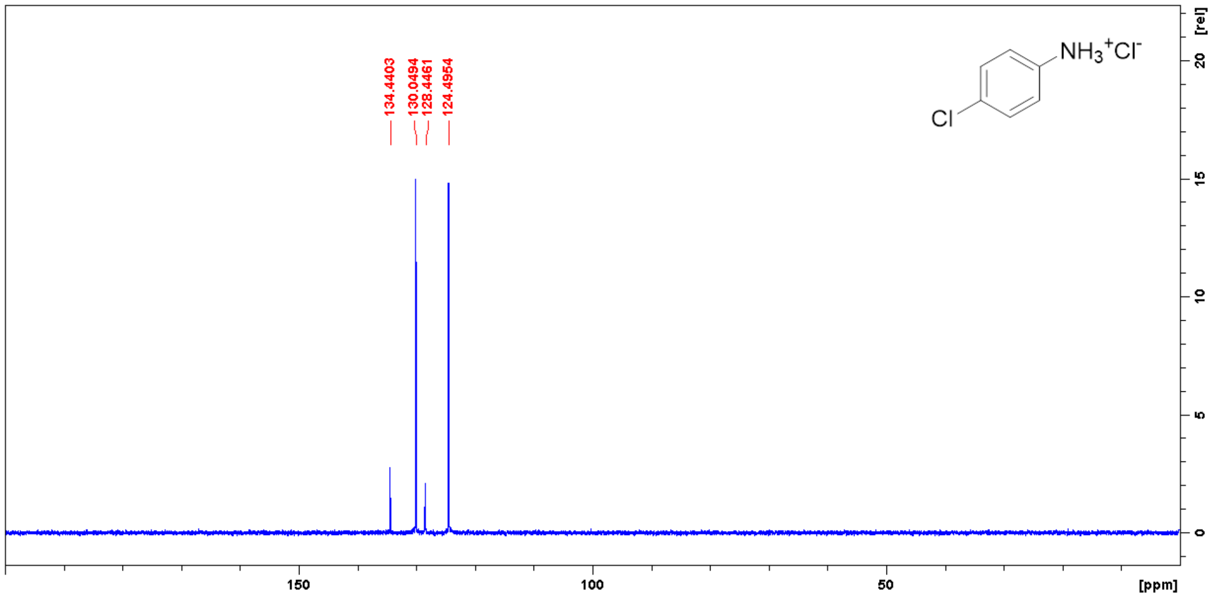

Figure S26: $^{13}C\{^{1}H\}$ NMR Spectrum of 4-Chloroanilinium Hydrochloride (**4e**) in D_2O.

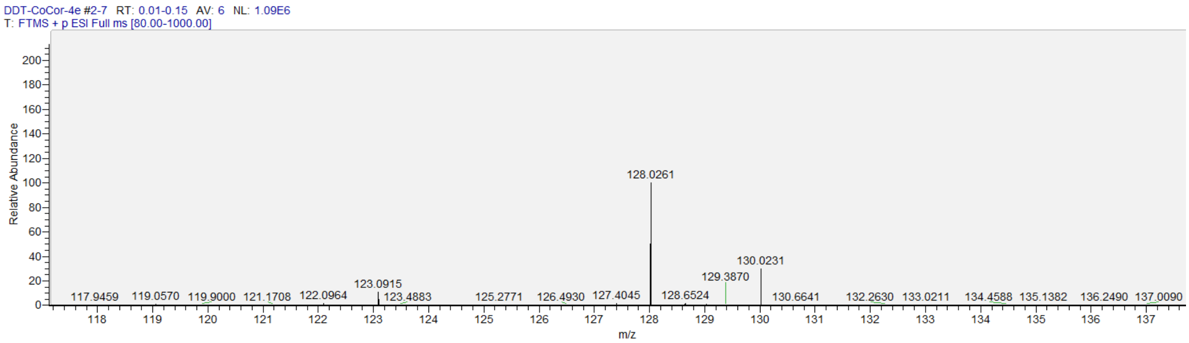

Figure S27: HR-MS of 4-Chloroanilinium Hydrochloride (**4e**).

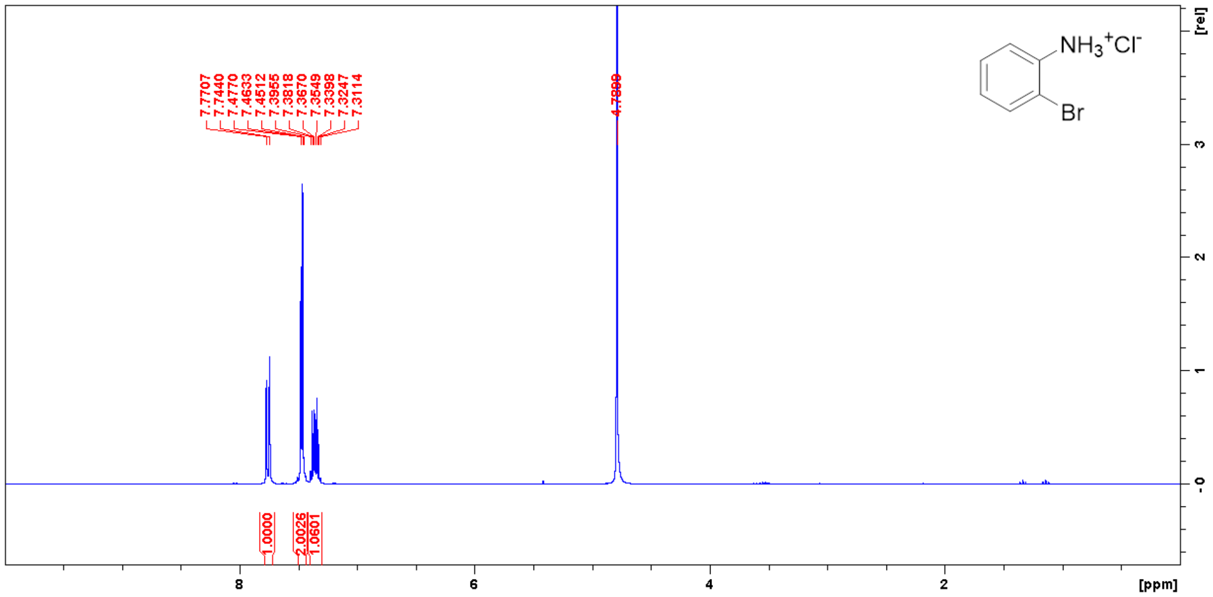

Figure S28: ^{1}H NMR Spectrum of 2-Bromoanilinium Hydrochloride (**4f**) in D_2O.

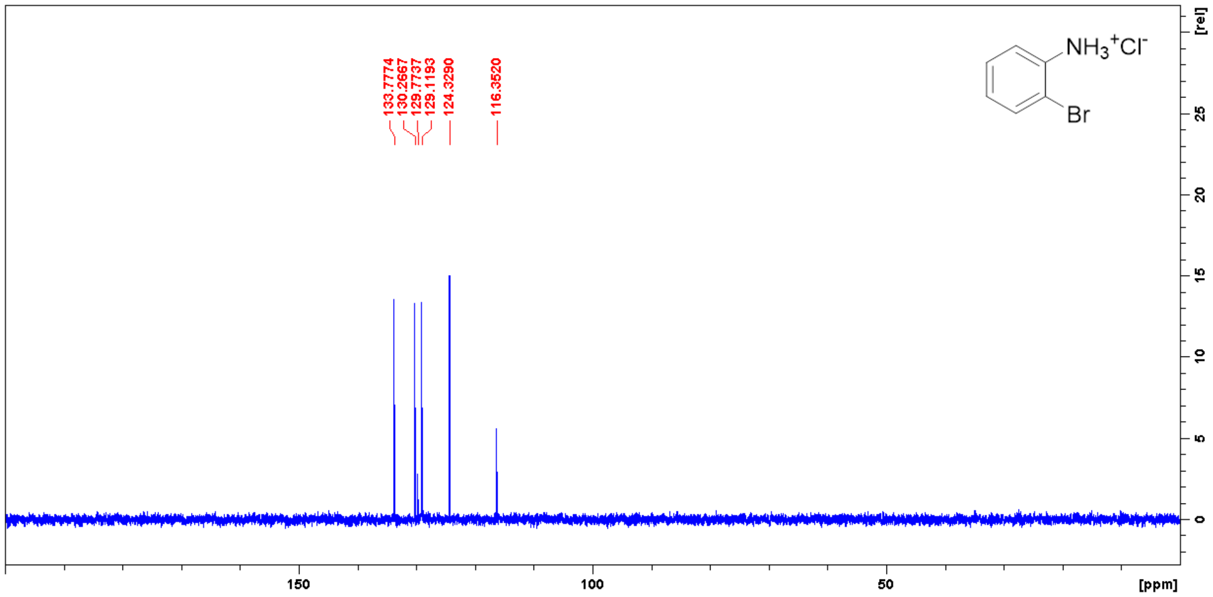

Figure S29: $^{13}C\{^{1}H\}$ NMR Spectrum of 2-Bromoanilinium Hydrochloride (**4f**) in D_2O.

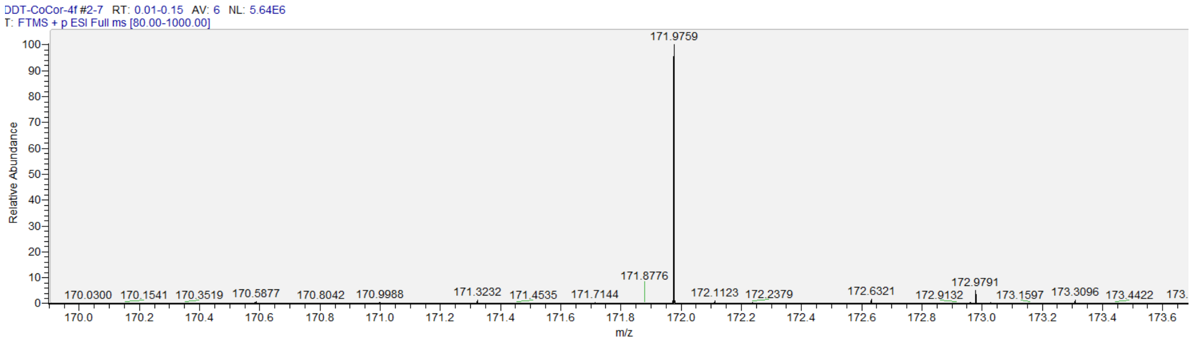

Figure S30: HR-MS of 2-Bromoanilinium Hydrochloride (**4f**).

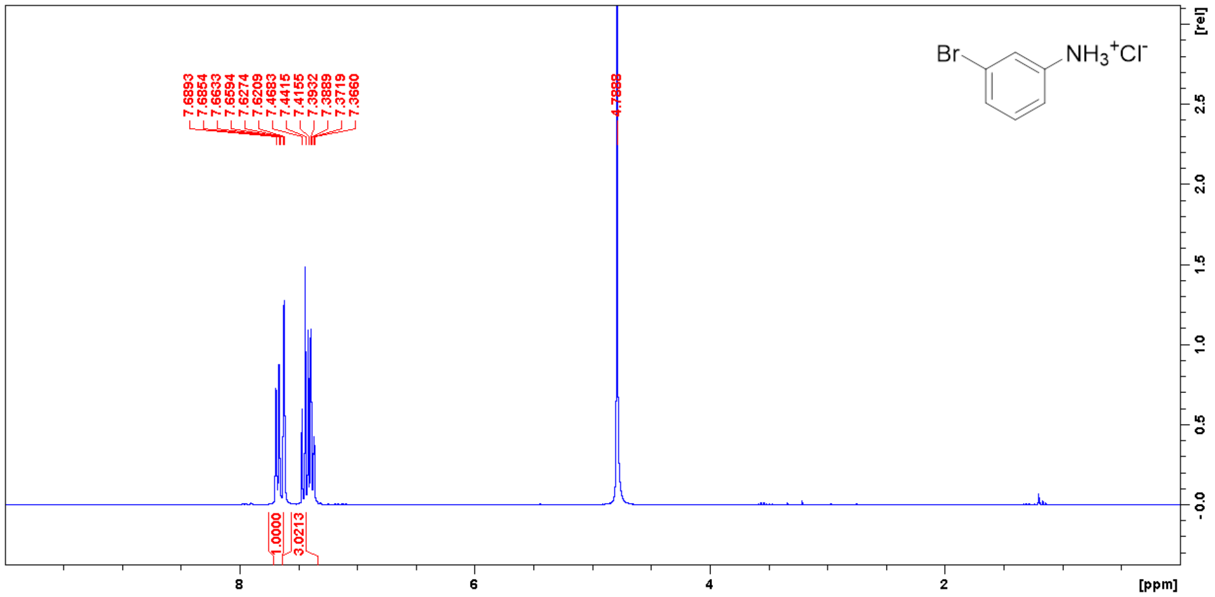

Figure S31: ^{1}H NMR Spectrum of 3-Bromoanilinium Hydrochloride (**4g**) in D_2O.

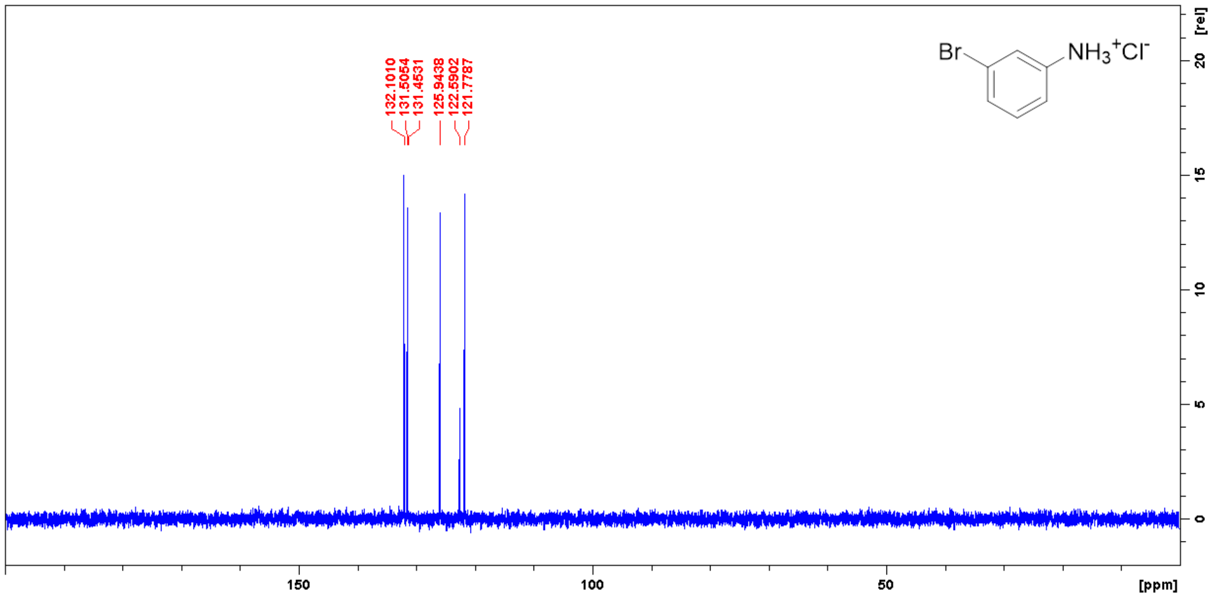

Figure S32: $^{13}C\{^{1}H\}$ NMR Spectrum of 3-Bromoanilinium Hydrochloride (**4g**) in D_2O.

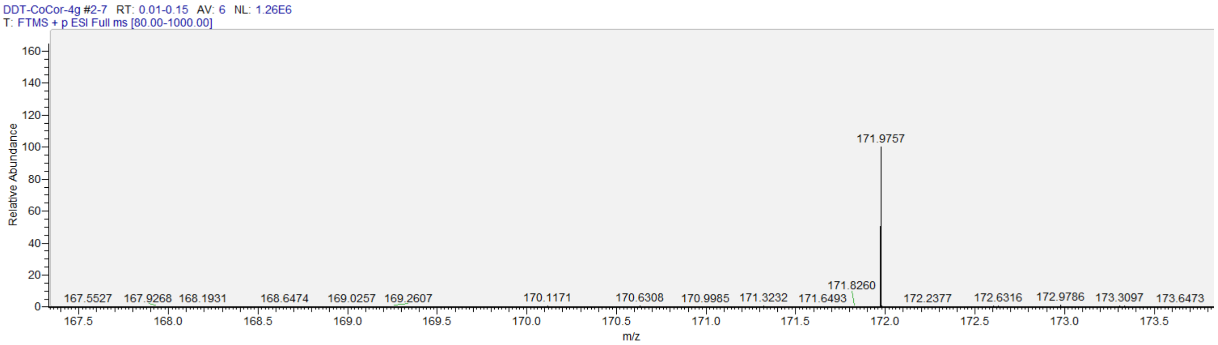

Figure S33: HR-MS of 3-Bromoanilinium Hydrochloride (**4g**).

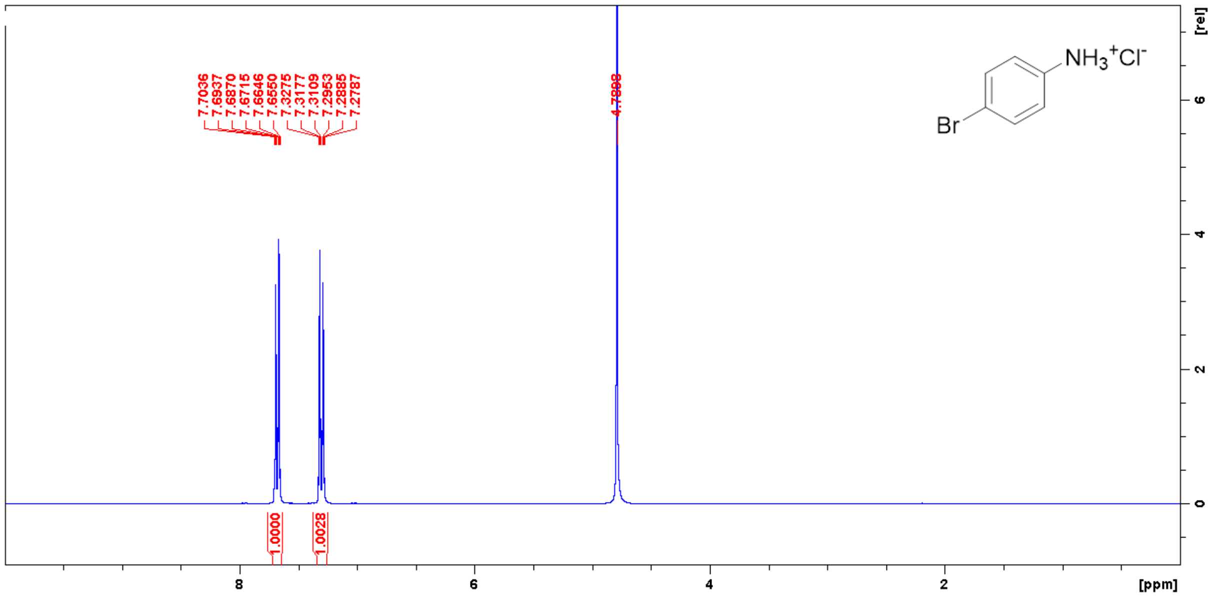

Figure S34: ^{1}H NMR Spectrum of 4-Bromoanilinium Hydrochloride (**4h**) in D_2O.

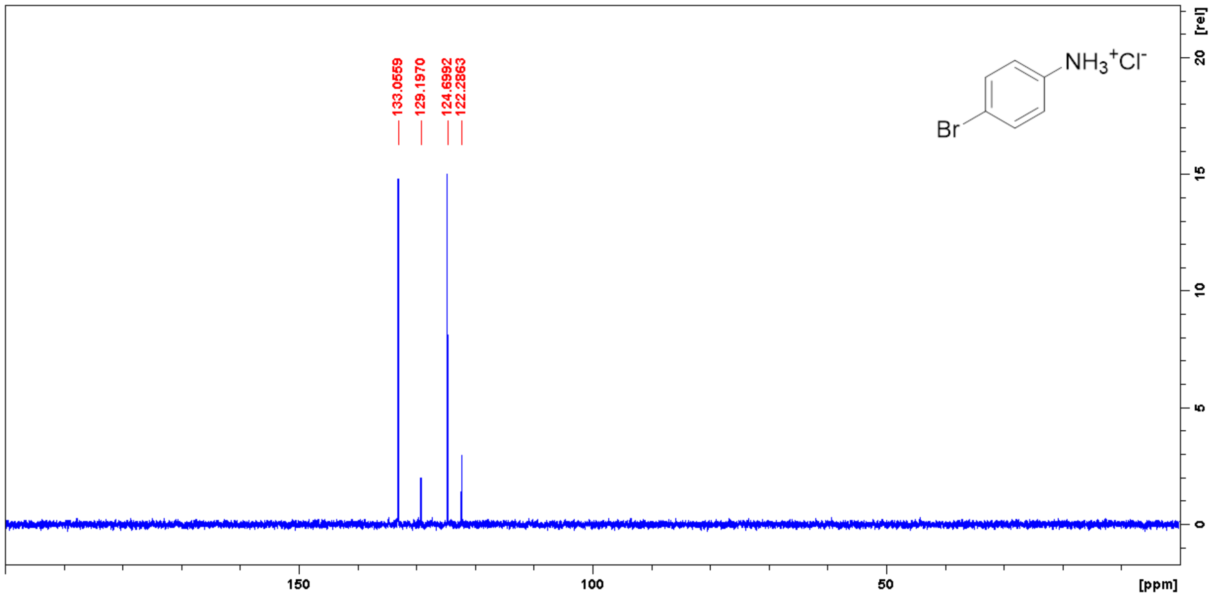

Figure S35: $^{13}C\{^1H\}$ NMR Spectrum of 4-Bromoanilinium Hydrochloride (**4h**) in D_2O.

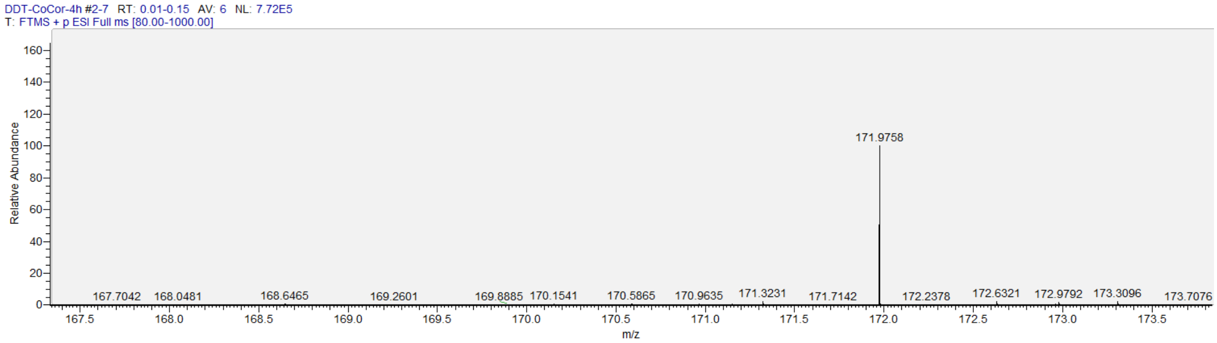

Figure S36: HR-MS of 4-Bromoanilinium Hydrochloride (**4h**).

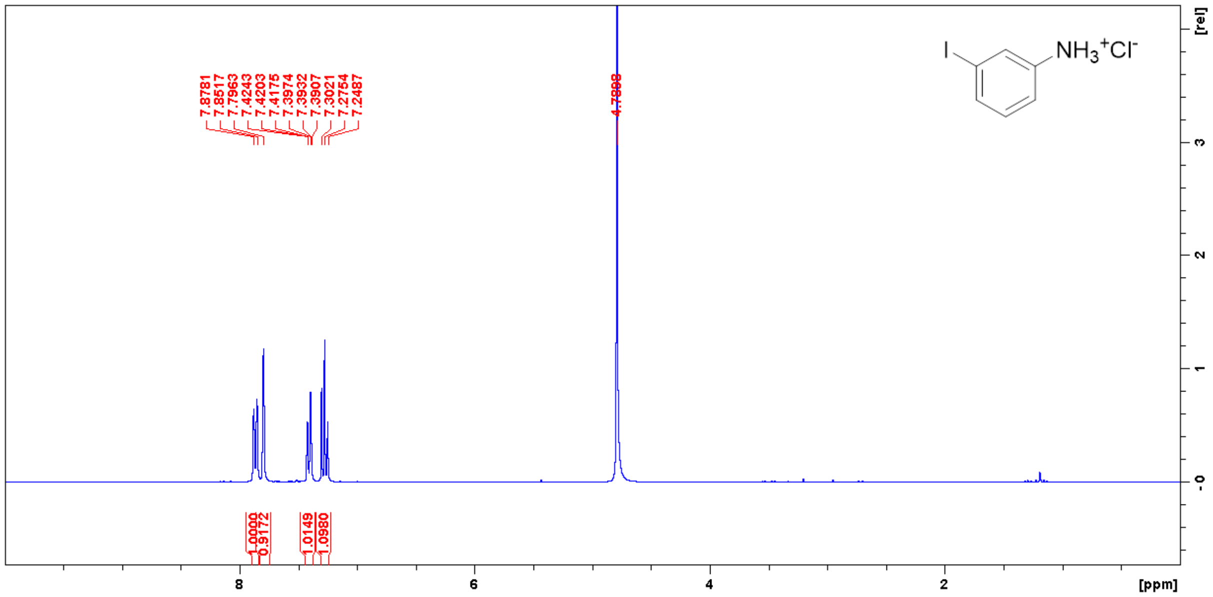

Figure S37: ^{1}H NMR Spectrum of 3-Iodoanilinium Hydrochloride (**4i**) in D_2O.

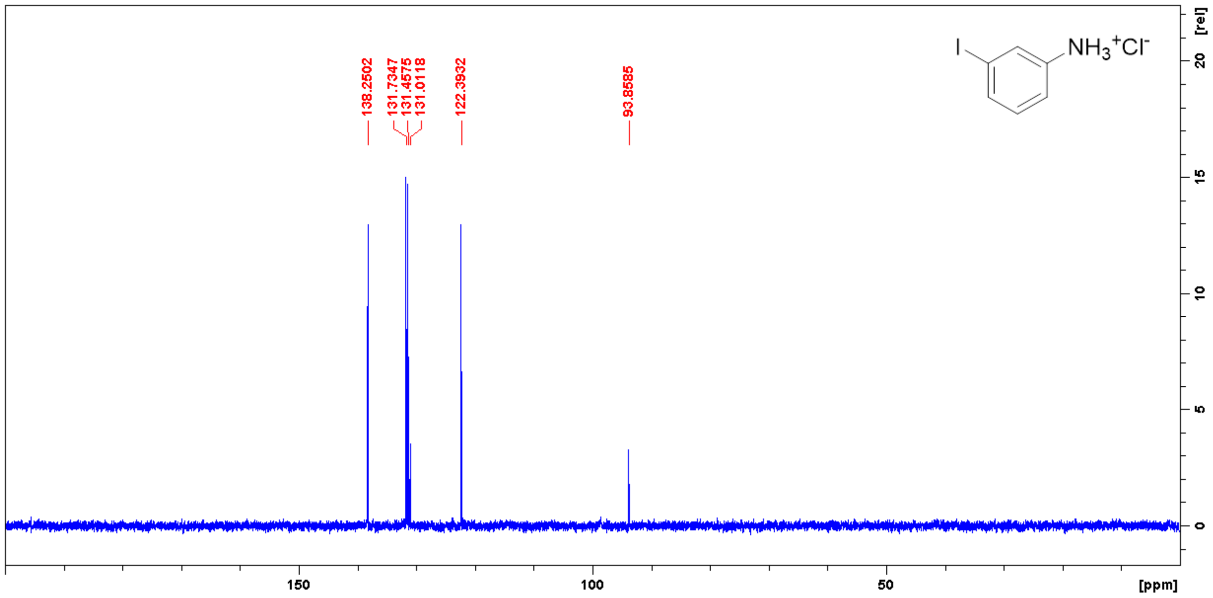

Figure S38: $^{13}C\{^{1}H\}$ NMR Spectrum of 3-Iodoanilinium Hydrochloride (**4i**) in D_2O.

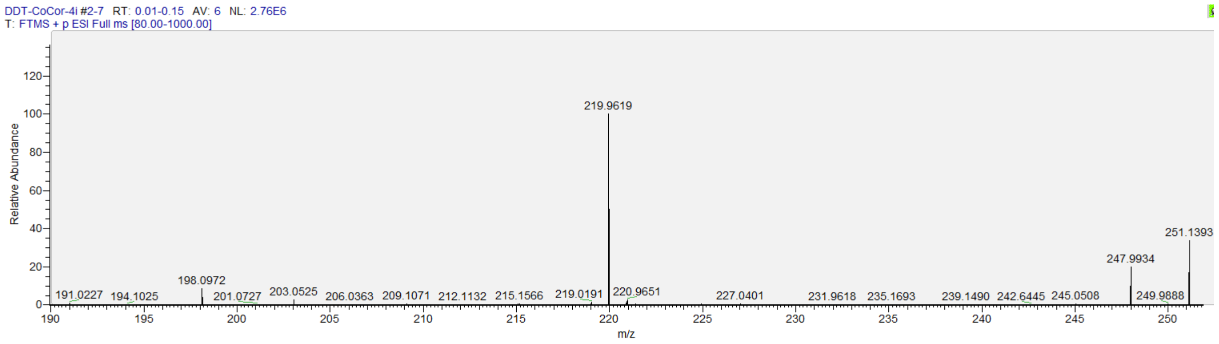

Figure S39: HR-MS of 3-Iodoanilinium Hydrochloride (**4i**).

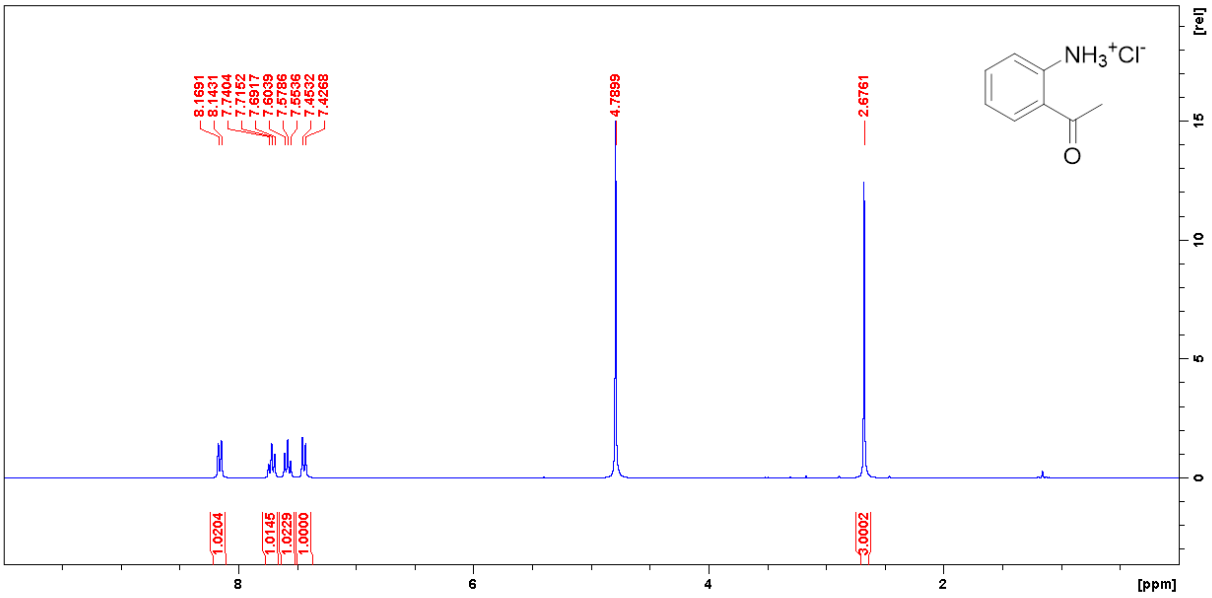

Figure S40: ^{1}H NMR Spectrum of 2-Acetylbenzenaminium Hydrochloride (**4j**) in D_2O.

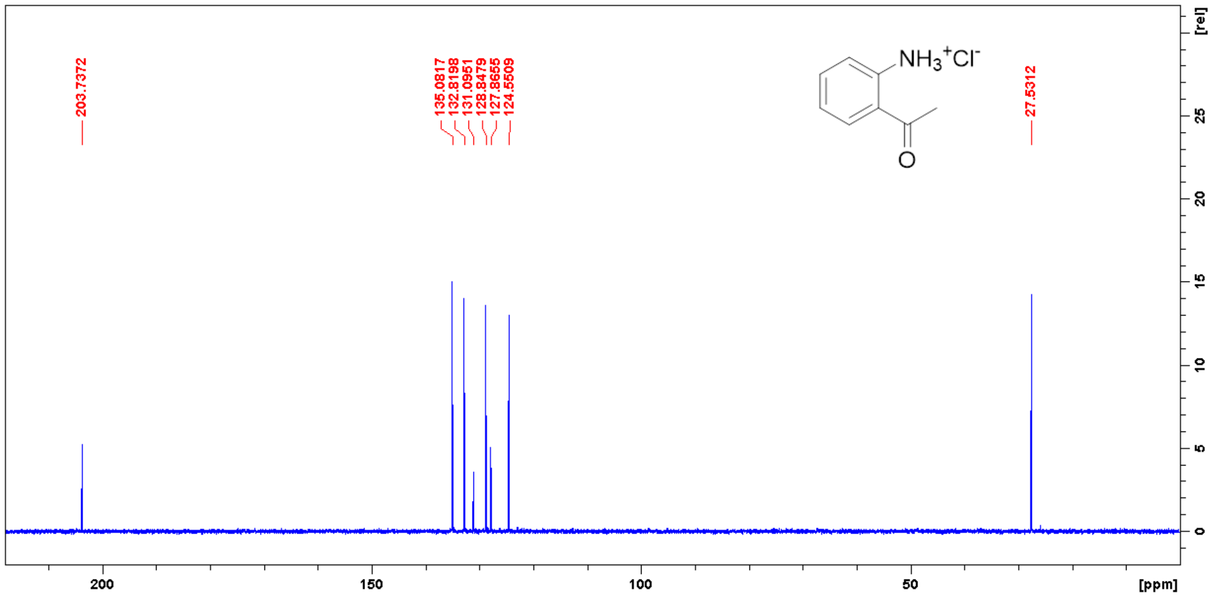

Figure S41: $^{13}C\{^1H\}$ NMR Spectrum of 2-Acetylbenzenaminium Hydrochloride (**4j**) in D_2O.

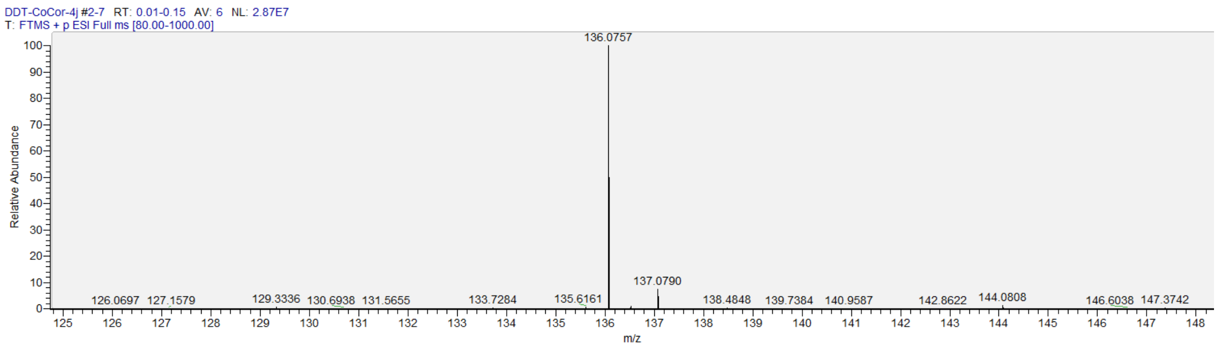

Figure S42: HR-MS of 2-Acetylbenzenaminium Hydrochloride (**4j**).

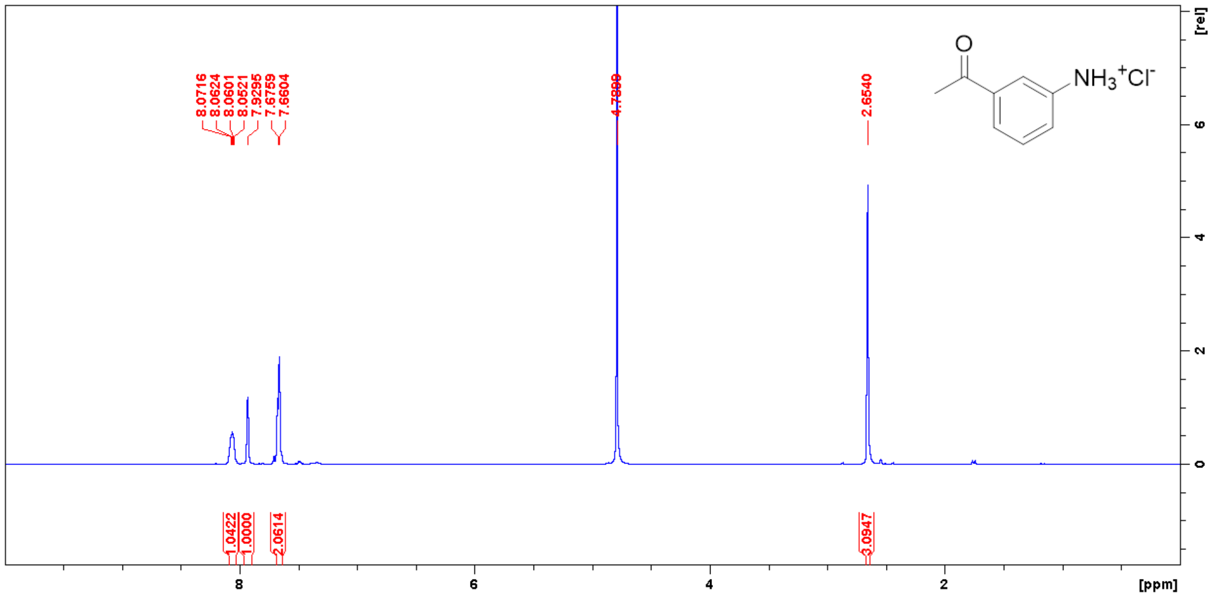

Figure S43: ^{1}H NMR Spectrum of 3-Acetylbenzenaminium Hydrochloride (**4k**) in D_2O.

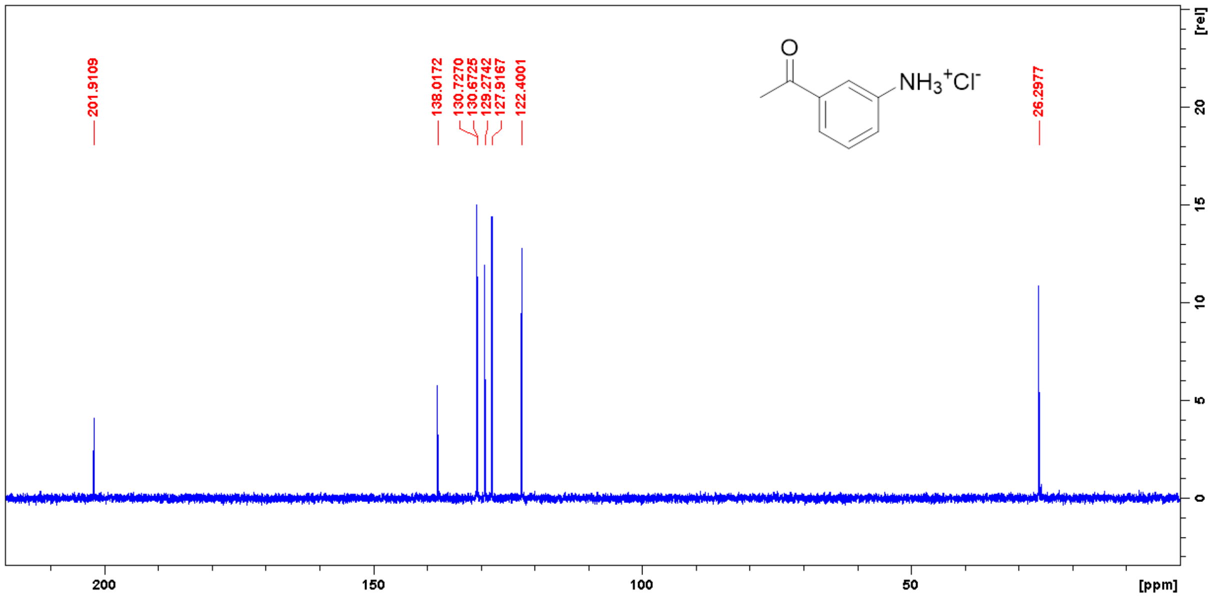

Figure S44: $^{13}C\{^{1}H\}$ NMR Spectrum of 3-Acetylbenzenaminium Hydrochloride (**4k**) in D_2O.

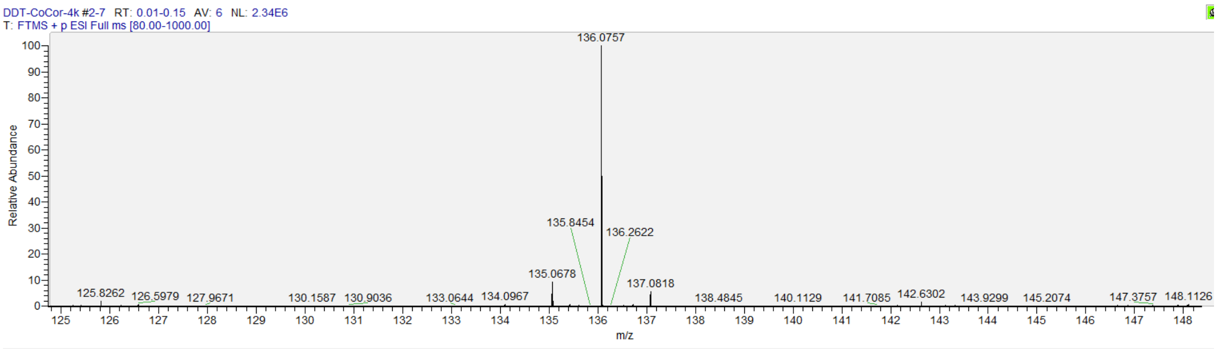

Figure S45: HR-MS of 3-Acetylbenzenaminium Hydrochloride (**4k**).

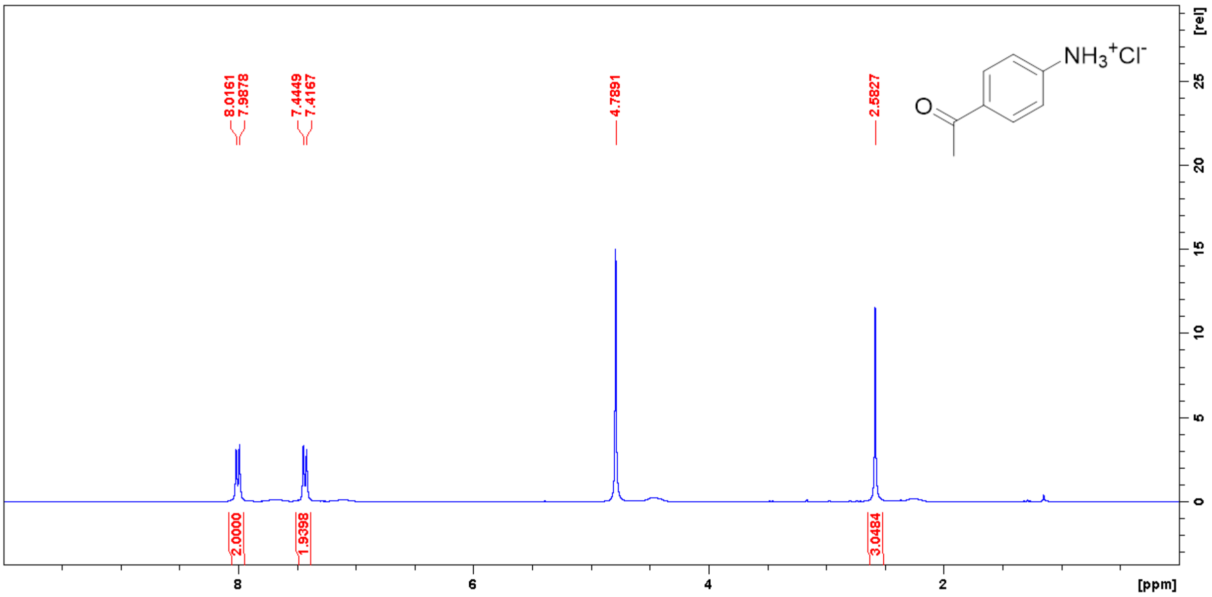

Figure S46: ^{1}H NMR Spectrum of 4-Acetylbenzenaminium Hydrochloride (**4l**) in D_2O.

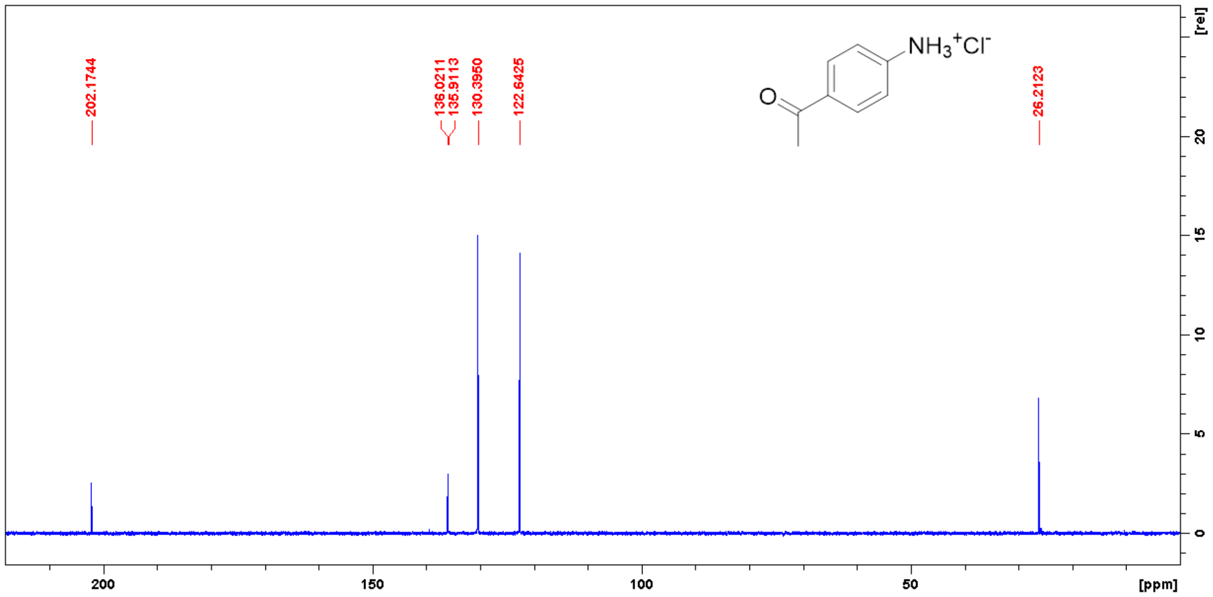

Figure S47: $^{13}C\{^1H\}$ NMR Spectrum of 4-Acetylbenzenaminium Hydrochloride (**4l**) in D_2O.

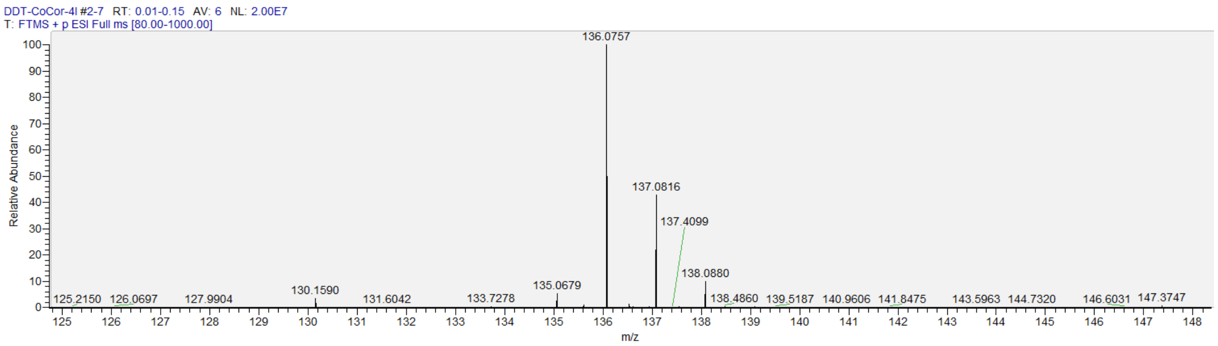

Figure S48: HR-MS of 4-Acetylbenzenaminium Hydrochloride (**4l**).

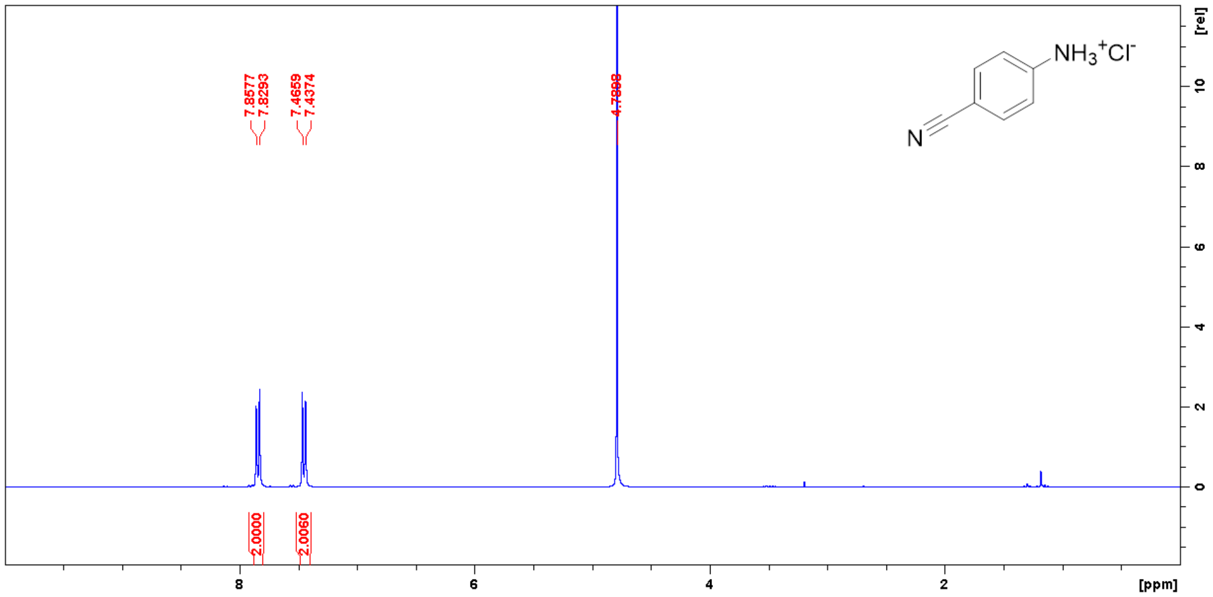

Figure S49: ^{1}H NMR Spectrum of 4-Cyanobenzenaminium Hydrochloride (**4m**) in D_2O.

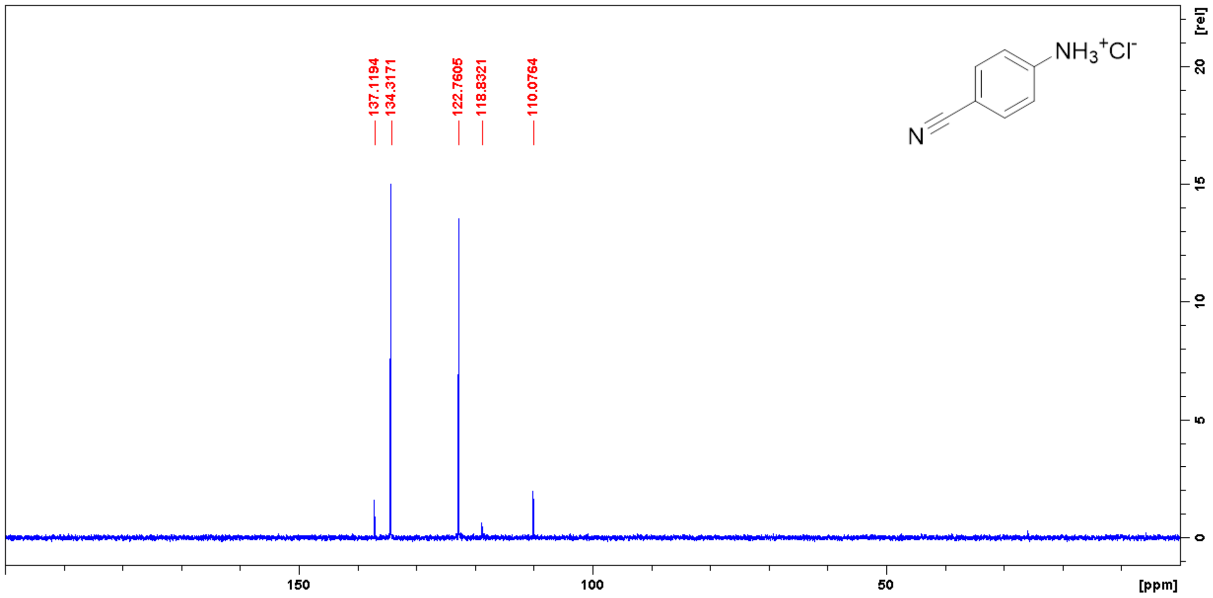

Figure S50: ^{13}C{^{1}H} NMR Spectrum of 4-Cyanobenzenaminium Hydrochloride (**4m**) in D_2O.

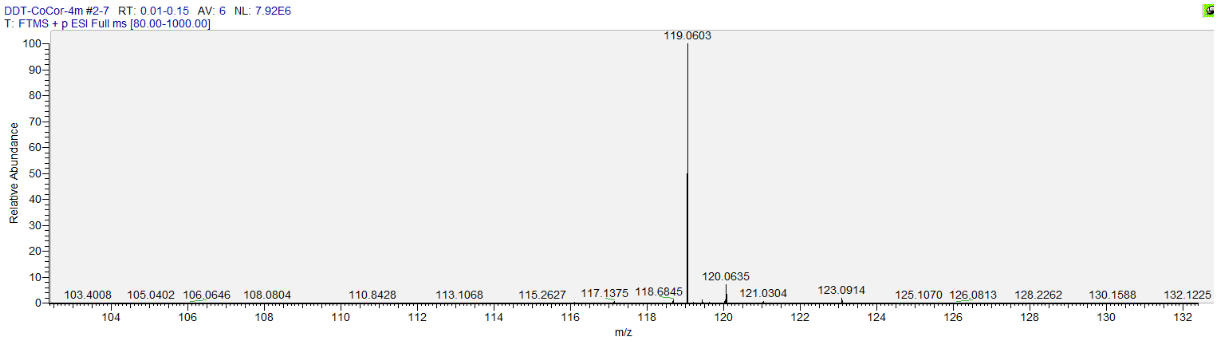

Figure S51: HR-MS of 4-Cyanobenzenaminium Hydrochloride (**4m**).

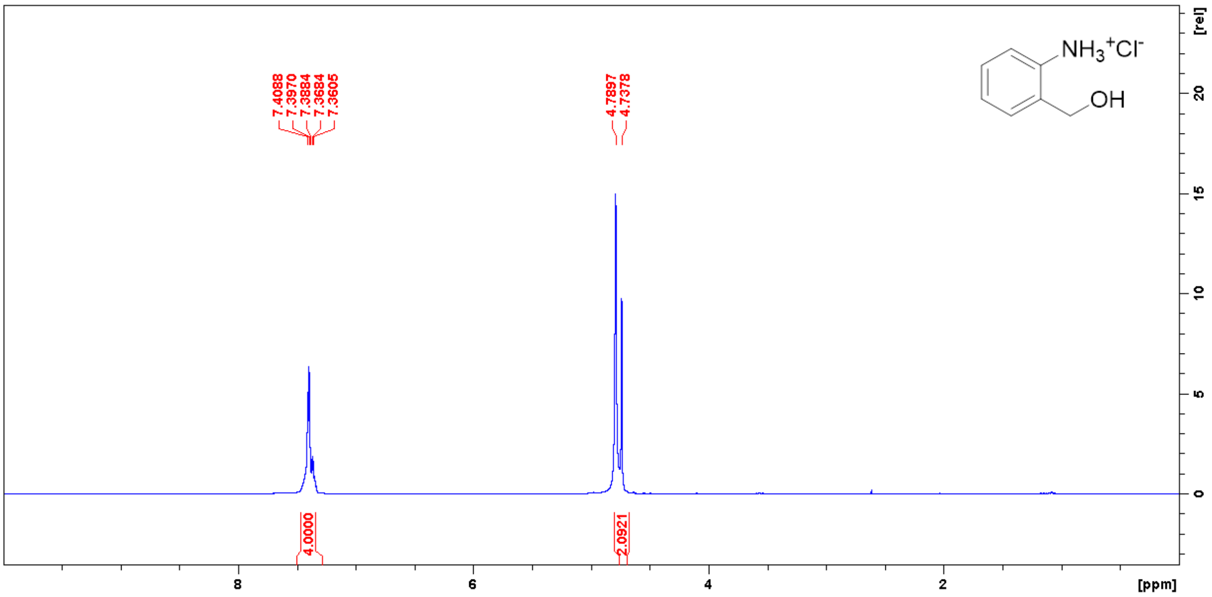

Figure S52: ^{1}H NMR Spectrum of 2-(Hydroxymethyl)benzenaminium Hydrochloride (**4n**) in D_2O.

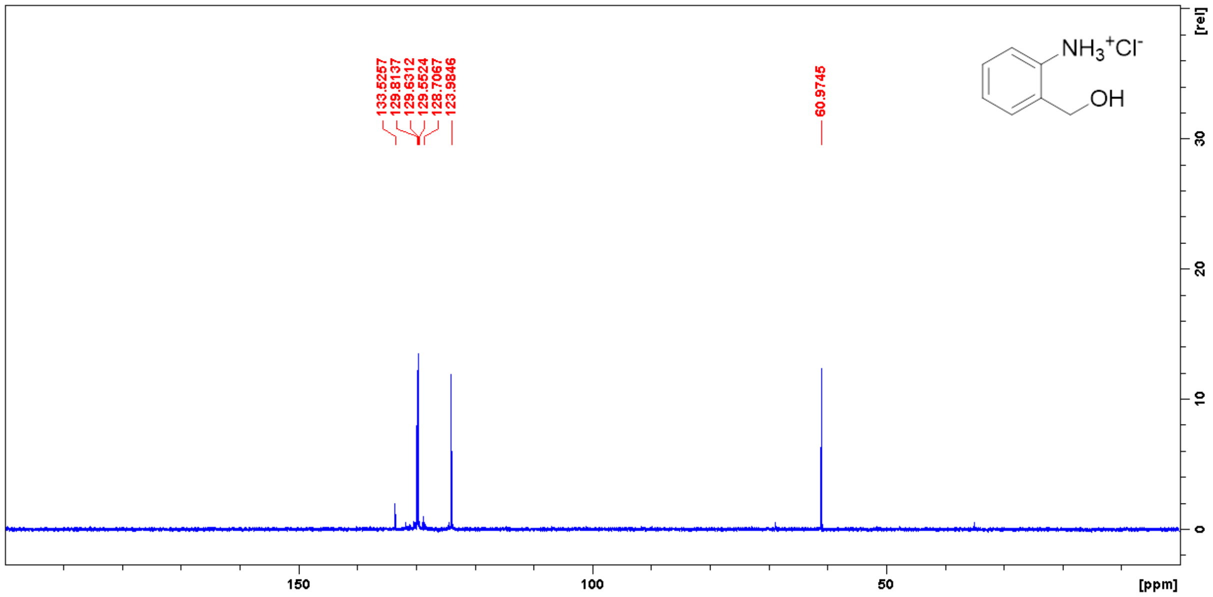

Figure S53: $^{13}C\{^1H\}$ NMR Spectrum of 2-(Hydroxymethyl)benzenaminium Hydrochloride (**4n**) in D_2O.

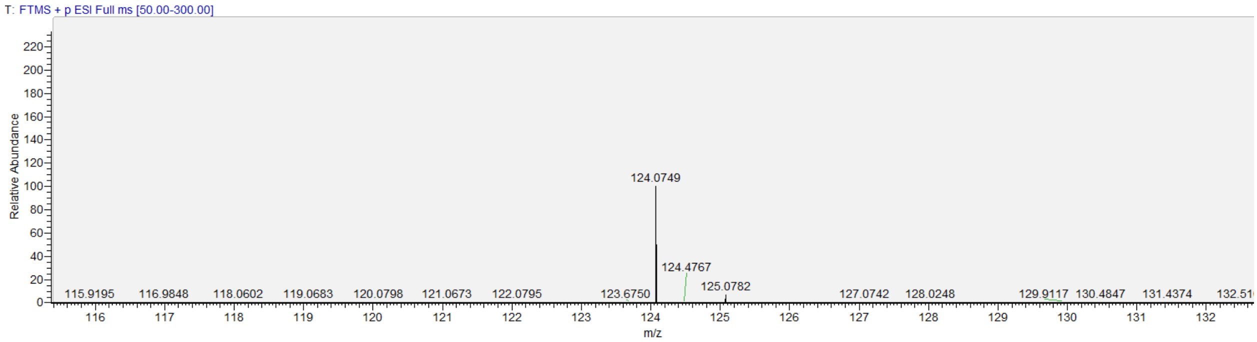

Figure S54: HR-MS of 2-(Hydroxymethyl)benzenaminium Hydrochloride (**4n**).

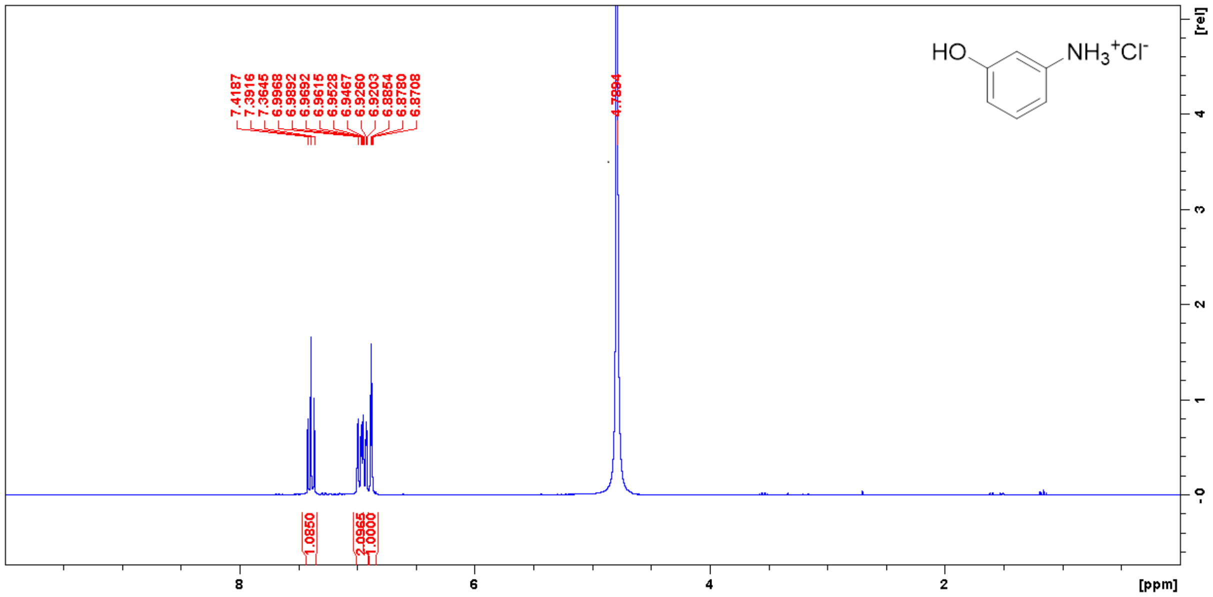

Figure S55: ^{1}H NMR Spectrum of 3-Hydroxybenzenaminium Hydrochloride (**4o**) in D_2O.

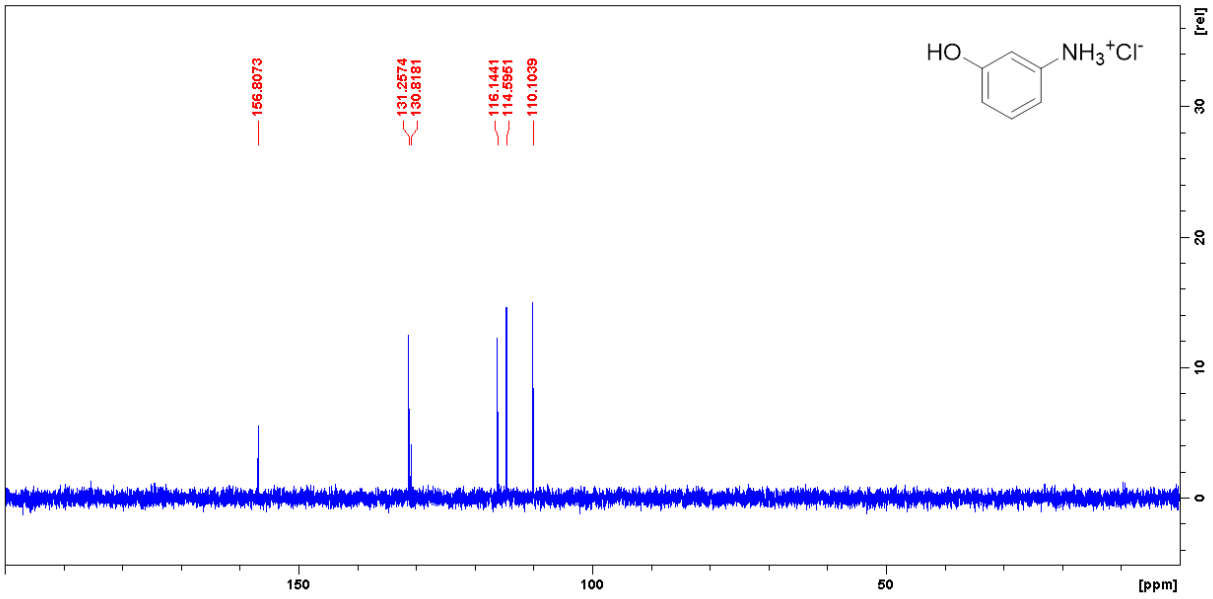

Figure S56: $^{13}C\{^{1}H\}$ NMR Spectrum of 3-Hydroxybenzenaminium Hydrochloride (**4o**) in D_2O.

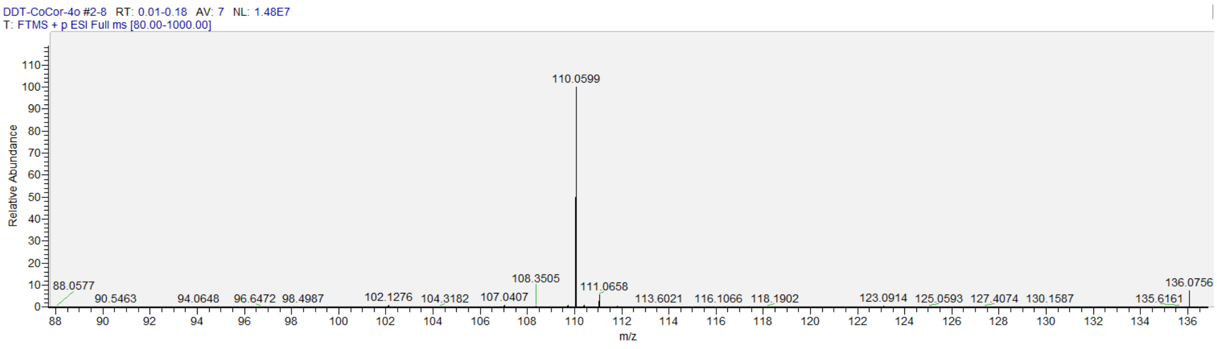

Figure S57: HR-MS of 3-Hydroxybenzenaminium Hydrochloride (**4o**).

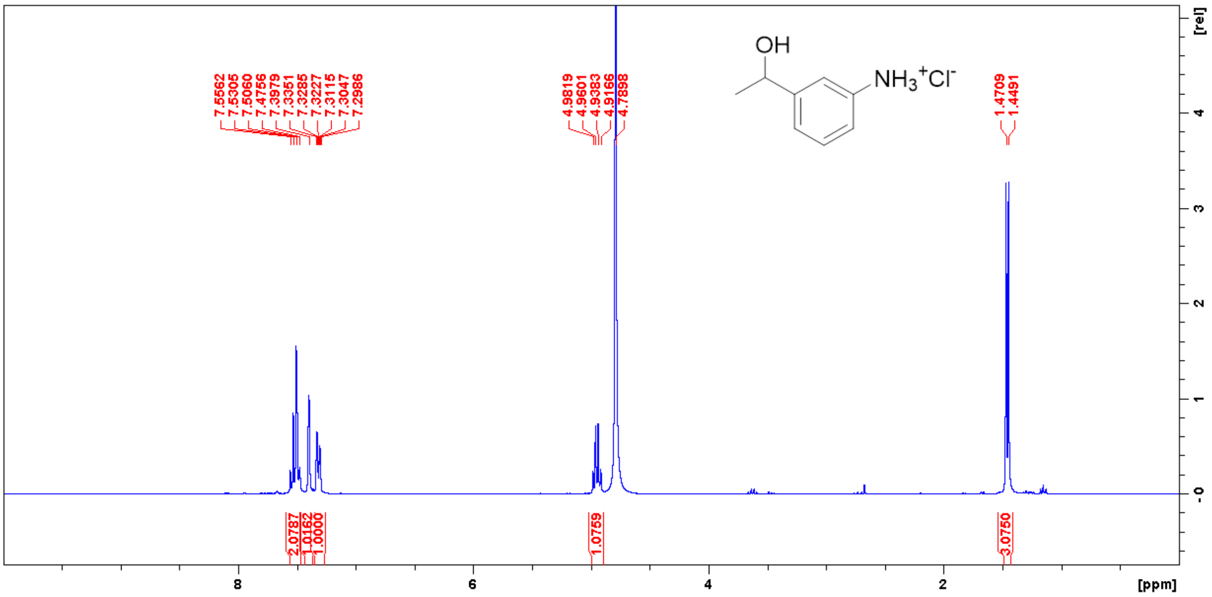

Figure S58: ^{1}H NMR Spectrum of 3-(1-Hydroxyethyl)benzenaminium Hydrochloride (**4p**) in D_2O.

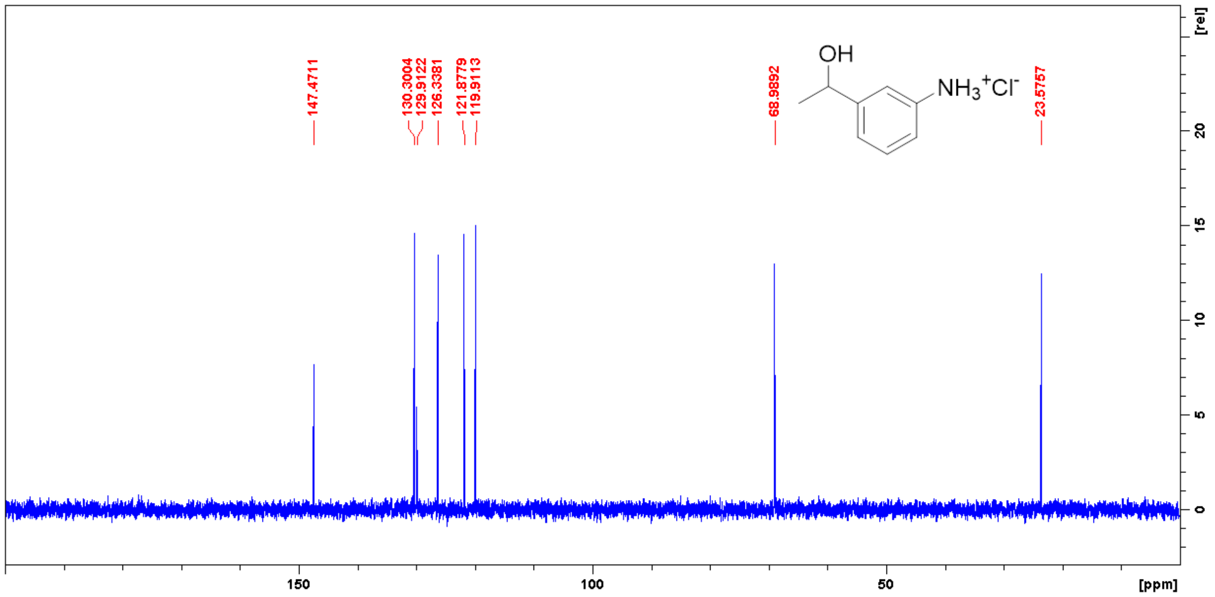

Figure S59: $^{13}C\{^{1}H\}$ NMR Spectrum of 3-(1-Hydroxyethyl)benzenaminium Hydrochloride (**4p**) in D_2O.

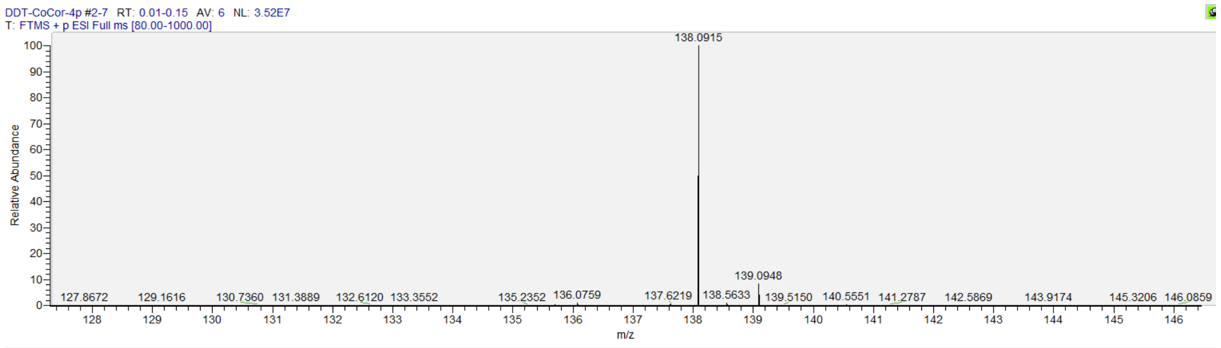

Figure S60: HR-MS of 3-(1-Hydroxyethyl)benzenaminium Hydrochloride (**4p**).

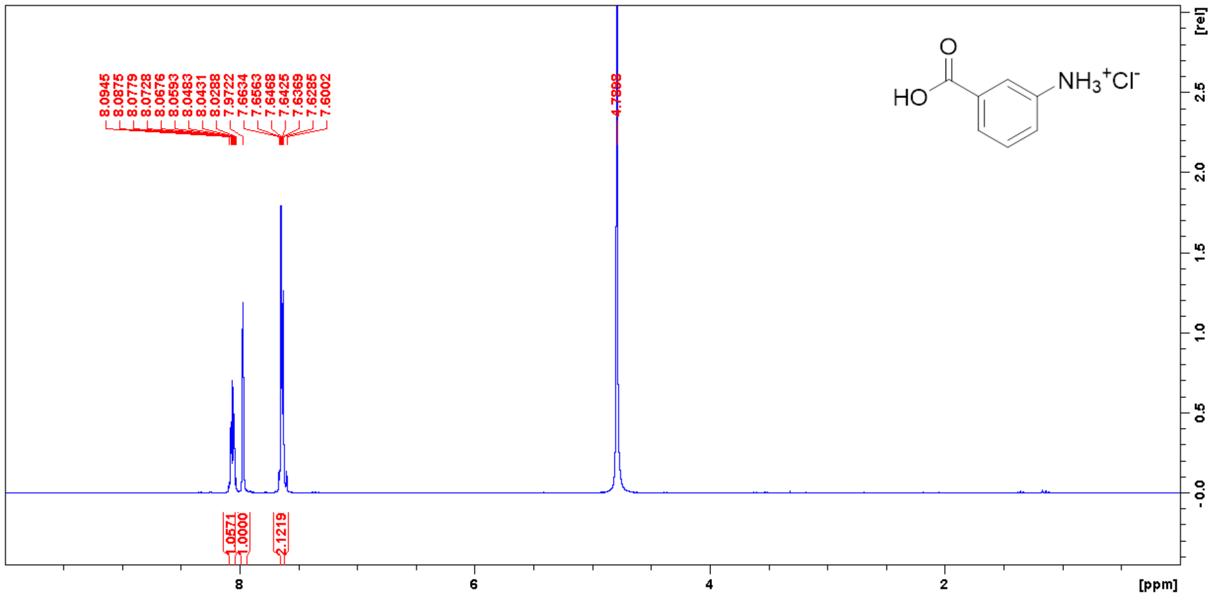

Figure S61: ^{1}H NMR Spectrum of 3-Carboxybenzenaminium Hydrochloride (**4q**) in D_2O.

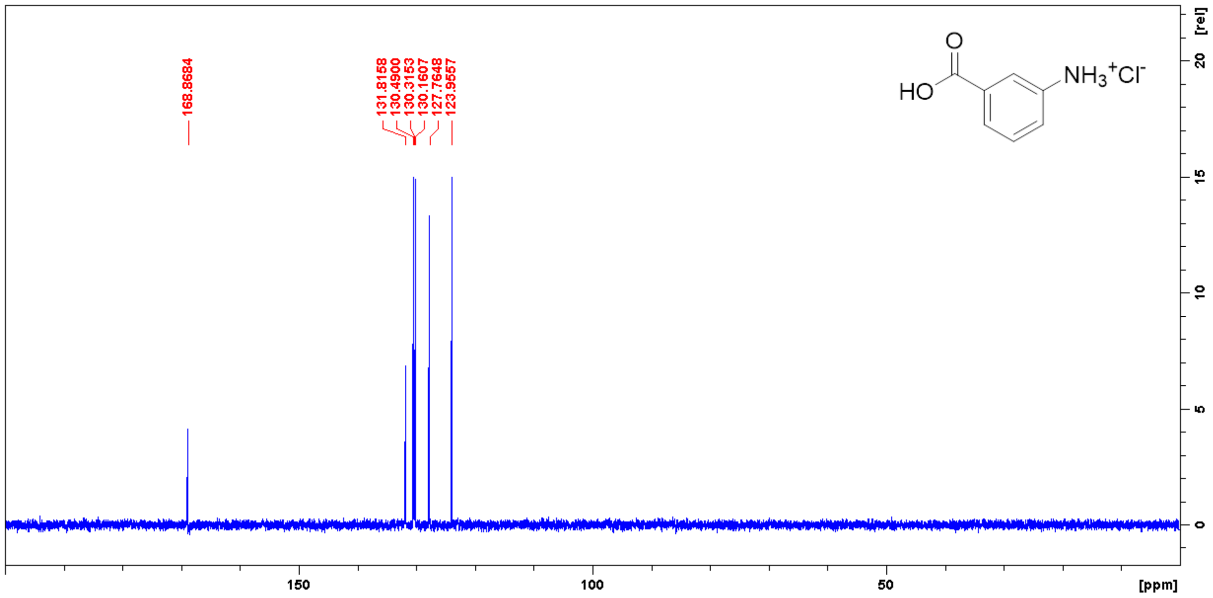

Figure S62: $^{13}C\{^{1}H\}$ NMR Spectrum of 3-Carboxybenzenaminium Hydrochloride (**4q**) in D_2O.

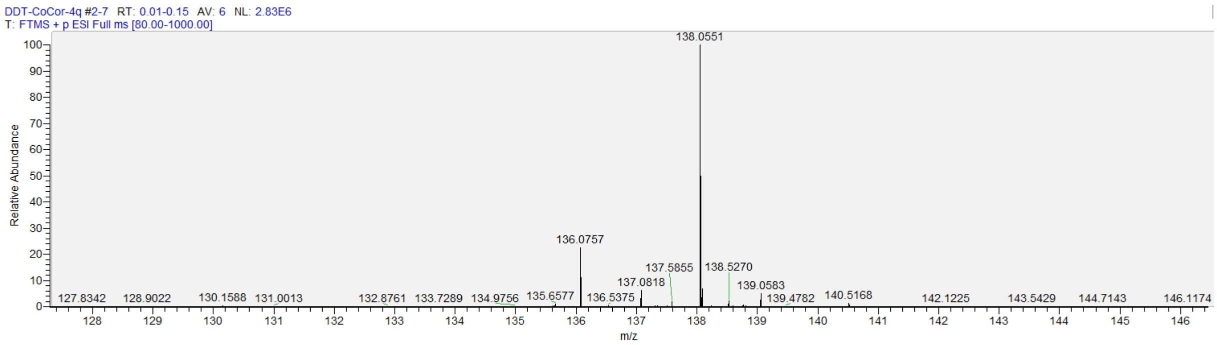

Figure S63: HR-MS of 3-Carboxybenzenaminium Hydrochloride (**4q**).

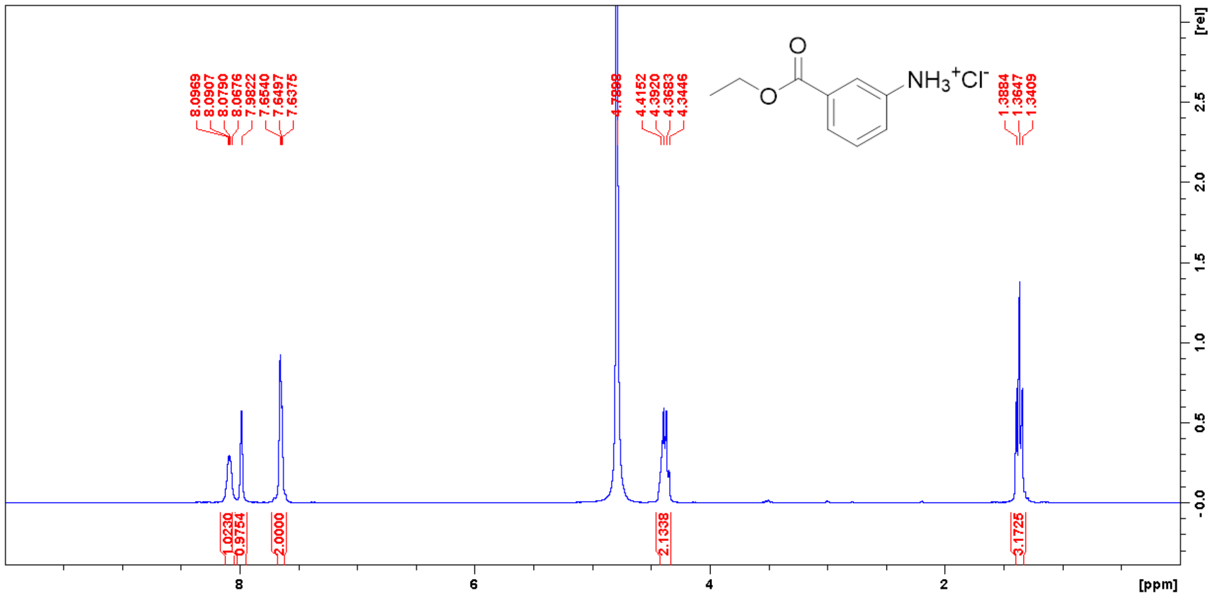

Figure S64: ^{1}H NMR Spectrum of 3-(Ethoxycarbonyl)benzenaminium Hydrochloride (**4r**) in D_2O.

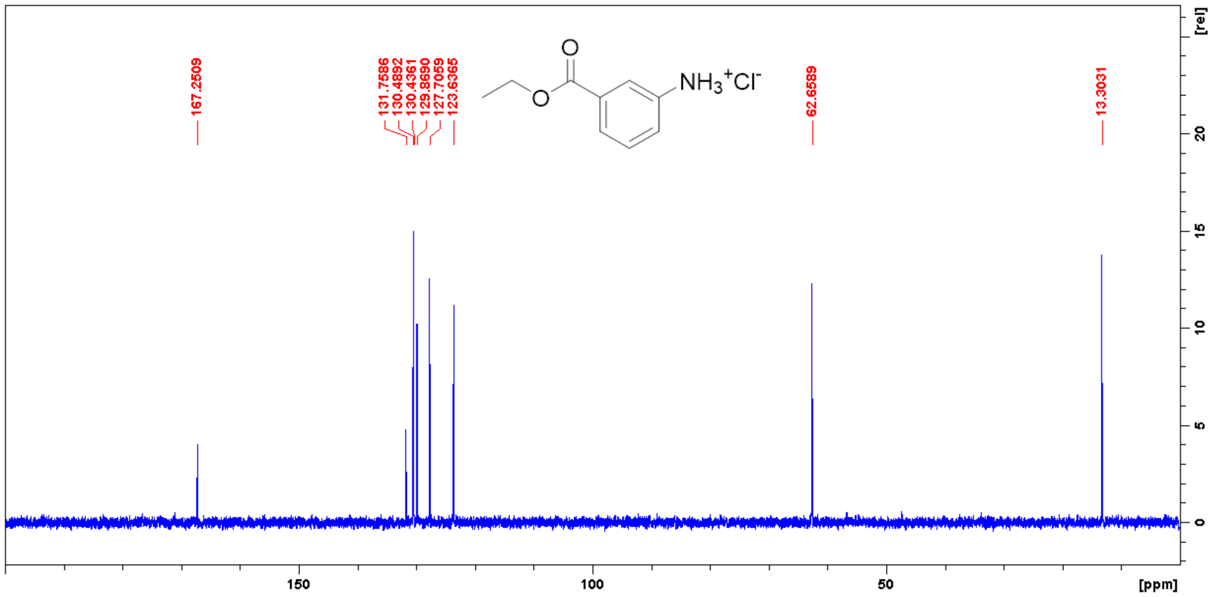

Figure S65: $^{13}C\{^1H\}$ NMR Spectrum of 3-(Ethoxycarbonyl)benzenaminium Hydrochloride (**4r**) in D_2O.

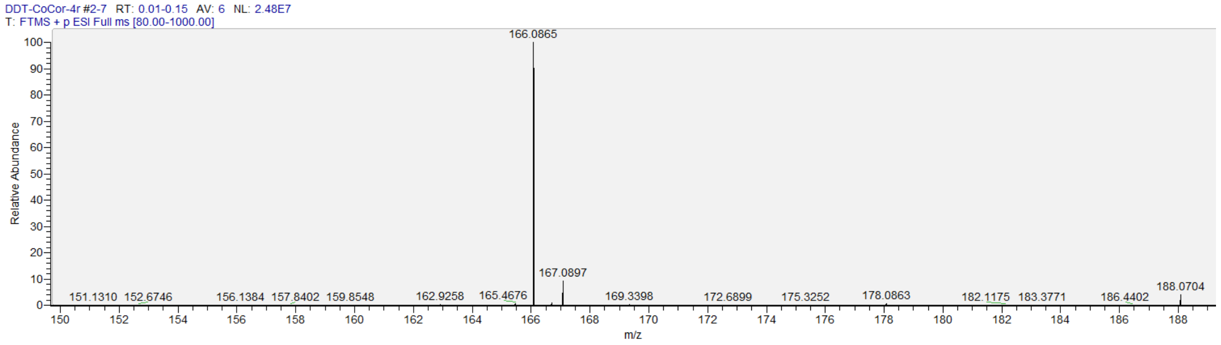

Figure S66: HR-MS of 3-(Ethoxycarbonyl)benzenaminium Hydrochloride (**4r**).

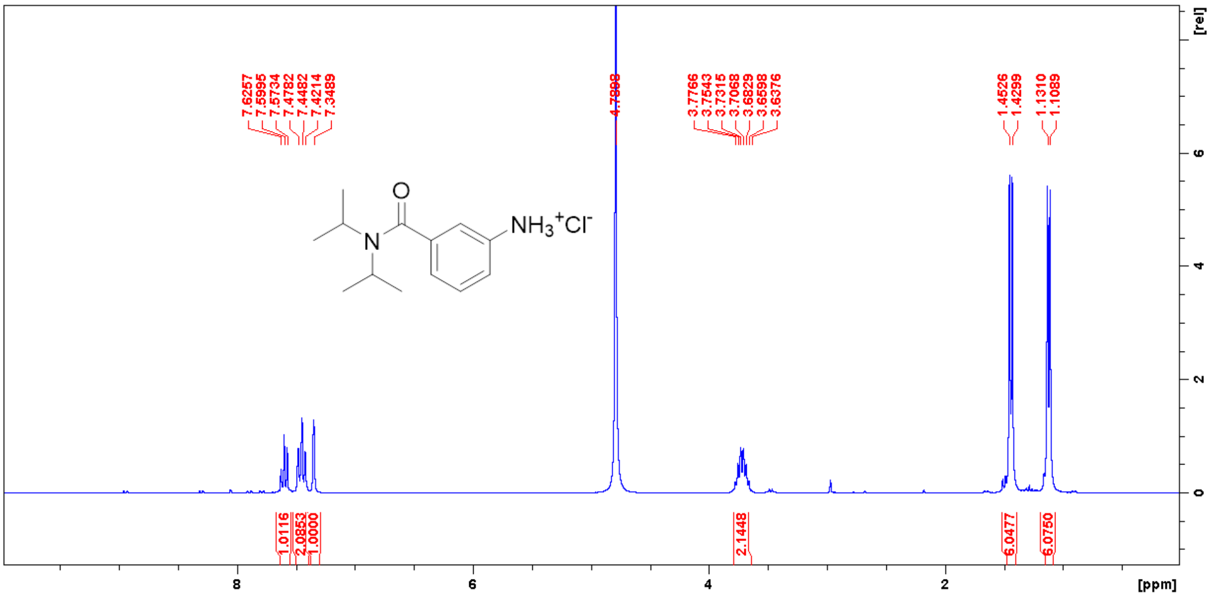

Figure S67: ^{1}H NMR Spectrum of 3-(Diisopropylcarbamoyl)benzenaminium Hydrochloride (**4s**) in D_2O.

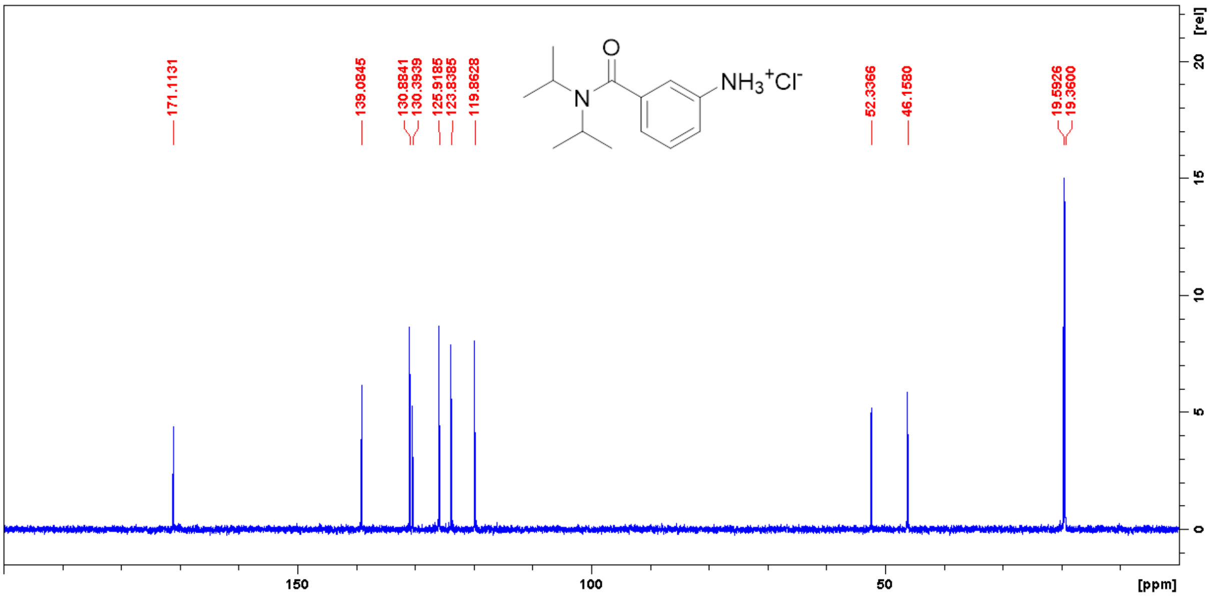

Figure S68: ^{13}C{^{1}H} NMR Spectrum of 3-(Diisopropylcarbamoyl)benzenaminium Hydrochloride (**4s**) in D_2O.

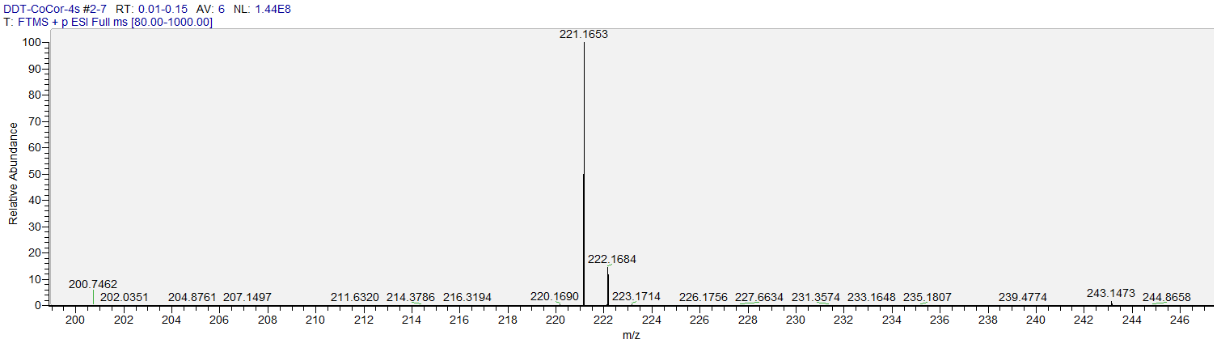

Figure S69: HR-MS of 3-(Diisopropylcarbamoyl)benzenaminium Hydrochloride (**4s**).

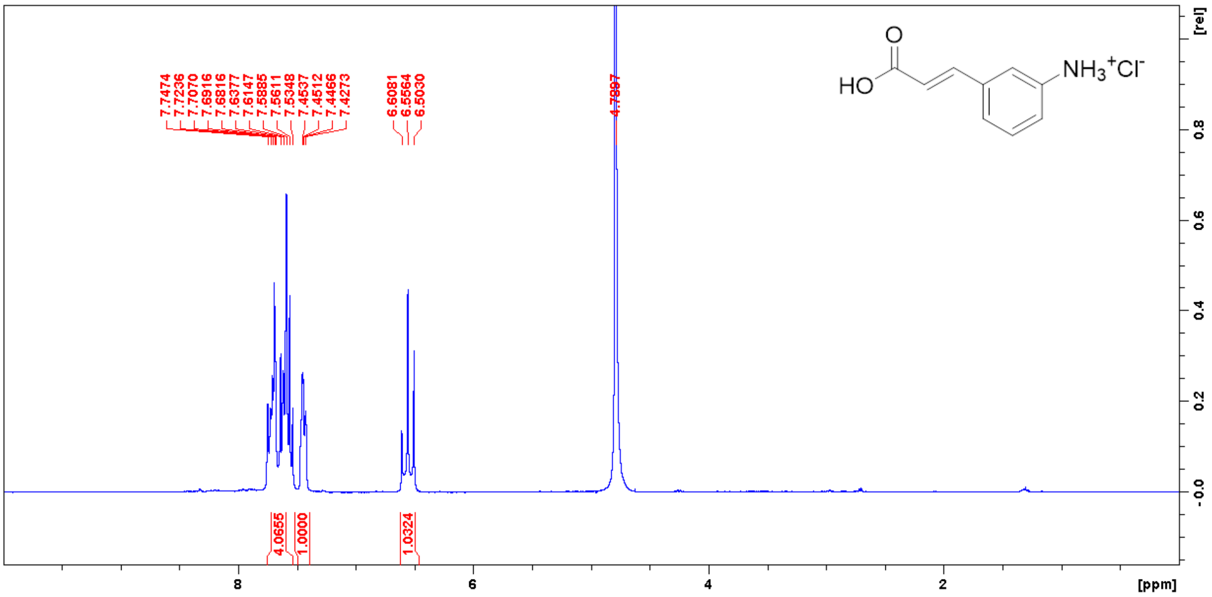

Figure S70: ^{1}H NMR Spectrum of 3-(2-Carboxyvinyl)benzenaminium Hydrochloride (**4t**) in D_2O.

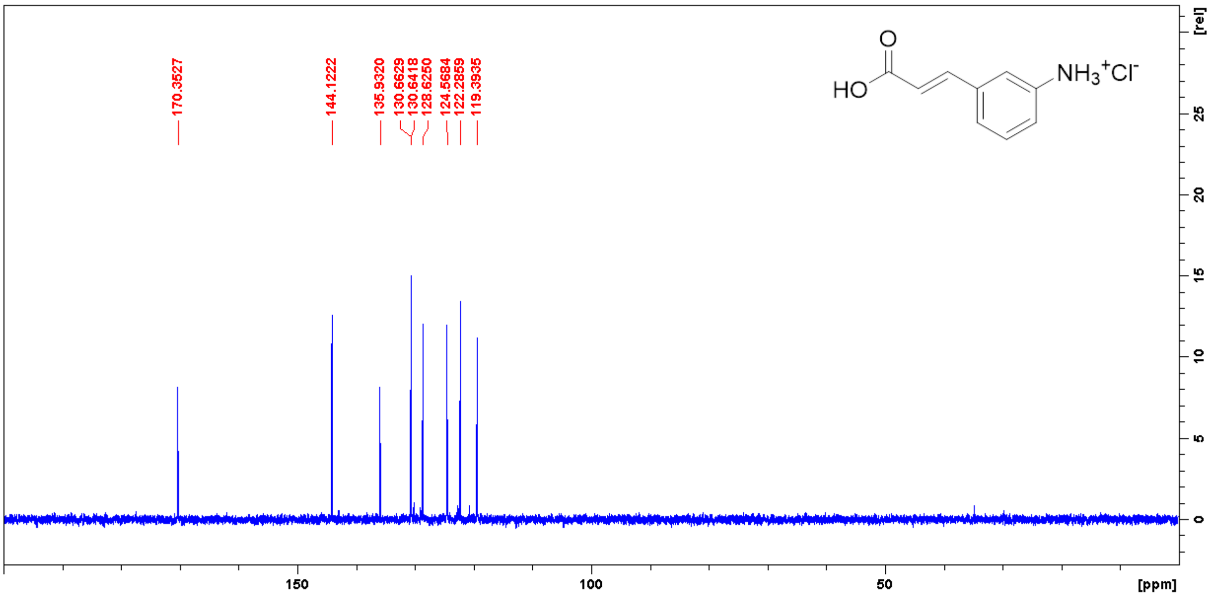

Figure S71: $^{13}C\{^{1}H\}$ NMR Spectrum of 3-(2-Carboxyvinyl)benzenaminium Hydrochloride (**4t**) in D_2O.

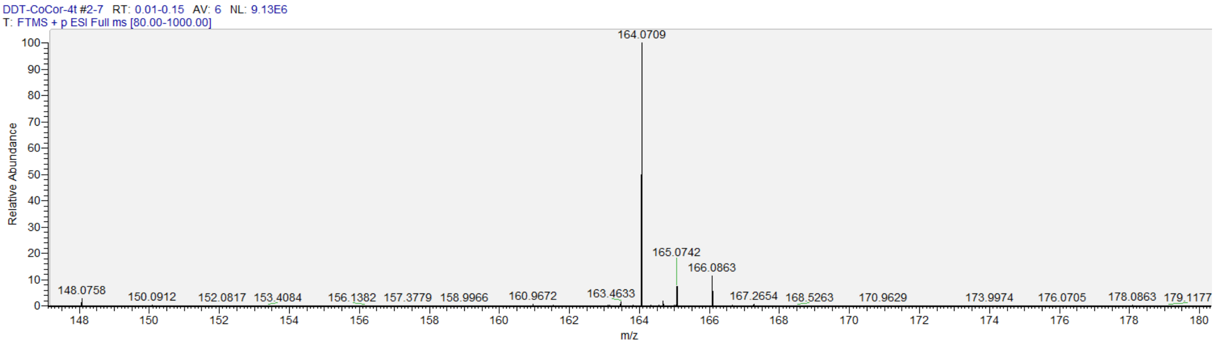

Figure S72: HR-MS of 3-(2-Carboxyvinyl)benzenaminium Hydrochloride (**4t**).

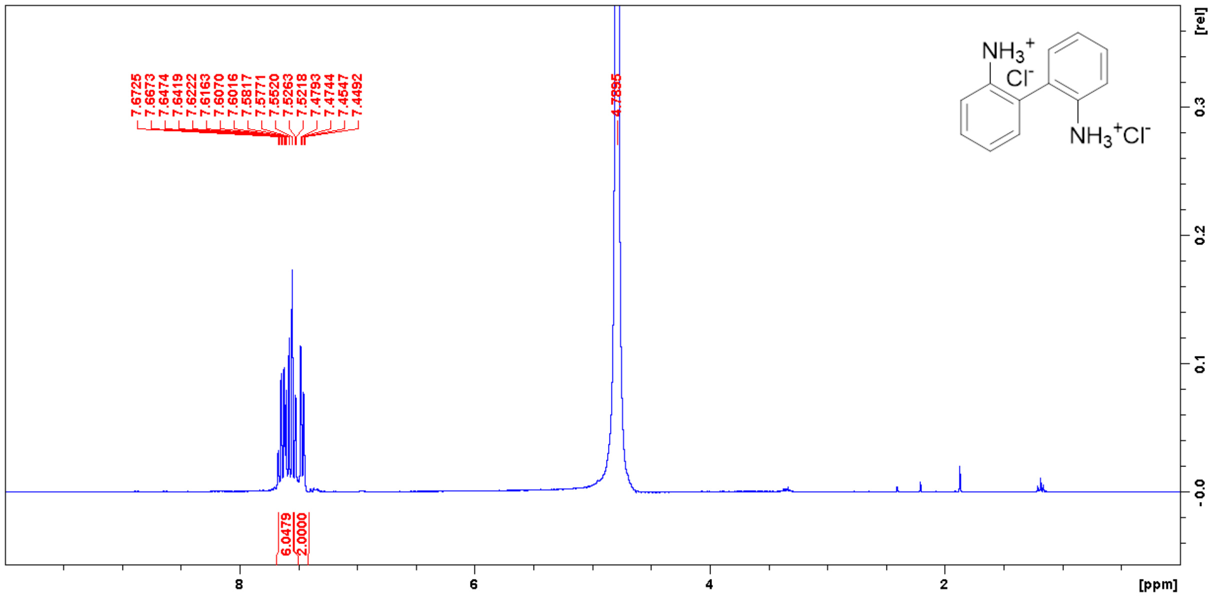

Figure S73: ^{1}H NMR Spectrum of 1,1'-Biphenyl-2,2'-diaminium Hydrochloride (**4u**) in D_2O.

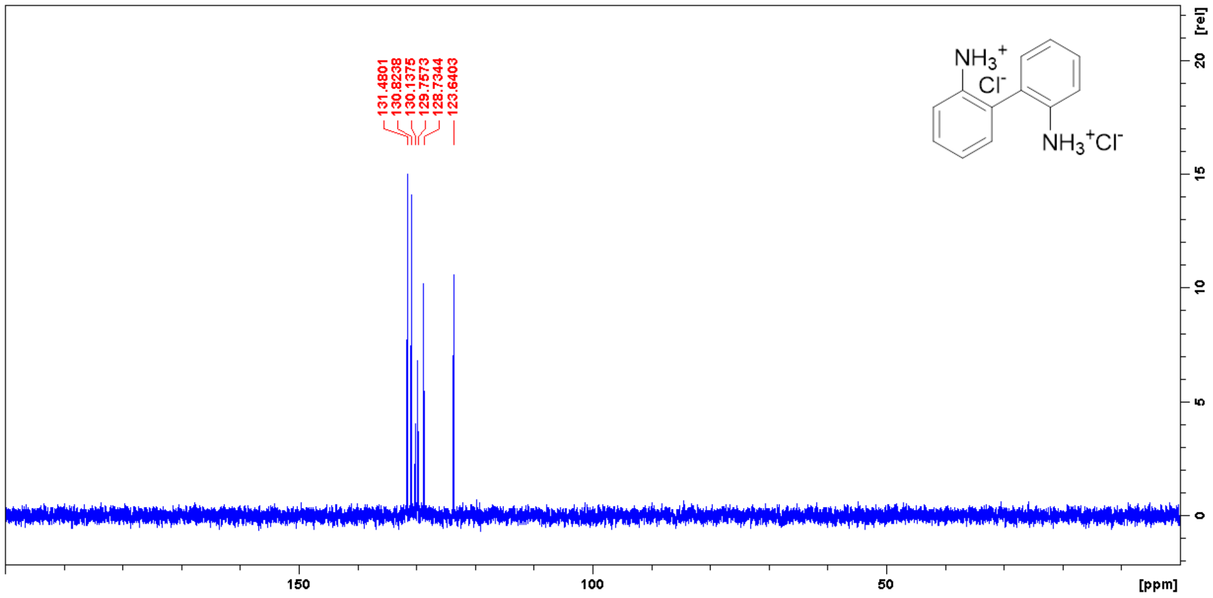

Figure S74: ^{13}C{^{1}H} NMR Spectrum of 1,1'-Biphenyl-2,2'-diaminium Hydrochloride (**4u**) in D_2O.

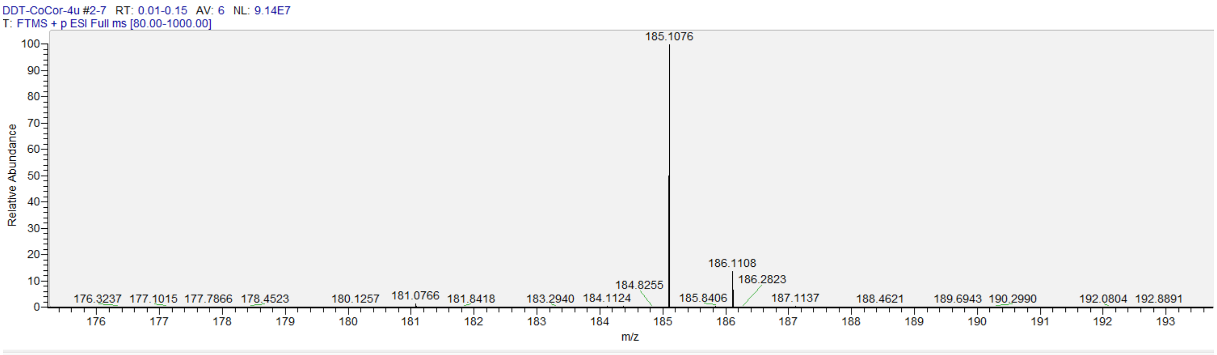

Figure S75: HR-MS of 1,1'-Biphenyl-2,2'-diaminium Hydrochloride (**4u**).

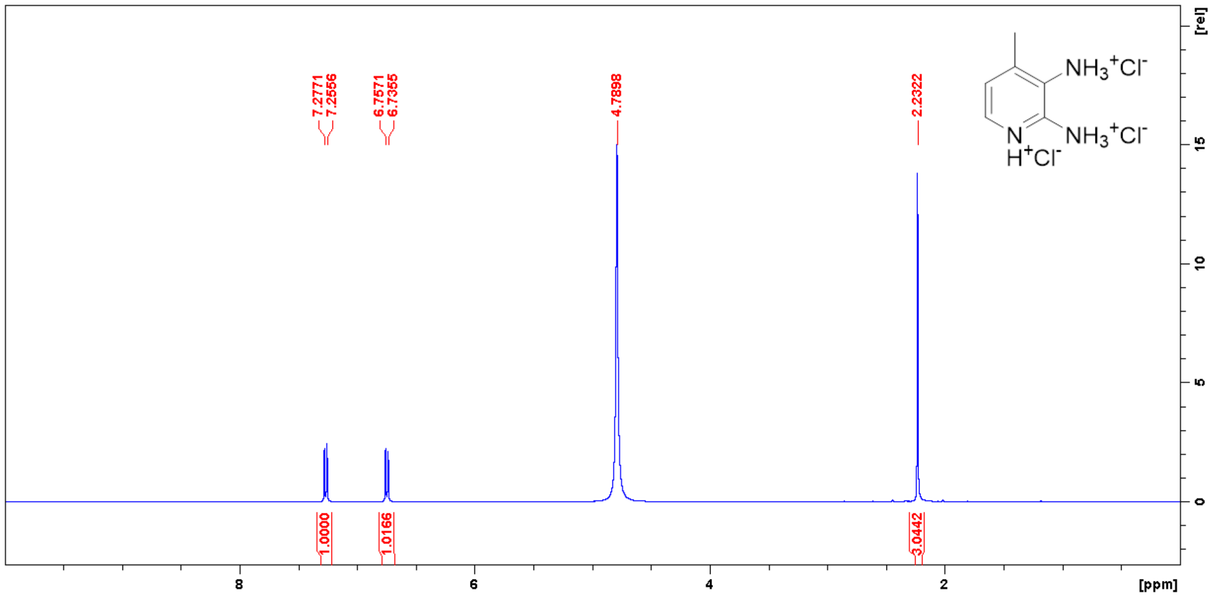

Figure S76: ^{1}H NMR Spectrum of 2,3-Diammonio-4-methylpyridinium Hydrochloride (**4v**) in D_2O.

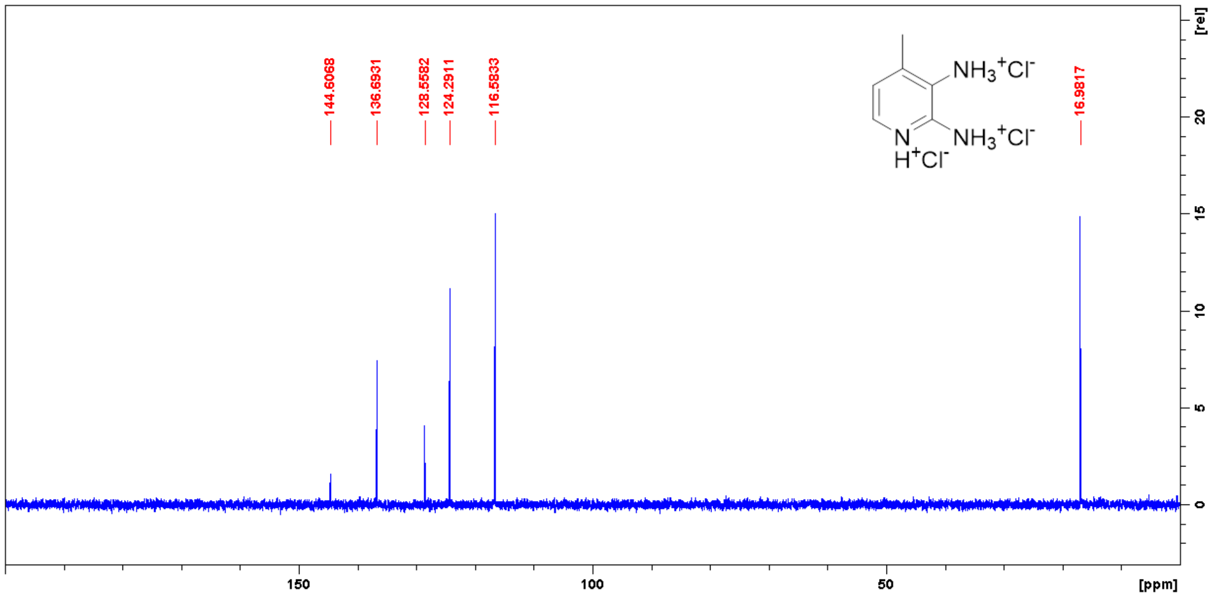

Figure S77: $^{13}C\{^{1}H\}$ NMR Spectrum of 2,3-Diammonio-4-methylpyridinium Hydrochloride (**4v**) in D_2O.

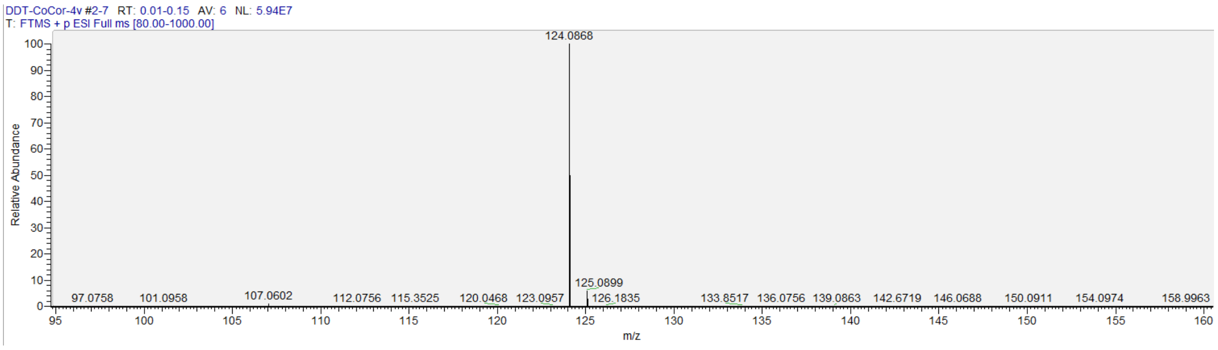

Figure S78: HR-MS of 2,3-Diammonio-4-methylpyridinium Hydrochloride (**4v**).

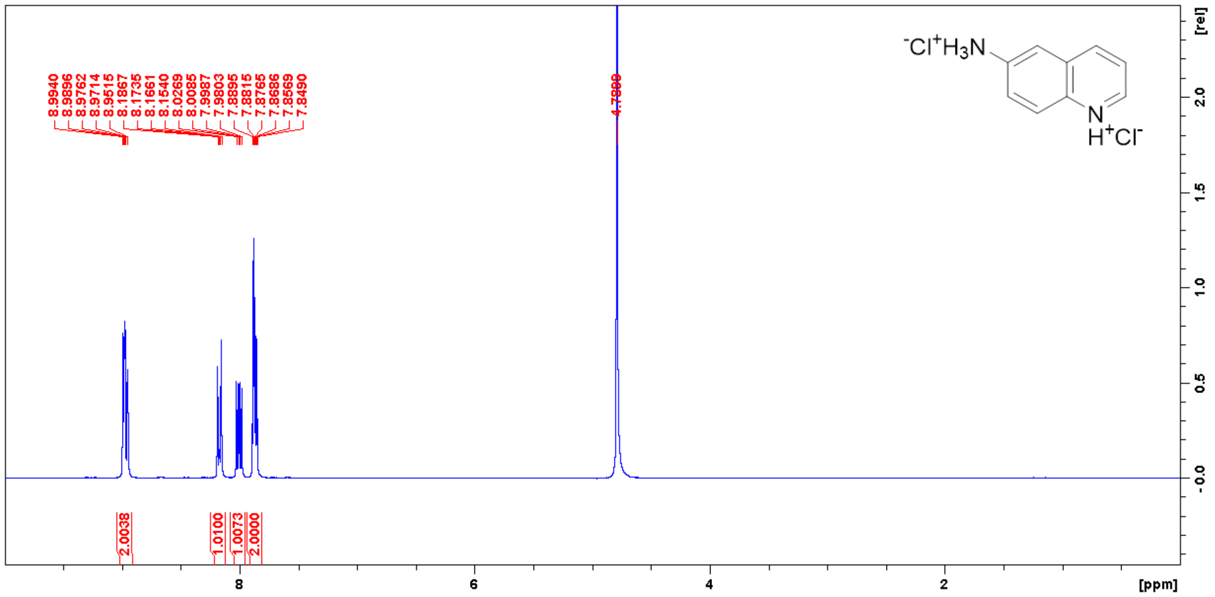

Figure S79: ^{1}H NMR Spectrum of 6-Ammonioquinolinium Hydrochloride (**4w**) in D_2O.

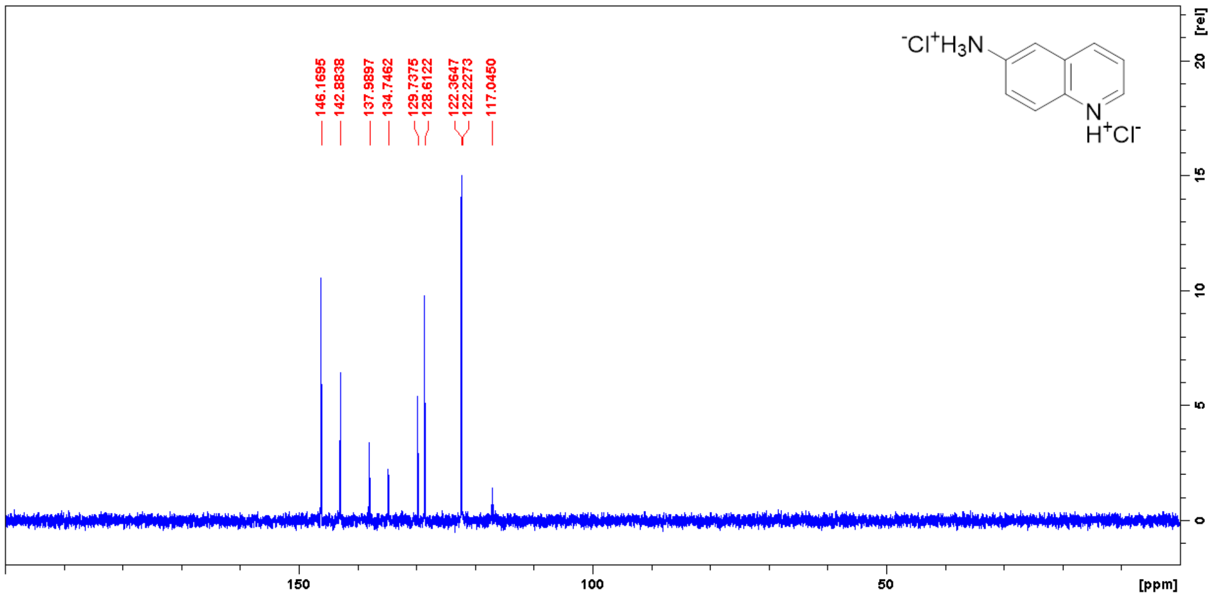

Figure S80: ^{13}C{^{1}H} NMR Spectrum of 6-Ammonioquinolinium Hydrochloride (**4w**) in D_2O.

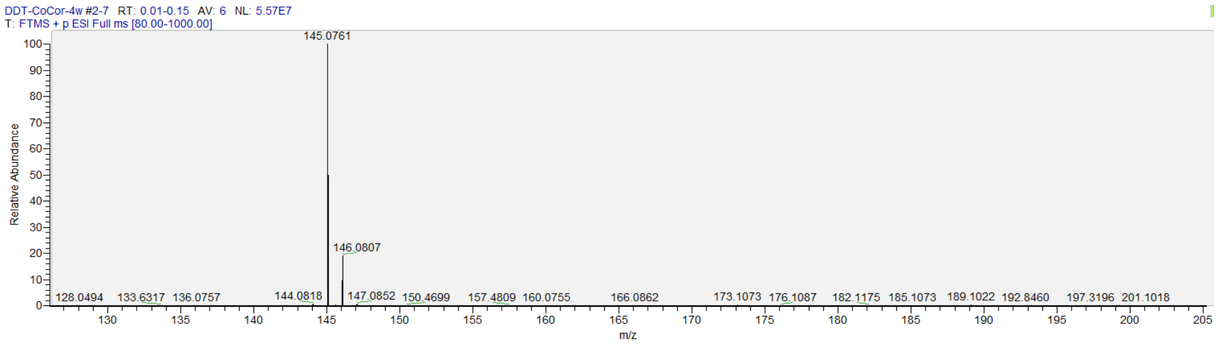

Figure S81: HR-MS of 6-Ammonioquinolinium Hydrochloride (**4w**).

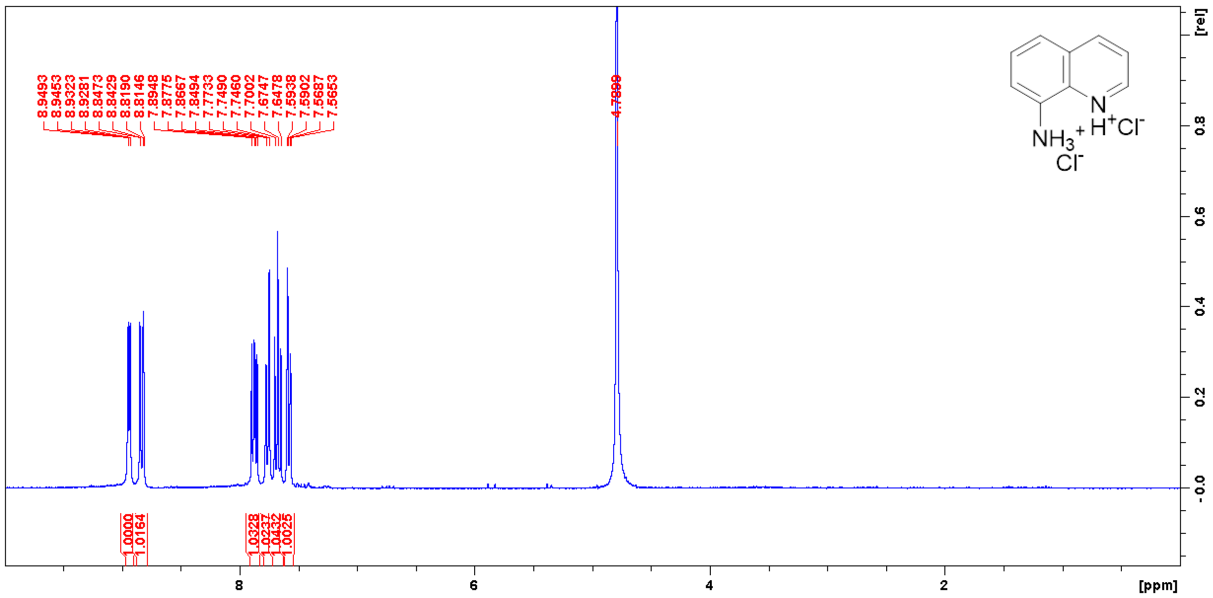

Figure S82: ^{1}H NMR Spectrum of 8-Ammonioquinolinium Hydrochloride (**4x**) in D_2O.

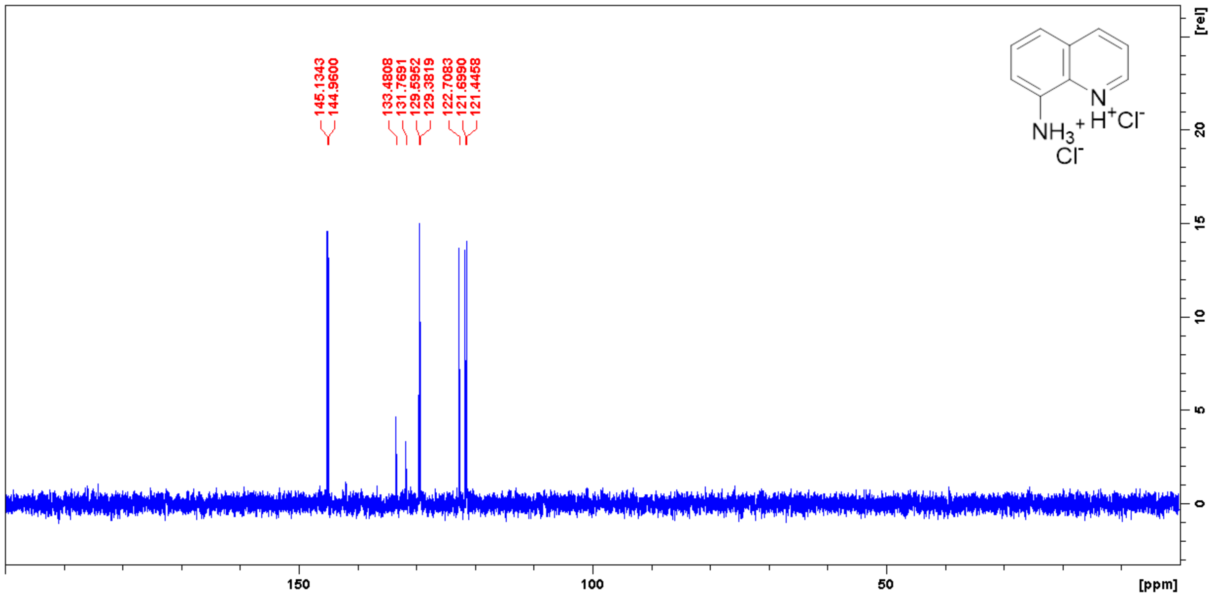

Figure S83: $^{13}C\{^{1}H\}$ NMR Spectrum of 8-Ammonioquinolinium Hydrochloride (**4x**) in D_2O.

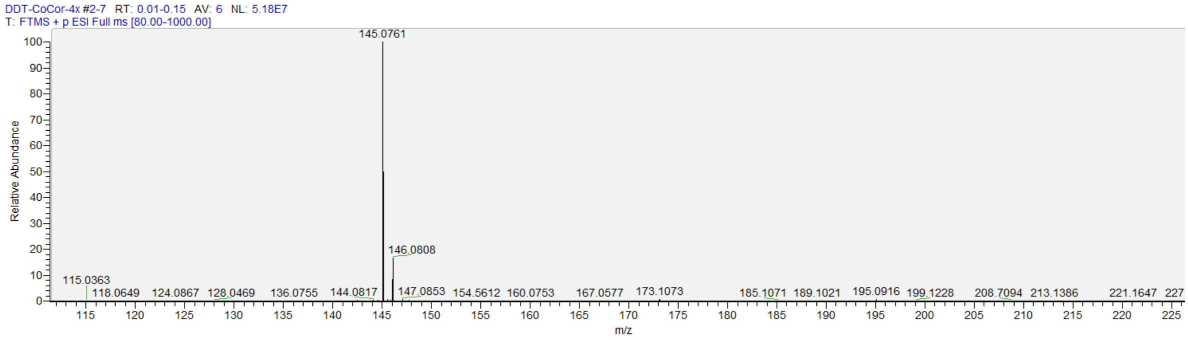

Figure S84: HR-MS of 8-Ammonioquinolinium Hydrochloride (**4x**).

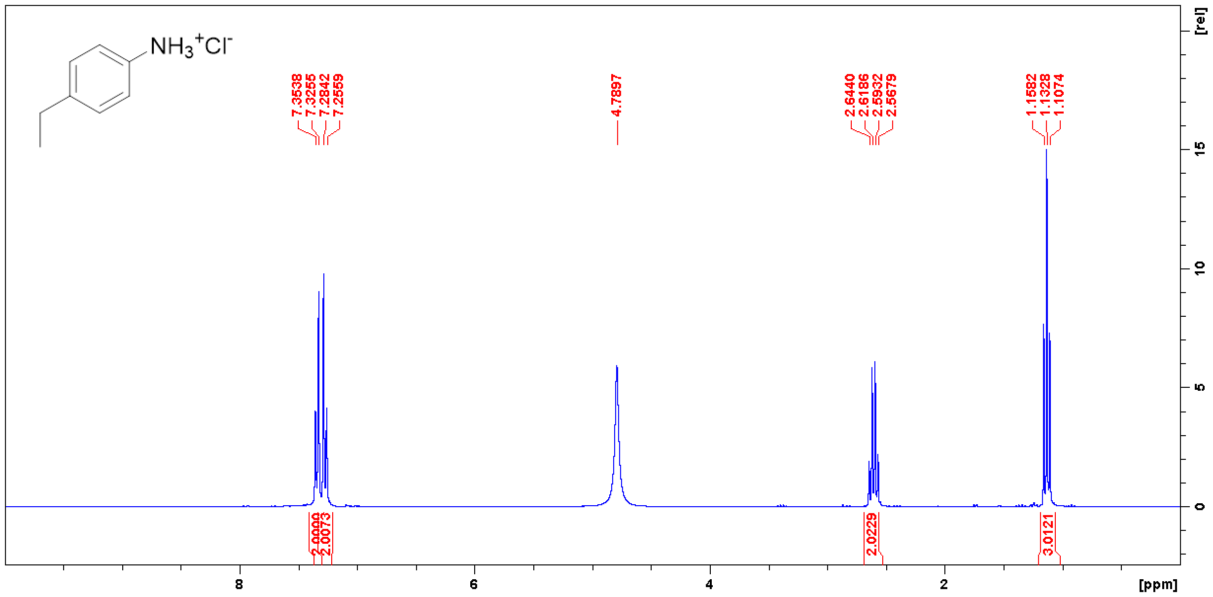

Figure S85: ^{1}H NMR Spectrum of 4-Ethylanilinium Hydrochloride (**4y**) in D_2O.

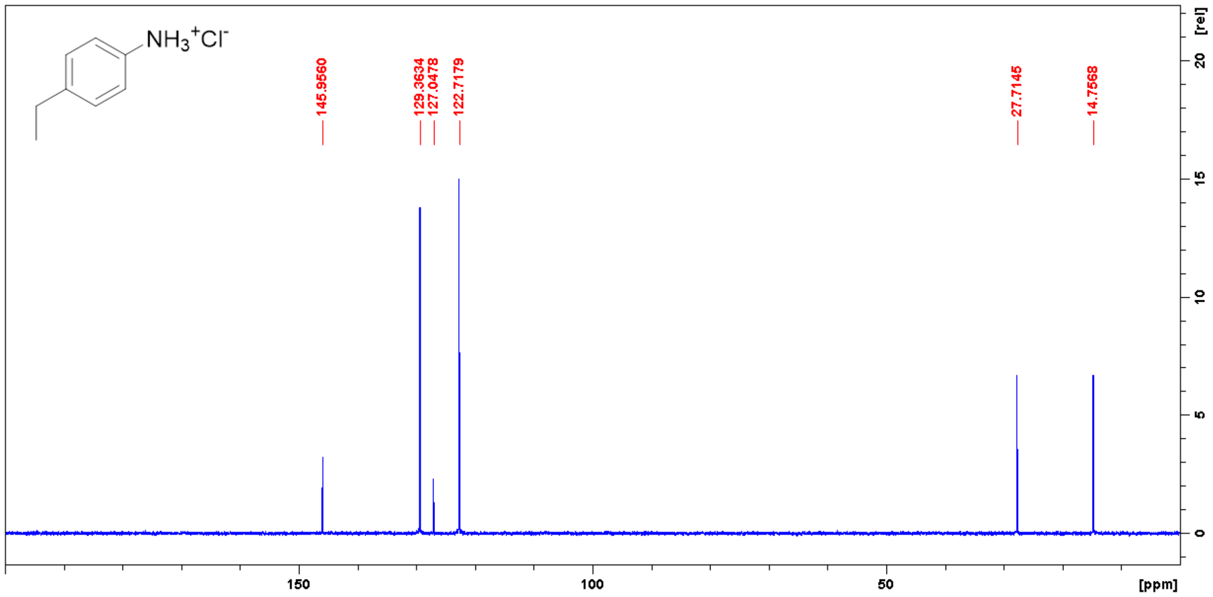

Figure S86: $^{13}C\{^{1}H\}$ NMR Spectrum of 4-Ethylanilinium Hydrochloride (**4y**) in D_2O.

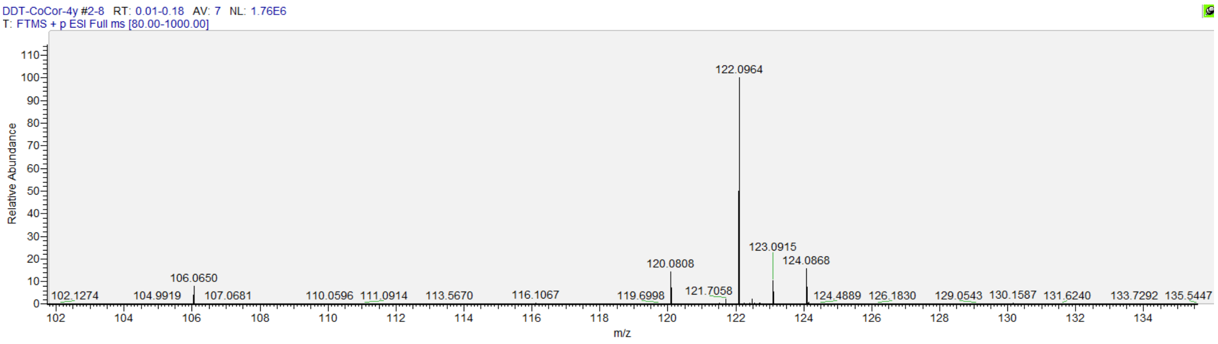

Figure S87: HR-MS of 4-Ethylanilinium Hydrochloride (**4y**).

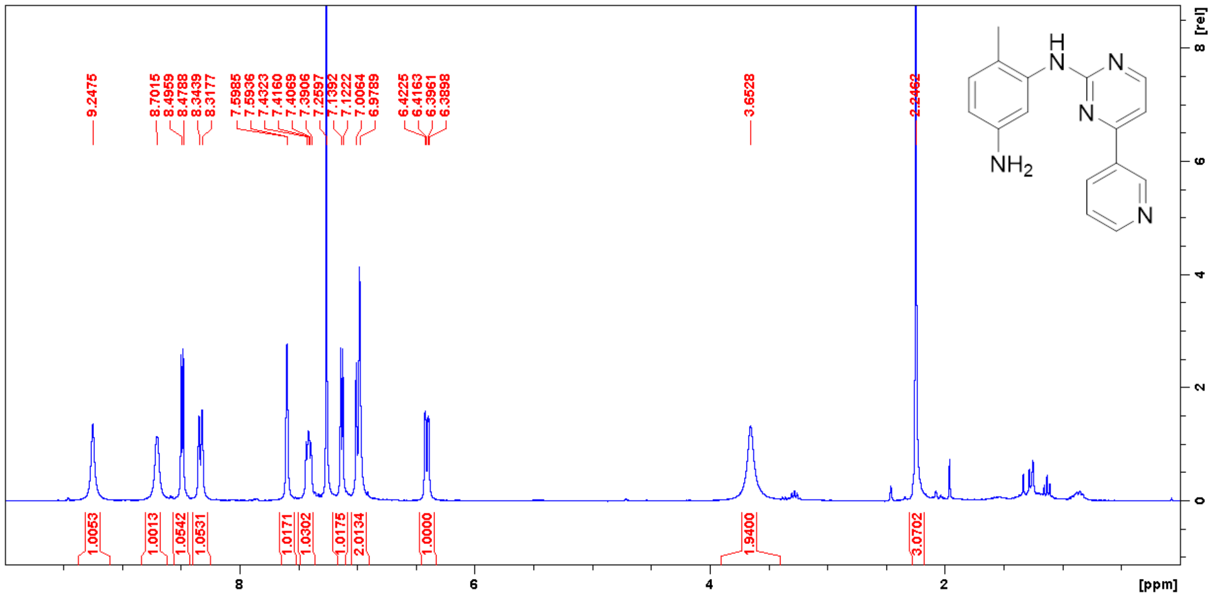

Figure S88: ^{1}H NMR Spectrum of 6-Methyl-N[1]-(4-(pyridin-3-yl)pyrimidin-2-yl)benzene-1,3-diamine (**3aa**) in $CDCl_3$.

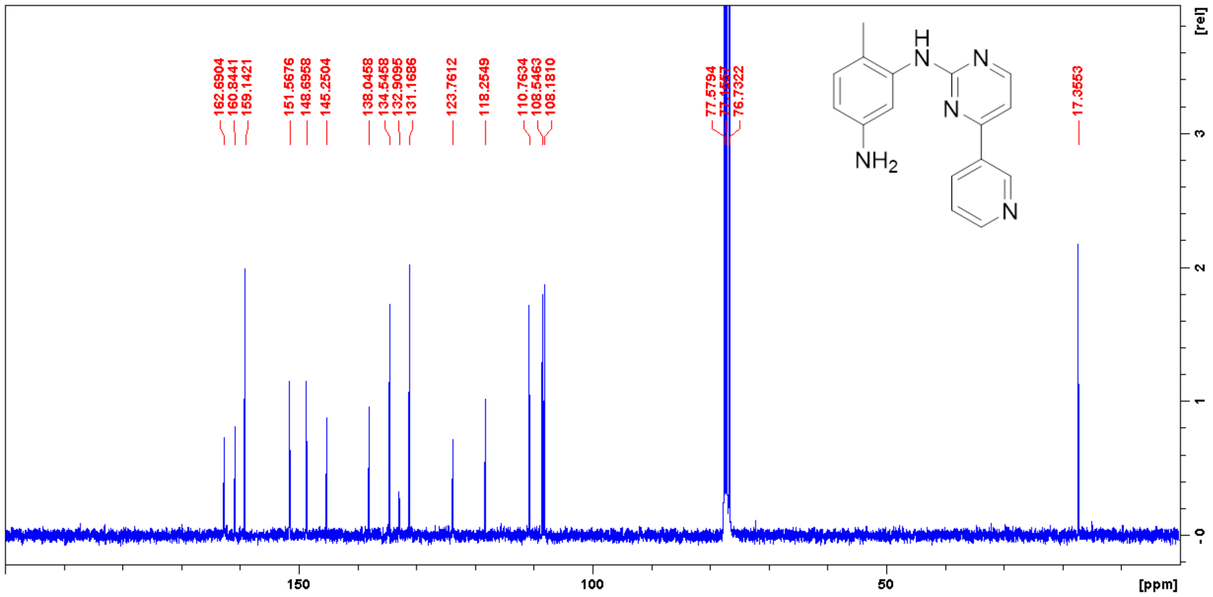

Figure S89: $^{13}C\{^{1}H\}$ NMR spectrum of 6-Methyl-N^{1}-(4-(pyridin-3-yl)pyrimidin-2-yl)benzene-1,3-diamine (**3aa**) in $CDCl_3$.

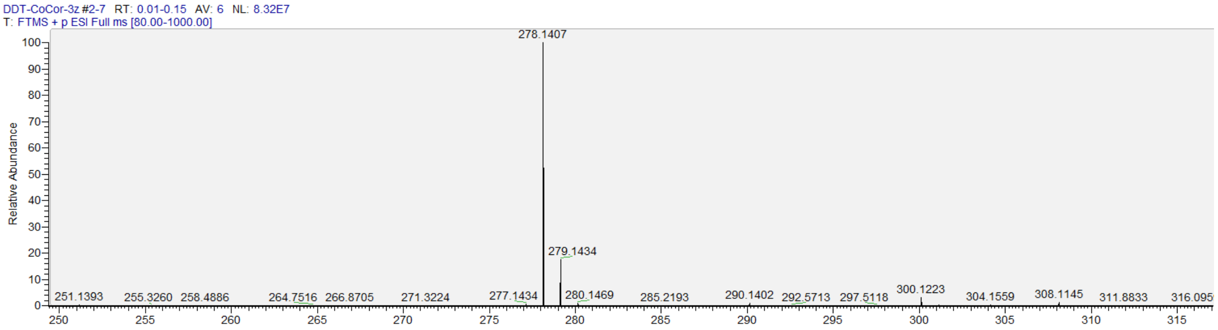

Figure S90: HR-MS of 6-Methyl-N^{1}-(4-(pyridin-3-yl)pyrimidin-2-yl)benzene-1,3-diamine (**3aa**).
